# Creative experiences and brain clocks

**DOI:** 10.1038/s41467-025-64173-9

**Published:** 2025-10-03

**Authors:** Carlos Coronel-Oliveros, Joaquin Migeot, Fernando Lehue, Lucia Amoruso, Natalia Kowalczyk-Grębska, Natalia Jakubowska, Kanad N. Mandke, Joana Pereira Seabra, Patricio Orio, Dominic Campbell, Raul Gonzalez-Gomez, Pavel Prado, Jhosmary Cuadros, Enzo Tagliazucchi, Josephine Cruzat, Agustina Legaz, Vicente Medel, Hernan Hernandez, Sol Fittipaldi, Florencia Altschuler, Sebastian Moguilner, Sandra Baez, Hernando Santamaria-Garcia, Alfredis González-Hernández, Jasmin Bonilla-Santos, Bahar Güntekin, Claudio Babiloni, Daniel Abasolo, Gaetano Di Caterina, Görsev G. Yener, Javier Escudero, John Fredy Ochoa-Gómez, Marcio Soto-Añari, Martin A. Bruno, Pedro A. Valdes-Sosa, Renato Anghinah, Rodrigo A. Gonzalez-Montealegre, Ruaridh A. Clark, Adolfo M. García, Laura Kaltwasser, Martin Schürmann, Jil M. Meier, Aneta Brzezicka, Robert Whelan, Brian Lawlor, Ian H. Robertson, Christopher Bailey, Lucia Melloni, Nisha Sajnani, Agustin Ibanez

**Affiliations:** 1 Latin American Brain Health Institute (BrainLat), Universidad Adolfo Ibañez, Santiago de Chile, Santiaguinos, Chile; 2 Trinity College Dublin, The University of Dublin, Dublin, Ireland; 3 Global Brain Health Institute (GBHI), University of California, San Francisco, US and Trinity College Dublin, Dublin, Ireland; 4 Cognitive Neuroscience Center (CNC), Universidad de San Andrés, Buenos Aires, Argentina; 5 Basque Center on Cognition, Brain and Language (BCBL), San Sebastian, Spain; 6 Ikerbasque, Basque Foundation for Science, Bilbao, Spain; 7 Faculty of Psychology, SWPS University of Social Sciences and Humanities, Chodakowska 19/31, Warsaw, Poland; 8 Centre for Neuroscience in Education, Department of Psychology, University of Cambridge, Cambridge, United Kingdom; 9 Berlin School of Mind and Brain, Humboldt-Universität zu Berlin, Berlin, Germany; 10 Centro Interdisciplinario de Neurociencia de Valparaíso (CINV), Universidad de Valparaíso, Valparaíso, Chile; 11 Instituto de Neurociencia, Facultad de Ciencias, Universidad de Valparaíso, Valparaíso, Chile; 12 Escuela de Fonoaudiología, Facultad de Odontología y Ciencias de la Rehabilitación, Universidad San Sebastián, Santiago, Chile; 13 Advanced Center for Electrical and Electronic Engineering, Universidad Técnica Federico Santa María, Valparaíso, Chile; 14 Grupo de Bioingeniería, Decanato de Investigación, Universidad Nacional Experimental del Táchira, San Cristóbal, Venezuela; 15 Harvard Medical School, Boston, MA USA; 16 Universidad de los Andes, Bogotá, Colombia; 17 Pontificia Universidad Javeriana (PhD Program in Neuroscience) Bogotá, San Ignacio, Colombia; 18 Center of Memory and Cognition Intellectus, Hospital Universitario San Ignacio Bogotá, San Ignacio, Colombia; 19 Department of Psychology, Master programme of Clinical Neuropsychology, Universidad Surcolombiana, Neiva, Huila Colombia; 20 Department of Psychology, Universidad Cooperativa de Colombia, Neiva, Colombia; 21 Department of Neurosciences, Health Sciences Institute, Istanbul Medipol University, İstanbul, Turkey; 22 Health Sciences and Technology Research Institute (SABITA), Istanbul Medipol University, Istanbul, Turkey; 23 Department of Biophysics, School of Medicine, Istanbul Medipol University, Istanbul, Turkey; 24 Department of Physiology and Pharmacology ‘V. Erspamer’, Sapienza University of Rome, Rome, Italy; 25 Hospital San Raffaele Cassino, Cassino, Italy; 26 Centre for Biomedical Engineering, School of Mechanical Engineering Sciences, University of Surrey, Guildford, UK; 27 Department of Electronic and Electrical Engineering, University of Strathclyde, Glasgow, UK; 28 Faculty of Medicine, Izmir University of Economics, Izmir, Turkey; 29 Brain Dynamics Multidisciplinary Research Center, Dokuz Eylul University, Izmir, Turkey; 30 Izmir Biomedicine and Genome Center, Izmir, Turkey; 31 School of Engineering, Institute for Imaging, Data and Communications, University of Edinburgh, Edinburgh, UK; 32 Grupo de Neurociencias de Antioquia (GNA), University of Antioquia, Medellín, Colombia; 33 Universidad Católica San Pablo, Arequipa, Peru; 34 Instituto de Ciencias Biomédicas (ICBM), Universidad Católica de Cuyo, San Juan, Argentina; 35 Consejo Nacional de Investigaciones Científicas y Técnicas (CONICET), Buenos Aires, Argentina; 36 The Clinical Hospital of Chengdu Brain Sciences Institute, University of Electronic Sciences and Technology of China, Chengdu, China; 37 Cuban Neuroscience Center, La Habana, Cuba; 38 Reference Center of Behavioural Disturbances and Dementia, School of Medicine, University of Sao Paulo, Sao Paulo, Brazil; 39 Traumatic Brain Injury Cognitive Rehabilitation Out-Patient Center, University of Sao Paulo, Sao Paulo, Brazil; 40 Neurocognition and Psychophysiology Laboratory, Universidad Surcolombiana, Neiva, Colombia; 41 Centre for Signal and Image Processing, Department of Electronic and Electrical Engineering, University of Strathclyde, Strathclyde, UK; 42 Departamento de Lingüística y Literatura, Facultad de Humanidades, Universidad de Santiago de Chile, Santiago, Chile; 43 School of Psychology, University of Nottingham, Nottingham, United Kingdom; 44 Berlin Institute of Health, Charité – Universitätsmedizin Berlin, Berlin, Germany; 45 Department of Neurology with Experimental Neurology, Brain Simulation Section, Charité – Universitätsmedizin Berlin, Berlin, Germany; 46 World Health Organization, Geneva, Switzerland; 47 Department of Neurology, New York University Grossman School of Medicine, New York, USA; 48 Neural Circuits, Consciousness, and Cognition Research Group, Max Planck Institute for Empirical Aesthetics, Frankfurt am Main, Germany; 49 Predictive Brain Department, Faculty of Psychology, Ruhr University Bochum, Bochum, Germany; 50 Jameel Arts & Health Lab, New York University, New York, USA

**Keywords:** Cognitive neuroscience, Human behaviour, Predictive markers

## Abstract

Creative experiences may enhance brain health, yet metrics and mechanisms remain elusive. We characterized brain health using brain clocks, which capture deviations from chronological age (i.e., accelerated or delayed brain aging). We combined M/EEG functional connectivity (*N* = 1,240) with machine learning support vector machines, whole-brain modeling, and *Neurosynth* metanalyses. From this framework, we reanalyzed previously published datasets of expert and matched non-expert participants in dance, music, visual arts, and video games, along with a pre/post-learning study (*N* = 232). We found delayed brain age across all domains and scalable effects (expertise>learning). The higher the level of expertise and performance, the greater the delay in brain age. Age-vulnerable brain hubs showed increased connectivity linked to creativity, particularly in areas related to expertise and creative experiences. *Neurosynth* analysis and computational modeling revealed plasticity-driven increases in brain efficiency and biophysical coupling, in creativity-specific delayed brain aging. Findings indicate a domain‑independent link between creativity and brain health.

## Abstract (web summary)

Creative experiences such as dance, music, drawing, and strategy video games might preserve brain health. The authors show that regular practice or short training in these activities is linked to brains that look younger and work more efficiently.

## Introduction

Creative and artistic experiences have recently been proposed to improve brain health^1,2^. Creativity is defined here as the ability to produce ideas or solutions that are both novel and effective using one’s imagination. Creativity traditionally includes the arts, but video games can incorporate creative elements^3^. For instance, strategy video games, such as StarCraft II, materialize creativity in unique tactics, adaptive problem-solving, and personalized playstyles. These experiences, which include multiple domains from the arts to gaming^1,2,4^, may foster well-being in healthy participants and those with psychological and neurological disorders. Increased brain volume and connectivity correlate with different creative experiences^4,5^. However, the available evidence on specific brain health benefits is scarce^6^. Most studies comprise cognitive and emotional effects and well-being or neural correlates, without providing evidence for protective effects on the brain^6^.

Brain clocks measure accelerated brain age as observed in multiple brain diseases^7–10^. They offer a robust approach to assess accelerated or delayed brain aging by measuring deviations between chronological age and brain age, commonly referred to as brain age gaps (BAGs)^8–10^. These BAGs, derived from neuroimaging data, provide a quantitative measure of brain health, identifying individuals with faster or slower aging processes. Accelerated aging (larger positive BAGs) is observed in psychiatric and neurological conditions^8–10^, in vulnerable populations exposed to physical and social exposomes^9,11–13^, or in those with unhealthy lifestyles^9,13^. Conversely, people with healthier habits, such as systematic creative experiences, could be hypothesized to have delayed aging. Only a few works addressed the impact of creativity, specifically music expertise, on brain structural correlates and reported contradictory results^14,15^. While structural and functional BAGs can be estimated, the latter may be more sensitive to plasticity-driven effects of interventions and lifestyle on brain aging^9,16^.

A critical concern in the literature on the health effects of creative experiences is the lack of mechanistic insight^17^. Biophysical models and graph theory can help elucidate general mechanisms of aging^7,18^, such as the inter-area coupling modulated by plasticity and the underlying organization of brain networks^19,20^. These mechanisms and organizational features, as revealed through generative models and graph-theoretical approaches^7^, could inform the properties of brain clocks and their potential modulation by creative experiences. This synergy can help to understand the underlying brain mechanisms driving accelerated or delayed brain aging^7^. Thus, brain clocks combined with mechanistic models represent a promising tool for investigating the potential protective effects of diverse creative experiences on brain health. To our knowledge, no studies have yet explored this relationship.

Here, we investigate the potential protective effects of creative experiences on brain clocks in a large sample of participants (N = 1472). We developed creative-sensitive measures of accelerated and delayed brain age using brain clocks, graph theory, and biophysical modeling^7–9,21^. First, we robustly estimated brain clock models using machine learning and EEG data from 1240 participants. Then, we computed brain age gaps from M/EEG data in individuals with different levels of creative experiences (*N* = 232). These involved age-, sex-, education- and geography-matched groups of expert and non-expert tango dancers, musicians, visual artists, and video game players (study 1); and explored the pre/post-effects of short-term learning in a separate group of video game training (study 2). This design allowed us to investigate and compare the effects of consolidated professional expertise vs short-term learning.

Here, we showed that cross-domain creative experiences are associated with delayed brain aging as measured by BAGs. We found that lower negative BAGs in people with more creative experiences would be mainly observed in frontoparietal hubs, as these are regions vulnerable to aging^22–25^. An association between delayed brain aging and the degree of creative expertise was observed in experts (e.g., years of practice) and learners (pre/post-learning outcomes)^26–28^. These effects were larger in the expertise study that involved long-term training than pre/post-effects in non-expert participants involving short-term learning^28–30^. Using biophysical modeling and graph theory, we observed that creative experiences are related to plasticity-driven functional alterations^27,31^, such as increased local and global efficiency^26,27^ and modulations in biophysical coupling^32,33^, as previous reports show impaired segregation and reduced connectivity strength in aging^34–36^. The design allows us to investigate cross-domain convergent impacts of creativity on BAGs, with specific mechanisms related to these potential protective effects, providing insights about the impact of cumulative creative experiences on brain health. These results may inform future public policies to improve health and well-being through creativity and the arts^37^.

## Results

**Fig. 1:**
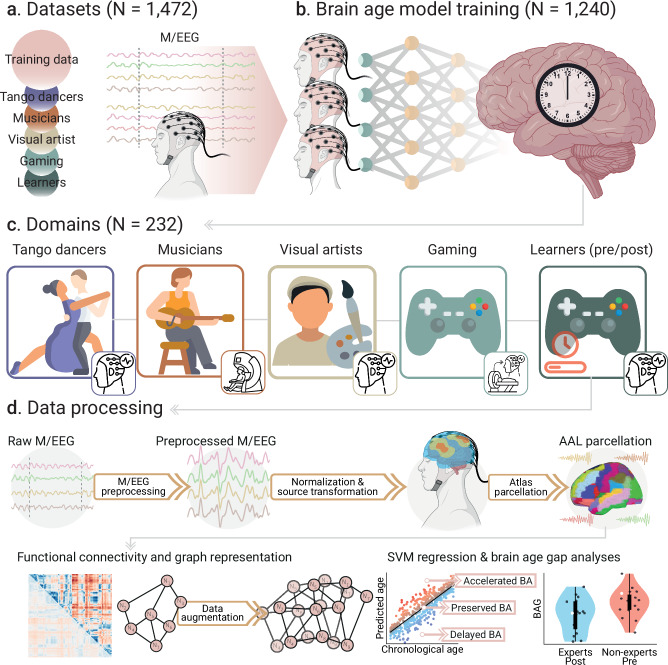
Data characterization, preprocessing pipeline, and data analysis. **a** We included M/EEG data from diverse populations (*N* = 1472) across 13 countries: Canada, Chile, Argentina, Cuba, Colombia, Brazil, the United Kingdom, Ireland, Italy, Greece, Turkey, Poland, and Germany. **b** We used a subsample of *N* = 1240 participants for training the support vector machines (SVMs) using EEG. These SVMs were used to predict the participants’ brain ages across all domains. **c** The remaining data (*N* = 232) was used for out-of-sample validation, and it consisted of M/EEG datasets related to different types of creative expertise and learning. Four of these groups represent creative expertise in dance (tango), music (instrumentalists and singers), visual arts (drawing), and video games (StarCraft 2) (study 1 about expertise). Additionally, we included one group with video game learning (StarCraft 2) (study 2 about pre/post-learning). **d** Before training the SVMs, raw M/EEG signals were preprocessed, normalized, and transformed into the source space using the AAL brain parcellation. From the source-transformed signals, we computed the functional connectivity matrices for all participants. We used data augmentation when training the SVMs to increase the model’s robustness and accuracy. From the trained SVMs, we computed the brain age gaps (BAGs) of participants as the difference between the model predictions and their chronological ages. BAGs > 0 can be interpreted as accelerated brain aging, and BAGs <0 as delayed brain aging. Points and violin plots in the figure are schematic examples. Created in BioRender. Migeot, J. (2025) https://BioRender.com/99vpcts (EEG device and brain illustrations).

The overall pipeline is shown in Fig. 1. We included 1,473 participants to (a) create brain clock models and (b) test them in groups with varying levels of creative experience (Fig. 1a, b). This analysis included two main groups: expert vs. non-expert participants across various creative domains (study 1), and non-experts undergoing short-term video game training (pre/post-learning, study 2) (Fig. 1c and Table 1). For the study of expertise, we included sex-, education-, and geography-matched groups of experts vs. non-expert participants in dance (tango, age range 18–50 years), music (instrumentalists and singers, 22–41 years), visual arts (drawing, age range 20–37 years), and real-time-strategy video games (StarCraft 2, age range 18–31 years). In the pre/post-learning study, we compared outcomes before and after video game training in non-expert participants (age range 20–30 years). The brain clock model was built using machine learning and source-localized EEG functional connectivity across 1,240 participants (age range 17-91 years) (Fig. 1d and Table 1). The architecture consisted of support vector machine (SVMs) models, trained with EEG connectivity using a 5-fold cross-validation and hyperparameter tuning. From the model, we computed BAGs, defined as the difference between predicted and chronological age. BAGs > 0 indicate accelerated brain aging, and BAGs < 0 indicate delayed aging (Fig. 1d). BAGs and brain clocks constitute a normative approach for systematically measuring the impact of creative experiences on brain health using the same space of M/EEG sources, defined by an anatomical brain parcellation^38^, and using a standardized score of brain health (the BAGs). The trained brain clocks were used to test the BAGs on 232 participants across and within creative domains.

**Table 1:** Demographic data

Group	Subgroup	Countries	Sample size	Age (years)	Education (years)	Sex*	Expertise criteria	Expertise level
Training data	Global south	Argentina,	37	46.0 (21.0)	13.8 (4.4)	603:593**	Not apply	Not apply
		Brazil	84					
		Chile	41					
		Colombia	134					
		Cuba	220					
	Global north	Greece	22					
		Ireland	132					
		Italy	20					
		Turkey	484					
		UK	66					
		Total	1240					
Tango dancers	Experts	Argentina	23	31.3 (5.0)	18.3 (2.6)	18:5	Formal tango instruction (months)	56.8 (46.2)
	Non-experts		23	29.2 (5.9)	17.9 (2.8)	14:9		1.7 (3.1)
	Total		46	t(44) = 1.319, [−1.15,5.35]_95%_, *p* = 0.193	t(44) = 0.478, [−1.21, 2.01]_95%_, *p* = 0.635	X^2^(1) = 0.924, *p* = 0.336		t(44) = 5.581, [35.09, 75.11]_95%_, *p* < 0.001
Musicians	Experts	Canada	29	28.6 (7.5)	17.2 (3.3)	18:11	Musical experience (years)	13.3 (5.1)
	Non-experts		29	27.4 (6.0)	15.7 (3.6)	19:10		0.0 (0.0)
	Total		58	t(56) = 0.667, [−2.38, 4.78]_95%_, *p* = 0.508	t(56) = 0.592, [-0.32, 3.32]_95%_, *p* = 0.565	X^2^(1) = 0.0, *p* = 1.0		t(56) = 13.853, [11.36, 15.24]_95%_, *p* < 0.001
Visual artists	Experts	Germany	15	28.0 (5.3)		12:3	University-level art education (years)	4.5 (1.5)
	Non-experts		15	27.9 (4.3)		11:4		0.0 (0.0)
	Total		30	t(28) = 0.074, [-3.52, 3.72]_95%_, *p* = 0.942		X^2^(1) = 0.0, *p* = 1.0		t(28) = 11.439, [3.67, 5.33]_95%_, *p* < 0.001
Gaming	Experts	Poland	31	24.7 (4.2)	15.6 (2.8)	All male	StarCraft II experience (hours per week)	18.2 (10.0)
	Non-experts		31	24.4 (3.0)	16.1 (3.0)	All male		0.0 (0.0)
	Total		62	t(60) = 0.320, [−1.56, 2.16]_95%_, *p* = 0.750	t(60) = -0.757, [−1.97, 0.97]_95%_, p = 0.452	X^2^(1) = 0.0, *p* = 1.0		t(60) = 10.140, [14.53, 21.87]_95%_, *p* < 0.001
Pre/post-learning	Intervention	Poland	24	24.8 (3.4)	15.6 (2.5)	12:12	Unexperienced	Not apply
	Active control		12	25.1 (4.5)	16.6 (2.5)	3:9		Not apply
	Total		36					

For age, education, and expertise level, the data reported consisted of the mean and standard deviation in parentheses; 95% confidence intervals in square brackets. Differences in age, education, and expertise were assessed using Student *t*-tests. For sex, we used Chi-squared tests. Degrees of freedom in parentheses. Global south countries: Argentina, Chile, Brazil, Cuba, and Colombia. Global North countries: Ireland, Italy, the UK, Greece, and Turkey. *Sex as female vs male ratio. **Missing values about sex exclude.

**Fig. 2:**
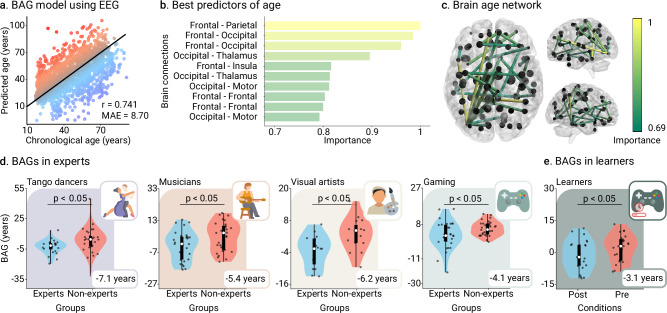
Brain clock model and brain age gaps (BAGs). **a** The model’s performance was assessed by computing the Pearson’s correlation and the mean absolute error (MAE) between the predicted age and the real chronological age of participants (*N* = 1,240 participants). Red/blue colors represent accelerated/delayed aging. **b** Most important (informative) brain connections for predicting age. Top connections reflect the highest absolute SVR weights, indicating their importance in age prediction. **c** The brain age network comprises the set of most informative connections; the thickness of the edges represents the features’ importance of connections for predicting age using SVMs. **d** BAGs in the expertise study (*N* = 196 participants), i.e., tango dancers (ΔBAGs = −5.50, [−8.17, −2.84]_95%_, t(194) = −4.823, *p* < 0.001, D = −0.69), musicians (ΔBAGs = −5.38, [−10.21, −0.56]_95%_, t(58) = −2.237, *p* = 0.035, D = −0.60), visual artists (ΔBAGs = −6.2, [−10.79, −1.60]_95%_, t(28) = −2.761, *p* = 0.028, D = −1.04), and gaming (ΔBAGs = −5.38, [−10.21, −0.56]_95%_, t(58) = −2.237, *p* = 0.035, D = −0.60). **e**. BAGs in the pre/post-learning study (*N* = 24 participants) (ΔBAGs = −3.06, [−5.27, −0.85]_95%_, t(23) = −2.863, *p* = 0.028, *D* = −0.46, FDR-corrected). Points in scatter plots represent participants. Box plots show the median and the first and third quartiles; whiskers mark the minimum and maximum values, and each point represents one participant. Groups were compared using t-statistics with two-sided p-values, FDR-corrected.

The training brain clock model yielded robust results. Accurate age estimation performance was consistent with previous studies using the mean absolute error (MAE) and Pearson’s correlation^8,39^ (MAE = 8.696 years, r = 0.742, *p* < 0.001, *N* = 1240 participants, Cohen’s f ^2^ = 1.11, Fig. 2a). BAGs were associated with reduced connectivity in age-related networks, specifically frontoparietal and frontal-to-occipital connections^22–25^ (Fig. 2b, c and Fig. S2 in Supplementary Information). BAGs were not explained by differences in data quality (Fig. S1 in Supplementary Information).

### Higher levels of creative experiences are associated with delayed brain age

Lower negative BAGs were observed in experts than in non-experts across domains (ΔBAGs = −5.50, [−8.17, −2.84]_95%_, t(194) = −4.823, *p* < 0.001, *D* = −0.69, Fig. 2). These results were replicated in each domain: tango dancers (ΔBAGs = -7.1, [−12.82, −1.47]_95%_, t(44) = −2.563, *p* = 0.028, *D* = -0.77, Fig. 2d) musicians (ΔBAGs = −5.38, [−10.21, −0.56]_95%_, t(58) = −2.237, *p* = 0.035, *D* = −0.60, Fig. 2d), visual arts (ΔBAGs = −6.2, [−10.79, −1.60]_95%_, t(28) = −2.761, *p* = 0.028, *D* = −1.04, Fig. 2d), and gaming (ΔBAGs = −4.06, [−7.43, −0.68]_95%_, t(60) = −2.422, *p* = 0.028, *D* = −0.63, Fig. 2d). In the pre/post-learning study, lower negative BAGs were observed in the post-learning condition compared to the pre-learning one (ΔBAGs = −3.06, [−5.27, −0.85]_95%_, t(23) = −2.863, *p* = 0.028, *D* = −0.46, FDR-corrected, Fig. 2e). These effects were specific to StarCraft II itself, as we did not find any differences in the active control of the pre/post design (ΔBAGs = 0.057, [−4.51, 4.62]_95%_, t(11) = 0.0274, *p* = 0.979, *D* = 0.0092, FDR-corrected, Figure S11 in Supplementary Information). Conversely, significant differences were observed when comparing learners and active controls (Δ(ΔBAGs) = −3.152, [−8.04, 1.81]_95%_, U(35) = 90, *p* = 0.036, *D* = −0.49, Table S11 in Supplementary Information). Thus, results showed moderate to large effects of creativity expertise on BAGs, and small to moderate effects of short-term learning.

### Degree of expertise and performance modulate the brain age gaps

**Fig. 3:**
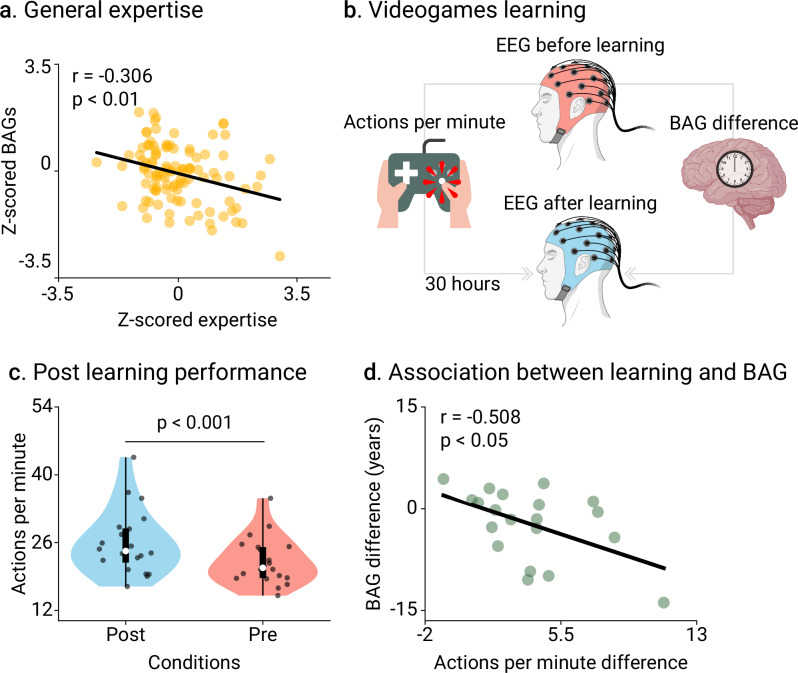
Creative experiences relationship with brain age gaps (BAGs). **a** Correlation between BAGs and scores of expertise for tango dancers, musicians, visual artists, and gaming (*r* = −0.306, *p* = 0.003, *N* = 105 participants, Cohen’s f ^2^ = 0.103). Scores and BAGs were previously transformed into z-scores. **b** Scheme representation of the pre/post-learning study design. EEG recordings were acquired before learning (period 0) and thereafter (period 2). The in-game performance was assessed using the average actions per minute (APM) between periods 2 and 1 (post), and periods 0 and 1 (pre). **c**, **d**. Post- and pre-learning APM (*N* = 20 participants) (ΔAPM = 3.83, [2.45, 5.21]_95%_, t(19) = 5.804, *p* < 0.001, *D* = 0.68), and correlation between changes in APM and BAGs (*r* = -0.508, *p* = 0.022, *N* = 20 participants, Cohen’s f ^2^ = 0.349). Points represent participants. Box plots show the median and the first and third quartiles; whiskers mark the minimum and maximum values, and each point represents one participant. Groups were compared using t-statistics with two-sided p-values, FDR-corrected. Created in BioRender. Migeot, J. (2025) https://BioRender.com/99vpcts (EEG device and brain illustrations).

We explored the associations of levels of creative experience with BAGs. In the expertise study, the BAGs and the expertise scores were converted into z-scores (section Expertise scores and BAGs standardization in Methods for details). The more skilled participants have lower BAGs (*r* = −0.306, *p* = 0.003, N = 105 participants, Cohen’s f ^2^ = 0.103, FDR-corrected, Fig. 3a). Thus, the degree of individual expertise across four domains (tango dancers, musicians, visual artists, and gaming) was associated with delayed brain age. For the pre/post-learning study, we used the number of actions per minute (APM, section Pre/post-learning design in Methods), which provides a reliable score of in-game performance^40^, increased after post-learning (ΔAPM = 3.83, [2.45, 5.21]_95%_, t(19) = 5.804, *p* < 0.001, *D* = 0.68, Fig. 3b-c). A negative correlation between ΔBAGs and ΔAPM (*r* = −0.508, *p* = 0.022, *N* = 20 participants, Cohen’s f ^2^ = 0.349, FDR-corrected, Fig. 3d) evidenced that participants with reduced post-learning BAGs also showed improved performance.

**Fig. 4:**
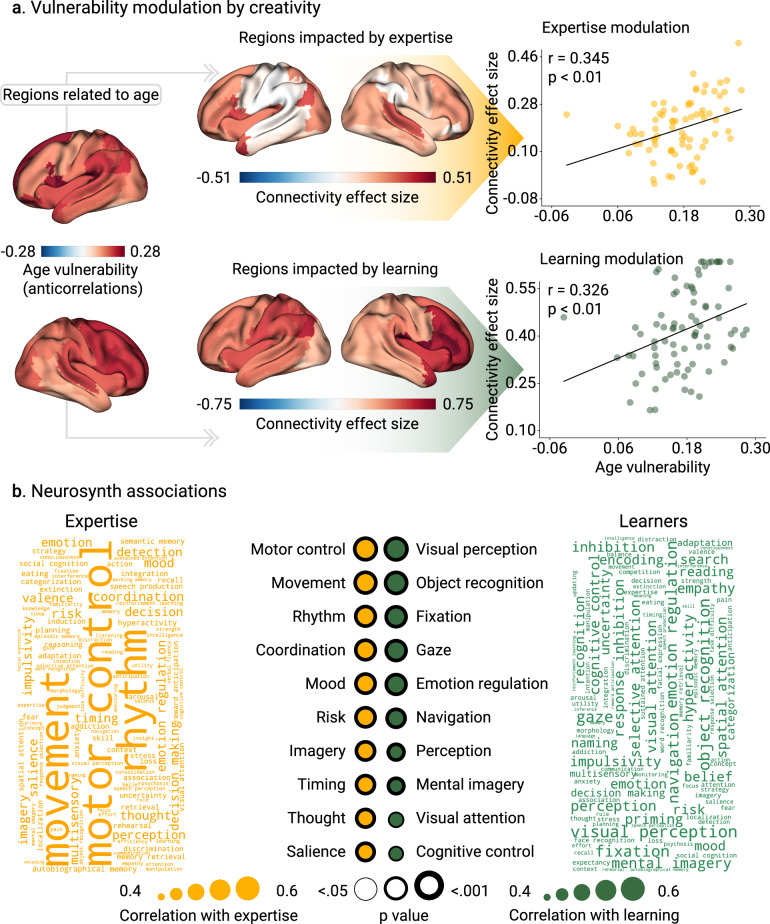
Topographic patterns of connectivity associated with creative experiences. **a** The anticorrelation between nodal functional connectivity and age represents age vulnerability. Associations between age vulnerability and increased brain connectivity are driven by creative experiences. The areas that have the greatest increase in connectivity are the ones with higher Cohen’s D effect sizes. The brain of the experts’ group is the average brain across dance dancers, musicians, visual artists, and gamers. Scatter plots represent the associations in expertise (*r* = 0.345, *p* < 0.001, *N* = 78 brain areas, Cohen’s f ^2^ = 0.135) and learning (*r* = 0.326, *p* < 0.001, *N* = 78 brain areas, Cohen’s f ^2^ = 0.119). Points in scatter plots represent brain areas. **b** *Neurosynth* associations with brain connectivity increase in creative experiences. We reported the absolute Pearson’s correlation between brain connectivity and association maps of different cognitive processes (*N* = 78 brain areas). FDR-corrected p-values are shown, and the thickness of the circles represents statistical significance. The p-values were computed using the Spin test up to 10,000 permutations before performing the FDR correction.

Using the attentional blink paradigm task outcomes, we then analyzed how the effects of pre/post learning can be translated to generalized performance. Results showed significant improvements after training: participants responded faster to the first question (T1; Δ = −141.28, [−238.84, −43.73]_95%_, t(23) = −2.996, *p* = 0.026, D = −0.61, FDR-corrected), and exhibited higher accuracy on the second question (T2; Δ = 6.71, [−10.75, 24.16]_95%_, t(23) = 5.958, *p* < 0.001, D = 1.22, FDR-corrected). We found no effects in the active control group (results in Supplementary Information Table S6).

### The BAG networks comprise regions with age-related vulnerability and expertise

We analyzed whether creative experience had a protective effect in age-vulnerable brain regions. Using the training data, we built a map of brain age vulnerability, which consisted of the areas anticorrelated with chronological age (section Brain maps and *Neurosynth* associations in Methods for details). Then, we explored the association of regional alterations in nodal strength in (a) expert vs non-expert participants, and (b) pre/post-learning (Fig. 4a). In the expertise study, a correlation between age-related vulnerability regions and changes in nodal strength (*r* = 0.345, *p* < 0.001, *N* = 78 brain areas, Cohen’s f ^2^ = 0.135, spin test, FDR-corrected, Fig. 4a) involved frontoparietal hubs. In the pre/post-learning study, we also found similar significant associations (*r* = 0.326, *p* < 0.001, *N* = 78 brain areas, Cohen’s f ^2^ = 0.119, FDR-corrected, Fig. 4a).

We performed a *Neurosynth*^21^ analysis to explore the possible underlying cognitive processes involved in the BAG association, i.e., increased connectivity strength with creativity-related cognitive process (Fig. 4b). In the expertise study, the main regions were involved in motor control, movement, rhythm, coordination, imaginary, and visual salience, among others (Fig. 4b). In the pre/post-learning study, associations with cognitive domains were primarily related to attention, i.e., visual perception, object recognition, fixation, perception, and visual attention (Fig. 4b).

### Increased network efficiency and coupling strength in creative experiences

**Fig. 5:**
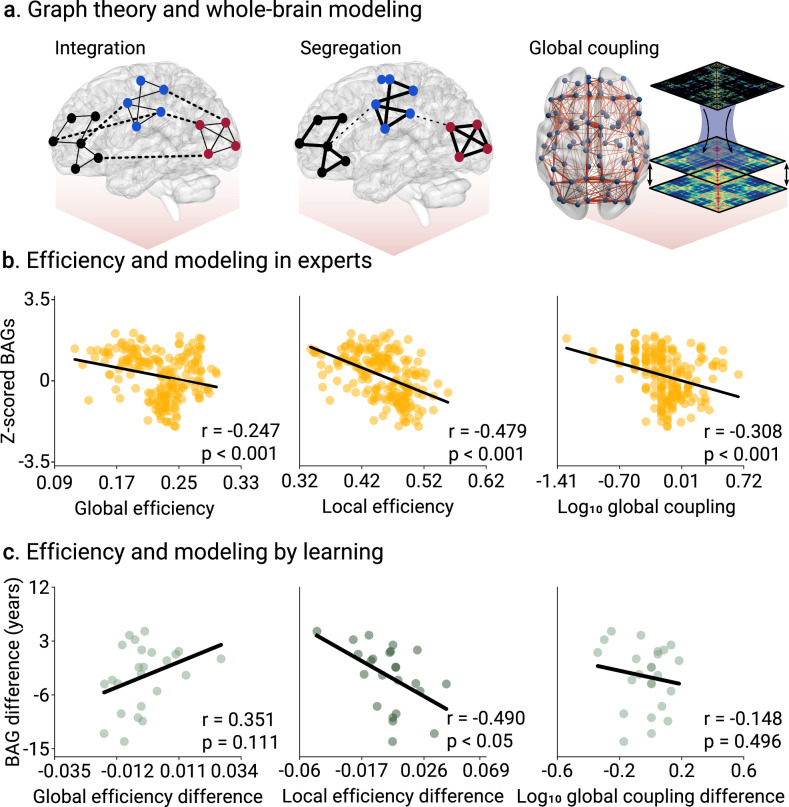
General organizational and mechanistic principles associated with brain age gaps (BAGs) in creative experiences. **a** Using M/EEG functional connectivity, we characterized two efficiency-based properties of brain topology, namely integration, related to general information processing, and segregation, ascribed to specialized information processing. We then used a generative model of EEG activity to test mechanisms based on global coupling modulation. **b** Efficiency metrics and modeling parameters in the expertise design (tango dancers, musicians, visual artists, and gaming). We reported significant correlations between BAGs and global efficiency (*r* = −0.247, *p* < 0.001, *N* = 195 participants, Cohen’s f ^2^ = 0.065), local efficiency (*r* = −0.479, *p* < 0.001, *N* = 195 participants, Cohen’s f ^2^ = 0.298), and global coupling (*r* = −0.351, *p* < 0.001, *N* = 195 participants, Cohen’s f ^2^ = 0.105). **c** Efficiency metrics and modeling parameters in the pre/post-learning design. We reported significant correlations between BAGs and local efficiency (*r* = −0.490, *p* = 0.023, *N* = 24 participants, Cohen’s f ^2^ = 0.316), but not with either global efficiency (*r* = 0.351, *p* = 0.111, *N* = 24 participants, Cohen’s f ^2^ = 0.141), or global coupling (*r* = −0.148, *p* = 0.492, *N* = 24 participants, Cohen’s f ^2^ = 0.022). Points in scatter plots represent participants. FDR-corrected p-values.

We explored causal mechanisms associated with BAGs. First, we investigated the BAG networks and brain efficiency via graph theory^41^, using global and local efficiency measures (Fig. 5a). In the expertise study, a small negative correlation between z-scored BAGs and global efficiency (*r* = −0.247, *p* < 0.001, *N* = 195 participants, Cohen’s f ^2^ = 0.065, FDR-corrected, Fig. 5b), and a large correlation with local efficiency (*r* = −0.479, *p* < 0.001, *N* = 195 participants, Cohen’s f ^2^ = 0.298, FDR-corrected, Fig. 5b) were observed. In the pre/post-learning study, the same association was identified but only with Δlocal efficiency (*r* = −0.490, *p* = 0.023, *N* = 24 participants, Cohen’s f ^2^ = 0.316, FDR-corrected, Fig. 5c). Thus, the lower the BAGs, the more efficient the brain networks are, especially at local levels.

Second, generative whole-brain modeling was used to investigate causal mechanisms based on generative modeling. The global coupling parameter modulates the biophysical strength of brain connections^32,33^ (Fig. 5a, see also the goodness of fit in Figs. S4, 5 in Supplementary Information). In the expertise study, a negative correlation between BAGs and global coupling (r = −0.351, *p* < 0.001, *N* = 195 participants, Cohen’s f ^2^ = 0.105, FDR-corrected, Fig. 5b) suggests that long-term creative expertise drives biophysical coupling across domains. As expected, this effect was not observed in the more transient and short-term creative experiences assessed in the pre/post-study (*r* = −0.148, *p* = 0.492, *N* = 24 participants, Cohen’s f ^2^ = 0.022, FDR-corrected, Fig. 5b)

### Sensitivity analyses

ANCOVAs controlling for age, sex, and education confirmed that expertise significantly predicted BAGs across all creative domains (tango dancers, musicians, visual artists, and gamers). These effects remained significant after FDR correction, supporting the robustness of our main findings (Table S8 in Supplementary Information).

We found that the spatial correlations between age vulnerability and connectivity effect sizes remained significant when analyzed separately for each domain. Specifically, robust positive correlations were observed for tango dancers (*r* = 0.333, *p* = 0.002, *N* = 78 brain areas, f² = 0.125, spin test, FDR-corrected), musicians (*r* = 0.350, *p* = 0.001, *N* = 78 brain areas, f² = 0.140, spin test, FDR-corrected), visual artists (*r* = 0.246, *p* = 0.013, *N* = 78 brain areas, f² = 0.064, spin test, FDR-corrected), and gaming (*r* = 0.282, *p* = 0.01, *N* = 78 brain areas, f² = 0.086, spin test, FDR-corrected) (Fig. S8 in Supplementary Information).

Participants classified as BAG-younger (i.e., with brain age gaps below the 35th percentile) showed significantly higher domain-specific expertise than those classified as BAG-older (i.e., above the 65th percentile). This pattern was consistent across domains: tango dancers (Δ = 21.25, [−13.26, 55.76]_95%_, U(30) = 118.5, *p* = 0.016, FDR-corrected), musicians (Δ = 4.75, [−0.22, 9.72,]_95%_, U(38) = 26.5, *p* = 0.030, FDR-corrected), visual artists (Δ = 2.77, [−2.09, 4.85]_95%_, U(20) = 138, *p* = 0.008, FDR-corrected *p* = 0.016), and gaming (Δ = 11.21, [4.47, 17.95]_95%_, U(42) = 69.5, *p* = 0.005, FDR-corrected). The results are summarized in Table S9 in the Supplementary Information.

We compared BAG estimates with and without age bias correction. The main group effects remained consistent across approaches (Fig. S10 in Supplementary Information), confirming that our findings are not driven by regression-to-the-mean artifacts. We observed no meaningful differences in prediction slopes between 64- and 128-channel EEG data in either the training or creativity datasets (Supplementary Table S10).

## Discussion

This study addressed how different creative experiences were associated with brain health measures of delayed biological age while exploring age-related brain mechanisms. Creative experiences were linked to delayed brain aging across domains and in both expertise and short-term learning studies. The degree of expertise and learning correlated with BAGs. Creative experiences enhanced connectivity in brain regions vulnerable to age, as well as in areas associated with creativity processes. Increased network efficiency and global coupling contributed to lower BAGs, which were more accentuated in the expertise study. Thus, delayed brain aging, driven by creative experiences, presented an inverted pattern (accelerated aging) seen in many neurological disorders^8–11^. Our work provides evidence of reduced accelerated aging in domain-free creativity linked to expertise, experience, and underlying brain plasticity mechanisms.

Most of the evidence on brain clocks suggests that adverse conditions, such as neurological diseases, induce accelerated aging^8,9,11,18,22^. However, positive social determinants of brain health^13^ and lifestyles, such as physical activity and good cardiometabolic health^9,13,42^, education^13,43,44^, and prosociality^45,46^ may reduce accelerated aging. Organ and brain clocks have shown how protective factors modulate brain aging, reducing BAGs^8–10^. Although earlier studies suggested a potential effect of creativity on brain health^1,2^, our work directly addressed this question by showing delayed brain age in multiple creative domains. Domain-free results suggest that different creative experiences share common mechanisms^31^ and impact networks associated with aging^22–25^. Present results support the potential of creativity-based interventions for preventive strategies and supportive therapies for healthy populations and clinical settings^1–4,37^.

Delayed brain aging due to creative experiences could be attributed to neural plasticity mechanisms^31^ and brain specialization^5,27^. Increased local efficiency through creativity is a hallmark of brain specialization, enhancing the communication bandwidth of expertise-related networks^26–28^, while higher global coupling improved communication across the brain^19^. Many studies have assessed graph properties associated with aging^34–36^, but no previous study has used them to describe the organizational properties of BAGs. Our results suggest that increased local efficiency with lower BAGs is similar to other brain organizational changes observed with aging, i.e., the reduction of brain segregation^34–36^. Neural plasticity mechanisms^31^ can drive these changes, supporting more efficient brain connectivity and information transfer between networks^26,27,29,47–49^, with increased network efficiency directly contributing to lower BAGs. Creativity experiences have been linked to structural and functional changes in brain regions vulnerable to aging and involved in creativity-related cognitive processes^22–25^. These consisted mainly of frontoparietal hubs, critical for attention, motor control, coordination, and rhythm^5,25^. Longer training periods result in stronger neural plasticity effects^26,27,47^, which explains the greater impact on brain connectivity in experts compared to learners, and the correlation between BAGs and expertise level. More domain-specific and smaller effects observed in the learning study might be ascribed to the short-term nature of the training design and specific video game engagement^5^.

The present work has multiple strengths. We utilized a large and diverse sample for both training the brain clocks, and for different creative experiences, applying state-of-the-art computational methods (machine learning, Neurosynth metanalysis, graph theory, and generative modeling) to assess the effect of creativity on brain health. Our analytical pipeline effectively addressed key confounding factors related to recordings, signals, and demographics, ensuring the reliability and accuracy of the BAGs computation. Although previous works found mixed effects of music expertise on brain health using structural brain age estimation^14,15^, our work made a step forward using functional connectivity and showing a domain-free positive effect of creativity on brain health. We found a tiered effect of creativity, with higher effects associated with long-term expertise and lower effects in short-term learning; all these outcomes scaled with participants’ skills and post-training performance. The inclusion of an active control group allowed us to isolate the specific effects of learning, as we observed no BAGs or cognitive changes associated with active controls. This reflects the genuine training-related plasticity effects in our pre/post-learning design, showing that short, targeted training can improve specific and generalized performance. Lastly, we provided plasticity-related mechanisms underlying creativity’s differential effects on brain clocks.

Our study also presents multiple limitations, calling for further research. We focused on brain aging, to understand the biological embedding and health-related measures of creativity. Future research should combine brain clocks with cognition, physical health, and well-being^9,10,13^. Accelerated aging, as measured with increased positive BAGs in literature, is typically associated with adverse outcomes, including disease, unhealthy lifestyles, or increased disparities^8,9^. However, future studies should develop methodologies that disentangle the potential positive healthy aging outcomes. Although our sample was heterogeneous, we used a normative approach that produced consistent results across groups. While the sample size of each group was relatively small, it remains one of the largest samples of studies in creativity and neuroscience, showing systematic effects in each group and across domains. Still, larger studies exploring additional creative domains are needed, e.g., acting and writing. Gaming can be considered a type of creative experience^3^, even though it is not traditionally classified as such. For this reason, we repeated the analyses in the expertise study, excluding the gaming groups. The results remained unchanged (Fig. S7 in Supplementary Information). Despite the heterogeneity of the groups, the findings consistently suggest delayed brain aging across all domains. Moreover, we observed domain-specific associations between artistic performance and brain clock metrics. If other than creativity effects (i.e., cognitively engaging activities) underlie these effects, they likely constitute core components of creative experience that manifest across modalities. Future studies should investigate the underlying subprocesses of creative experiences and their potential distinct effects on brain clocks. An additional limitation of our work is the small sample size used for the pre/post-learning design, and especially the active control group. Although both results convergently showed the effects of the intervention, the limited sample size could under/overestimate the magnitude of the outcomes. While EEG simulations derived from structural data (DTI) offer valuable insights into large-scale brain dynamics^27^, they remain approximations of actual neural activity and should, therefore, be interpreted with caution. However, EEG simulations and other datasets showed similar BAG effects. From a modeling point of view, additional neural plasticity mechanisms should be further assessed with other architectures, such as neural mass models^19,32,33,50^, and by integrating M/EEG and fMRI to provide cross-modal mechanisms.

We used Pearson’s correlation for its interpretability and reproducibility across different modalities. Source localization reduces, but does not eliminate, volume conduction and spatial leakage, improving the reliability of correlation-based connectivity relative to sensor-level analyses^51^. However, as Pearson’s correlation is susceptible to volume conduction artifacts^52^, future works should be replicated using alternative connectivity metrics^53^ (e.g., imaginary coherence, phase-lag index, or high-order interactions) to ensure the robustness of findings. Another limitation was the use of EEG data recorded with different numbers of electrodes, as lower sensor density may increase signal leakage and distort connectivity estimates^54^. However, our sensitivity analyses showed that electrode count did not affect the brain age models. Compared to raw functional connectivity measures, BAGs provide coarser-grained estimates that are more robust to sensor density variations. In previous work^8^, BAG estimates remained stable across EEG systems, channel counts, and signal quality when our data harmonization was applied. Importantly, each dataset in the creativity study included its own control group, ensuring that differences between experts and non-experts or pre- and post-learning were not confounded by electrode numbers.

As the sample used to train the data likely includes more non-experts than experts, brain age models may present a bias. However, there are multiple reasons to believe this is not the case. The experimental design controls for typical variables (age, sex, education, and in some domains, additional factors such as general cognition). Differences between BAGs—at least in terms of the variables typically used for group comparisons—are better explained by differences in creativity. Moreover, the consistency of delayed brain age was observed across groups, domains, and comparisons (between-group contrasts and pre/post designs), suggesting a systematic association between creativity and delayed brain age, despite potential random factors. Furthermore, the association and consistent directionality of BAGs with performance measures, along with the same effects in pre/post design, reinforce this idea. Nonetheless, we cannot fully rule out other contributing factors, and future research should address potential additional confounders. Demographic and individual factors are known to influence BAG^8^. Our comparisons controlled for standard variables, including age, sex, education, and geography (and cognition in the gaming and tango dancers). Likewise, the pre/post-learning intrasubject design controls all confounding and familial effects with an active control condition. Nonetheless, future studies should examine how the protective effects of creative experiences are potentially influenced by socioeconomic status and other individual-level variables. Finally, our results are consistent with previous findings on the effects of lifestyle^8^ and interventions^16^ on brain aging, including the scalability of these effects^16^. Moreover, they align with the notion that lifestyle and intervention-related changes tend to produce smaller effects than those observed in patient-control comparisons^8,9^.

Overall, our findings provide a framework to study the biological embedding of creative experiences and their association with delayed brain aging. Creativity was associated with reduced brain age in long- and short-term contexts and according to subject-specific skill levels. Creativity-related delayed brain age was especially observed in age-vulnerable regions linked to cognitive processes supporting creativity and expertise, and was associated with brain efficiency and global coupling mechanisms. Our work may inform current calls to increase creativity as a social prescription and interventions in disease and well-being^1,4,37^ by providing specific brain health effects and mechanisms.

## Methods

### Participants and demographics

This report included a total of 1,467 healthy participants. We used data from 1240 healthy controls (HCs) from the EuroLaD EEG Consortium^12^, to train the SVM models, including participants from global settings. Specifically, 724 HCs were from the global north (Turkey, Greece, Italy, the United Kingdom, and Ireland) (age range 17–91 years, mean age 45.7 ± 22.6 years, 48.8% female/male distribution), while 516 were from the global south (Cuba, Colombia, Brazil, Argentina, and Chile) (age range 18–89 years, mean age 46.4 ± 18.5 years, 50% female/male distribution) (Fig. 1a-c, full demographics in Table 1)^55,56^. Inclusion criteria required normal cognitive function and no history of disease. Study protocols were approved by each contributing institution’s Institutional Review Board (IRB), and all participants provided informed consent following the Declaration of Helsinki. The complete demographics are presented in Table 1.

We used independent datasets already published, consisting of 232 participants from studies about creativity expertise (study 1) and pre/post-learning (study 2, Fig. 1c). For study 1, we compared experts and control non-expert participants, considering domains related to dance^47^ (*N* = 46, Argentina), music^49,57^ (*N* = 58, Canada), visual arts^48^ (*N* = 30, Germany), and gaming^26^ (*N* = 62, Poland). Both expert and non-expert participants were age-, sex-, education-, and geography-matched (Table 1). Participants in the gaming group were also matched by working memory capacity (Table S1 in Supplementary Information). Participants in the dancing group were matched by abstraction capacity, working memory, attention, verbal inhibitory control, and verbal working memory (Table S5 in Supplementary Information). Study 2 consisted of measurements before and after a short-term learning session of gaming^29,58^ (*N* = 27, Poland). For all participants considered in this work, sex was defined as the self-reported biological sex. See Table 1 for full demographics and sections Expertise criteria and Pre/post-learning design in Methods for details of expertise criteria and training design. All datasets used in this study were obtained from participants who provided informed consent following the Declaration of Helsinki. The study protocols, including image acquisitions and data collection procedures, were reviewed and approved by the Institutional Review Boards of each contributing institution.

### Study 1. Expertise criteria

#### Tango dancers

A total of 46 right-handed Argentinean participants^47^ completed a self-assessment questionnaire consisting of 20 items designed to measure their level of expertise^47^. Expert and beginner tango dancers were recruited from three tango schools in Buenos Aires — DNI, Flor de Milonga, and Divino Estudio del Abasto Tango school. Exclusion criteria included no past neurological or psychiatric history reported. Inclusion criteria included being right-handed, verified by the Edinburgh Inventory, with normal or corrected-to-normal vision. The self-assessment questionnaire covered various areas, including tango practice, general dance experience, and formal tango instruction. Participants were classified according to their expertise level (Table S2 in Supplementary Information, item 17). We then built two coarser groups, where participants were assigned to the expert tango dancers’ group (*N* = 23), or the non-experts’ one (*N* = 23), using the median months of formal tango instruction as criteria, i.e., >12 months for expert tango dancers. Demographic data are presented in Table 1. The detailed questionnaire items can be found in Table S2 in Supplementary Information. This dataset consisted of 4.4 min resting-state EEG recordings. Ethics for this study was approved by the Comité de Ética of Universidad de San Andrés / CONICET, Buenos Aires, Argentina.

### Musicians

This group included a total of 62 right-handed participants^49^. The expert musicians consisted of 31 participants with 5+ years of experience playing a musical instrument, including professional and amateur musicians (23 and 8 participants each, respectively). The group included various musical expertise across different instruments (e.g., string instruments, percussion) and singing. The non-expert group (non-musicians) consisted of healthy participants who did not play any musical instrument. The information was retrieved from the OMEGA questionnaire using self-declared musical expertise and years of experience. Musicians were selected based on self-reported experience of playing a musical instrument for five or more years. Four participants were excluded due to data quality concerns, leaving a final sample of 29 experts and 29 non-experts. Demographic data are presented in Table 1. This dataset consisted of 5-min resting-state MEG recordings from the OMEGA database^49,57^. MEG recordings and questionnaires are available through the OMEGA repository^57^. The years of education were calculated by translating the maximal educational instruction (e.g., a PhD degree) into cumulative years. Ethics for this study were approved by the Research Ethics Board of Montreal Neurological Institute & Hospital (McGill U.), Montréal, Canada.

### Visual artists

A total of 34 right-handed participants were initially recruited for the study, with 17 visual artists and 17 non-artists. Recruitment occurred through social media, posters in locations related to the topic (e.g., universities, art schools, and art institutions), and word of mouth. After initial email contact, candidates were screened for inclusion and exclusion criteria and asked about their artistic background. Suitable participants were then invited to schedule an EEG session, during which a more detailed questionnaire on their art practice and interests was completed. Exclusion criteria included psychiatric, neurological, or cardiac conditions, and hairstyles or accessories (e.g., dreadlocks) that could impair EEG data acquisition. Following exclusions due to low data quality and non-compliance with inclusion criteria^48^, the final sample consisted of 30 participants: 15 artists (experts) and 15 non-artists (non-experts). As part of the selection criteria, the artists were required to have completed at least three years of university-level academic art education, with specific training in drawing. In contrast, participants in the non-artist group had no formal drawing training and did not engage in drawing regularly. Participants were informed about the data collected and signed informed consent before the session. Compensation was provided, although the specific form or amount is not mentioned in the article. Demographic data are presented in Table 1. This dataset consisted of 2 min resting-state EEG recordings^48^. Ethics for this study was approved by the Ethics Commission of Humboldt-Universität zu Berlin, Germany.

### Gaming

In this study, 62 right-handed male subjects were included^26^. All participants completed an online questionnaire on demographics, education, and video game experience. The online questionnaire was administered via the GEX platform (GEX Immergo, Funds Auxilium Sp. z o.o), which gathered demographic information, education status, and detailed data on video game habits. As part of the questionnaire, participants provided their Battle.net ID, allowing verification of their StarCraft II league ranking and recent gameplay activity. All participants were right-handed males, had no history of neurological illness or psychoactive substance use, and were matched on undergraduate-level education. Working memory capacity was assessed using a modified online version of the operation span (OSPAN) task, with participants required to maintain at least 85% accuracy on the math component to be included. Two participants were excluded due to poor MRI data quality. All participants gave written informed consent and received monetary compensation for their participation. The expert group (*N* = 31) met the following criteria: (a) experienced in real-time strategy video games and StarCraft II, (b) played real-time strategy video games at least 6 hours/week for the past 6 months, (c) spent over 60% of gameplay time on StarCraft II, and (d) actively played in the last two seasons, ranked in one of the StarCraft leagues (Gold, Platinum, Diamond, Master, Grandmaster). The non-expert group (*N* = 31) had: (a) less than 6 hours of real-time strategy play and (b) less than 8 hours per week of total video game play (any genre) in the past 6 months. Only males were recruited due to the lack of female participants with sufficient video game experience. Demographic data are presented in Table 1. Further details about video experience, intellectual level, and other demographic variables can be found in Table S1 in Supplementary Information. As our brain clock models were trained from EEG FC, and this data were not available for participants, we estimated EEG FC from DTI structural connectivity using whole-brain modeling^27,59^. Previous simulated FC from empirical DTI^26^ successfully revealed connectivity differences between players and non-players^27^. We also reported the structural differences between groups in Supplementary Information Figure S6. Ethics for this study was approved by the Research Ethics Committee, SWPS University of Social Sciences & Humanities, Warsaw, Poland.

### Study 2. Pre/post-learning design

A total of 24 right-handed participants were considered for the study in a short learning paradigm using video games^29,58^. Participants were initially recruited online via a covert questionnaire. All participants reported normal or corrected vision, normal hearing, and were right-handed. Exclusion criteria included any history of neurological or psychiatric disorders, head injuries, surgeries, brain tumors, current medication use, or more than five hours of video gaming per week in the prior six months, especially RTS or FPS games. Only participants who completed both sessions and met training requirements were included. Each eligible participant received compensation of approximately 184 USD. Participants engaged in StarCraft II gaming sessions in a controlled laboratory environment at the NeuroCognitive Research Center, SWPS University in Warsaw. Before the first session, each participant completed an introductory training session with a StarCraft II coach to familiarize them with the game’s core concepts and basic mechanics. The training lasted 30 hours in total, spread over 3 to 4 weeks, with participants playing between 5 and 10 hours per week. Telemetric variables were extracted from StarCraft II replays using the Python libraries sc2reader (available at https://github.com/ggtracker/sc2reader) and PACanalyzer (available at https://github.com/Reithan/PACAnalyzer), which enable the retrieval of information from various StarCraft II resources. We used the Actions Per Minute (APM) as a metric of performance, as it is one of the strongest predictors of StarCraft II performance and skill development^40^. APM reflects cognitive, motor, and decision-making speed, increasing as participants gain experience. EEG data were collected during two lab sessions lasting up to two hours each, which included instructions, electrode setup, and an attentional blink task. Only those who completed both sessions and met the training requirements were included in the final analysis. The attentional blink task involved rapid serial visual presentation (RSVP) of letters at the center of the screen. Participants were instructed to detect and report two target letters (T1 and T2) appearing in the stream, with T2 occurring shortly after T1, to measure the transient lapse in attention characteristic of the attentional blink effect. T1 corresponded to a green capital letter (vowel or consonant), and T2 to a black “X” presented at lag 1, 2, or 7 after T1. Participants answered two yes/no questions: whether a vowel (T1) and/or an “X” (T2) appeared. From the test, we reported the T1 and T2 reaction times and accuracy. EEG recordings were acquired before and after 30 hours of training. The total playing time was monitored, and participants were strictly prohibited from playing outside the lab. Demographic data are presented in Table 1. This dataset consisted of 23 min of EEG recordings during an attentional blink task^60^. This task was included to assess generalized attention and temporal processing improvements post-training. Although not directly associated with BAGs, improvements in attentional blink performance may reflect learning-induced changes^60^.

We included an active control group (*N* = 12) trained in Hearthstone. This group was part of the original study design^58^, with identical recruitment, training time, and inclusion/exclusion criteria as the StarCraft II group. The Hearthstone game was selected due to its more rule-based and turn-based mechanics, with limited improvisation and creative play compared to StarCraft II’s real-time decision-making^58^. Ethics for this study was approved by the Research Ethics Committee, SWPS University of Social Sciences & Humanities, Warsaw, Poland.

### M/EEG acquisition, preprocessing, and connectivity

M/EEG data were processed offline using a custom-built automated pipeline (Fig. 1d). The pipeline integrates a mesh model tailored to various electrode arrays and performs source space estimation. Details on acquisition parameters (acquisition time, electrode numbers, sampling rate), are provided in Table S3 in Supplementary Information. The full set of pre/post-processing procedures is available in Supplementary Information sections 1.1-5, with a summary provided below.

The M/EEG signals were re-referenced to an average reference and were resampled to a uniform sampling rate of 512 Hz. EEG preprocessing included re-differentiation, removal of muscle and eye movement artifacts, identification and interpolation of bad channels, and normalization. Source reconstruction was conducted using standardized Low-Resolution Brain Electromagnetic Tomography (sLORETA). Brain regions were defined according to the Automated Anatomical Labeling (AAL) atlas^38^, including only the 78 cortical regions (regions listed in Supplementary Table S4). All MEG data were obtained from a public access data repository (OMEGA^57^). Preprocessing included low-pass filtering, artifact removal, and co-registration of MEG with anatomical images. MEG source estimation was performed using an atlas-based beamforming approach. A dipole-based forward model and beamformer approach were used to estimate time courses for 78 AAL^38^ regions with adjustments for signal polarity. We filtered the M/EEG signals between 8 and 40 Hz using a 3rd-order Bessel filter and then computed Pearson’s correlation between pairs of brain regions, resulting in 78×78 functional connectivity matrices. MEG FCs for musicians and non-musicians were adjusted to the average EEG FC connectivity of a subset of participants from the global north (age range 26−30 years).

The 8–40 Hz band was selected to cover alpha to low-gamma rhythms, which are linked to creative and attentional processes^48^ as well as brain age^12,61^. Although many features derived from M/EEG correlate with brain age (e.g., power spectral density, entropy, kurtosis)^39^, we focused on functional connectivity for specific reasons^8^. Functional connectivity is a robust marker for assessing brain aging, particularly when considering diverse datasets^8,12,61^. It also allows for comparability with fMRI-based brain clock estimations^8^. By using a mesh model and a common source space, we ensured standardized brain mapping across participants^8^. This approach offers a balanced trade-off between spatial and temporal resolution, which is important in multi-site data. In any case, future studies should explore additional M/EEG-derived metrics^39^ to further enrich brain age modeling.

Additionally, we assessed the Overall Data Quality (ODQ) of the EEG recordings using the method developed by Zhao et al.^62^, to discard possible effects of data quality on BAGs’ computation. Despite their differences, BAGs derived from different functional sources can be compared using harmonization steps. The use of a similar approach and a common brain parcellation^38^ allowed us to compare EEG and MEG in the same source space.

### Brain clock models and brain age gaps

To improve the model’s robustness and generalizability^63^, we applied data augmentation over the EEG functional connectivity matrices of the training dataset (Fig. 1d). Using the augmented data, we trained SVMs to predict chronological age^8,9^ (Fig. 1d). A 5-fold cross-validation scheme with up to 15 repetitions was used, and the model performance was assessed by Pearson’s correlation coefficient and the Mean Absolute Error (MAE) between predicted and real chronological ages in the test sets. Feature importance was defined as the absolute value of SVR weight coefficients, averaged across cross-validation folds and repetitions. Detailed methods are provided in Supplementary Information sections 1.6-7. The BAGs were calculated by subtracting the actual chronological age from the SVM-predicted brain age. In the out-of-sample validation, these values were then corrected by regressing the chronological age. The regression slopes and intercepts were estimated from the training data^8,9^. The model performance was not assessed using age bias correction as this step can artificially increase the model performance. We finally normalized the BAGs, subtracting the average BAGs within each domain.

### Expertise scores and BAGs standardization

To compare different expertise scores and their associations with BAGs, we converted BAGs and expertise scores into z-scores to group them on the same scale. This step allows the merging of all the expertise scores on a common scale, ensuring that measures, such as hours per week and years of experience, are expressed under the same scale for consistent comparisons.

### Brain maps and *Neurosynth* associations

From the training data, we correlated nodal functional connectivity with chronological data, representing the brain areas more vulnerable to age, i.e., negatively correlated with age (Fig. 4). We subsequently captured the differences in nodal strength between (a) expert vs non-expert participants across all domains and (b) pre/post-learning connectivity. We reported these differences using Cohen’s D effect size. We then used *Neurosynth*^21^, an automated meta-analytical tool, to explore the cognitive processes linked to altered connectivity associated with creative experiences. We obtained association maps for 89 cognitive terms, which were then parcellated using the AAL atlas. We correlated the changes in nodal strength described above with the *Neurosynth* association maps, reporting the absolute strength of correlations.

To account for potential spatial autocorrelation in brain maps, we conducted Spin Test analyses^64^ using the BrainSMASH Python library (https://brainsmash.readthedocs.io/)^65^. We applied this method to all correlation analyses involving cortical surface data. The Spin Test generates spatially constrained null models by randomly rotating the spherical projection of cortical surface maps while preserving their spatial structure. We used 10,000 permutations to generate null distributions of correlation values. Empirical correlations were then compared to these null distributions to obtain p-values corrected for spatial autocorrelation. We additionally applied false discovery rate (FDR) correction across all comparisons.

### Graph theoretical analyses

We quantified functional network properties associated with creative experiences using tools from graph theory^41^. Functional connectivity matrices were binarized after applying proportional thresholds ranging from 0.02 to 0.1, in steps of 0.01, keeping the highest functional connectivity values after thresholding. We reported the mean value across the whole range of thresholds to minimize the arbitrariness of choosing a single value^41^. From the binarized matrices, we computed the global and local efficiency.

Global efficiency, E, is a measure of network integration and can be related to generalized information processing, that is, coordinated activity throughout the brain. Global efficiency is based on paths and was defined as^66^

1 $${{{\rm{E}}}}=\frac{1}{{{{\rm{n}}}}}{\sum }_{{{{\rm{i}}}}}^{{{{\rm{n}}}}}{{{{\rm{E}}}}}_{{{{\rm{i}}}}}=\frac{1}{{{{\rm{n}}}}}{\sum }_{{{{\rm{i}}}}}^{{{{\rm{n}}}}}\frac{{\sum }_{{{{\rm{j}}}}\ne {{{\rm{i}}}}}^{{{{\rm{n}}}}}{{{{\rm{d}}}}}_{{{{\rm{ij}}}}}^{-1}}{{{{\rm{n}}}}-1}$$

where E_i_ is the nodal efficiency, n is the total number of nodes, and d_ij_ is the shortest path between nodes i and j. Global efficiency ranges between 0 and 1. Higher values indicate highly integrated networks, where nodes can easily transmit information, while values closer to 0 suggest poor integration and more difficult node-to-node communication.

Local efficiency, L, is a measure of network segregation, capturing the efficiency of information transfer within local neighborhoods of the network, which reflects specialized information processing within clusters of interconnected nodes. It is computed as follows^66^

2 $${{{\rm{L}}}}=\frac{1}{{{{\rm{n}}}}}{\sum }_{{{{\rm{i}}}}=1}^{{{{\rm{n}}}}}\frac{1}{{{{{\rm{k}}}}}_{{{{\rm{i}}}}}\left({{{{\rm{k}}}}}_{{{{\rm{i}}}}}-1\right)}{\sum }_{{{{\rm{j}}}},{{{\rm{h}}}}\in \Gamma \left({{{\rm{i}}}}\right)}\frac{{{{{\rm{a}}}}}_{{{{\rm{ij}}}}}{{{{\rm{a}}}}}_{{{{\rm{ih}}}}}}{{{{{\rm{d}}}}}_{{{{\rm{jh}}}}}}$$

where k_i_ is the degree of node i, Γ_i_ is the set of neighbors of node i, and d_jh_ represents the shortest path length between nodes j and h within this neighborhood. Local efficiency ranges from 0 to 1. Higher values indicate efficient communication within local neighborhoods, while values closer to 0 suggest weaker local connections.

Graph theoretical analyses were conducted using the Brain Connectivity Toolbox for Python^41^.

#### Generative whole-brain model

We used a whole-brain model^27,59^ to explore associations between BAGs and biophysical coupling, specifically the global coupling parameter, G, of the model. Global coupling represents the overall conductivity of fibers, reflecting the strength of interregional communication.

Our model integrates structural and functional connectivity with regional dynamics, using a network of 78 brain areas defined by the AAL parcellation. The local brain activity was simulated using the normal form of a supercritical Hopf bifurcation (Stuart-Landau oscillators), which can shift the system’s behavior from self-sustained oscillations (limit cycle) to a stable fixed point.

The model’s key parameters include the global coupling and the local bifurcation parameter, a_i_, which determines whether a region i exhibits noise-induced oscillations (a_i_ < 0), self-sustained oscillations (a_i_ > 0), or critical behavior (a_i_ ≈ 0). We set a_i_ = 0.01 for all regions, following previous studies^27^. Regions were also subjected to uncorrelated Gaussian noise with a standard deviation of β = 0.1. The complete set of equations consisted of

3 $$\frac{{{{\rm{d}}}}{x}_{{{{\rm{i}}}}}\left(t\right)}{{{{\rm{d}}}}t}={{{{\rm{a}}}}}_{{{{\rm{i}}}}}{x}_{{{{\rm{i}}}}}\left(t\right)-\left[{x}_{{{{\rm{i}}}}}^{2}\left(t\right)-{y}_{{{{\rm{i}}}}}^{2}\left(t\right)\right]{x}_{{{{\rm{i}}}}}\left(t\right)-{{{{\rm{w}}}}}_{{{{\rm{i}}}}}{y}_{{{{\rm{i}}}}}\left(t\right) \\+{{{\rm{G}}}}{\sum }_{{{{\rm{j}}}}=1}^{{{{\rm{n}}}}}{{{{\rm{M}}}}}_{{{{\rm{ij}}}}}\left({x}_{{{{\rm{j}}}}}\left(t\right)-{x}_{{{{\rm{i}}}}}\left(t\right)\right)+{{{\rm{\beta }}}}{\eta }_{{{{\rm{i}}}}}\left(t\right)$$

4 $$\frac{{{{\rm{d}}}}{y}_{{{{\rm{i}}}}}\left(t\right)}{{{{\rm{d}}}}t}=\, {{{{\rm{a}}}}}_{{{{\rm{i}}}}}{y}_{{{{\rm{i}}}}}\left(t\right)-\left[{x}_{{{{\rm{i}}}}}^{2}\left(t\right)-{y}_{{{{\rm{i}}}}}^{2}\left(t\right)\right]{y}_{{{{\rm{i}}}}}\left(t\right)-{{{{\rm{w}}}}}_{{{{\rm{i}}}}}{x}_{{{{\rm{i}}}}}\left(t\right) \\+{{{\rm{G}}}}{\sum }_{{{{\rm{j}}}}=1}^{{{{\rm{n}}}}}{{{{\rm{M}}}}}_{{{{\rm{ij}}}}}\left({y}_{{{{\rm{j}}}}}\left(t\right)-{y}_{{{{\rm{i}}}}}\left(t\right)\right)+{{{\rm{\beta }}}}{\eta }_{{{{\rm{i}}}}}(t)$$

Here, *x*(*t*) represents the real component of the M/EEG-like signals, and *y*(*t*) represents the imaginary component. Regions with a_i_ > 0 exhibit self-sustained oscillations at a frequency f_i_ = w_i_/2π, set to 10 Hz across all nodes. The brain regions are coupled via an empirical structural connectivity, M, where each entry M_ij_ indicates the strength of the connection between regions i and j. We used diffusion tensor imaging (DTI) structural connectivity data from the gaming expertise design^26^.

Using the linear approximation of the Hopf model^59^, static functional connectivity was predicted using analytical estimations without running extensive simulations. These estimations can be applied under weak noise conditions and small nonlinearities^59^.

### Model fitting

We generated EEG functional connectivity from DTI structural connectivity and the Hopf model^67^. We swept parameter G between 0 and 3 in steps of 0.1. We compared the simulated and empirical functional connectivity matrices using the structural similarity index (SSIM = 1, perfect fit)^18,68^. In the gaming expertise design, the model was fitted to a subsample of age-matched participants from the training data (24.5 ± 1.0 years). The simulated functional connectivity matrices and G values were used for subsequent analyses. We validated our results by comparing the BAGs between expert and non-expert video game players across different values of G, finding consistent results across the entire parameter range (Fig. S3 and S6 in Supplementary Information). For the remaining groups, we used the average structural connectivity matrix across participants of the gaming expertise design (average across experts and non-experts). We then fitted individual G values using participants’ empirical functional connectivity matrices. The results of the model fitting are presented in Figs. S4, 5 in Supplementary Information.

### Statistical analyses and visualization

Pairwise t-tests for independent samples (expertise) and paired t-tests (pre/post-learning) with a p-value of 0.05 were used to determine significance, plus the 95% confidence intervals and degrees of freedom. Cohen’s D was calculated to provide effect sizes, with values interpreted as small (0.2 < |D| < 0.5), moderate (0.5 <| D| <0.8), large (0.8 < |D| <1.2), and huge (|D| > 1.2). Cohen’s f² was also used to measure effect size in correlations, with values interpreted as small (0.02 < f² < 0.15), moderate (0.15 < f² < 0.35), large (0.35 < f² < 0.5), and huge (f² > 0.5). For categorical variables, like sex, we proceeded with Chi-squared tests. Pearson’s correlation was used to assess statistical relationships, and all p-values from multiple correlations were corrected for false discovery rate (FDR) using the Benjamini-Hochberg method. For generating the brain plots, we used the BrainNet Viewer Toolbox^69^. Word cloud plots were generated with the wordcloud 1.9.3 Python package (https://github.com/amueller/word_cloud). We used one-sided Mann-Whitney U tests in relatively small sample sizes, which limited the suitability of parametric alternatives.

### Sensitivity analyses

Sensitivity analysis 1: To control for potential confounding variables, we conducted an ANCOVA with BAGs as the dependent variable and the expertise (group), age, sex, and education as covariates. This analysis was performed separately for each creative domain (tango dancers, musicians, visual artists, and gaming). In addition, normality tests and matching using parametric and non-parametric tests are reported in Table S7 in Supplementary Information.

Sensitivity analysis 2: We analyzed the effects of age bias correction, comparing the experts and non-experts with and without the correction.

Sensitivity analysis 3: We repeated the spatial correlation analyses between age vulnerability maps and connectivity-based effect sizes separately for each domain of expertise. For the tango dancers, musicians, and visual artists groups, we used the EEG-derived age vulnerability maps; for the gaming group, the age vulnerability map was constructed using MEG data from musicians and non-musicians.

Sensitivity analysis 4: We investigated whether the BAGs are associated with domain-specific expertise levels. Participants were classified as BAG-younger or BAG-older based on the overall BAG distribution, merging experts and non-experts. Specifically, individuals with BAG values below the 35th percentile were labeled as BAG-younger, while those above the 65th percentile were labeled as BAG-older. We then compared expertise scores between these two groups using one-sided Mann-Whitney U tests and FDR correction.

Sensitivity analysis 5: To assess the impact of channel density on model performance, we computed the slope of predicted versus chronological age using linear regression, separately for 64- and 128-channel EEG datasets, in both the training and creativity datasets.

### Reporting summary

Further information on research design is available in the Nature Portfolio Reporting Summary linked to this article.

## Supplementary information

Supplementary Information

Reporting Summary

Transparent Peer review file

## Data Availability

The processed functional‑connectivity matrices, demographic metadata, and full analysis code for all cohorts except the music‑expertise cohort are openly available at both GitHub (https://github.com/carlosmig/Creativity_Brain_Clocks) and the mirrored Zenodo archive 10.5281/zenodo.15915311. These files are sufficient to reproduce every analysis and figure in the paper. The datasets used in this work came from different independent studies: EEG from ReDLat and EuroLad-EEG^12^, tango EEG^47^, visual‑artist EEG^48^, gaming‑expertise DTI^26^, with simulated FC, StarCraftII learners EEG^29,58^, and musicians MEG^49,57^. The music‑expertise cohort’s MEG‑derived functional‑connectivity matrices, demographic variables, and expertise scores are available under restricted access because EUGDPR and original participant consent preclude public release. Qualified academic researchers may obtain these files for non‑commercial research by emailing the corresponding author (agustin.ibanez@gbhi.org) and signing a GDPR‑compliant data‑sharing agreement; requests are acknowledged within ten business days and, once access is granted, no further time limit is placed on data use.

## Code availability

Graph analyses were performed using the Brain Connectivity Toolbox for Python (https://github.com/fiuneuro/brainconn)^41^. *Neurosynth* association maps were generated with the NiMARE Toolbox in Python (https://nimare.readthedocs.io/en/latest/index.html). Full code to reproduce the main results can be found at https://github.com/carlosmig/Creativity_Brain_Clocks and the mirrored Zenodo archive 10.5281/zenodo.15915311. The linearized Hopf model can be found at https://github.com/adrianponce/Linear-Hopf-model^59^. From replays, StarCraft II telemetry data were obtained using sc2reader (https://github.com/ggtracker/sc2reader) and PACanalyzer (https://github.com/Reithan/PACAnalyzer). Brain plots were made using BrainNet Viewer for MATLAB (https://www.nitrc.org/projects/bnv)^69^ and the Python surfplot library (https://pypi.org/project/surfplot/). The spin test analyses^64^ were conducted using the BrainSMASH Python library (https://brainsmash.readthedocs.io/)^65^.
