# Supplementary material for: Creative experiences and brain clocks
Source: Nat Commun. 2025 Oct 3;16:8336. doi: 10.1038/s41467-025-64173-9 (PMC12494922; doi:10.1038/s41467-025-64173-9)
Supplement: Supplementary file 1 — Supplementary Information [file 41467_2025_64173_MOESM1_ESM.pdf]

Supplementary Information for Creative experience and brain clocks

1 Supplementary Methods

1.1 EEG preprocessing and harmonization (tango, visual arts, post/pre-learning)

The EEGs were adjusted to an average reference and band-pass filtered from 0.5 to 40 Hz employing a zero-phase shift Butterworth filter of 8th order. The data sampling rate was increased or decreased to 512 Hz (downsampling or upsampling, according to the case). The standardization of the reference electrode technique was applied for referencing, and eye and muscle artifacts were mitigated using two Independent Component Analysis (ICA)-based techniques. These included ICLabel, which categorizes EEG-independent components into signal or various noise types, and EyeCatch, specifically for detecting eye-movement-related ICA scalp maps. Post-artifact removal, the data underwent manual inspection to spot and correct any faulty channels through weighted spherical interpolation methods.

1.2 EEG data quality

The Overall Data Quality (ODQ) was determined using the approach developed by Zhao et al. EEG recordings were segmented into 1-second intervals, each labeled as "1" for low-quality segments and "0" for high-quality segments. The assessment of each segment's quality was based on four specific metrics. The first metric identifies segments with weak (constant) signals or those with missing or infinite values by examining the standard deviation of the signal. The second metric assesses signal quality by detecting artifacts in the signal amplitude ratio. The third metric evaluates the segment for high-frequency power noise. The fourth and final metric considers the low correlation between channels as an indicator of quality. An EEG segment was classified as of bad quality if it did not surpass, simultaneously, the thresholds for each one of the metrics described: 1) segments with constant (standard deviation of $< 10^{-10}$) and weak (mean absolute deviation $< 10^{-10}\ \mu\mathrm{V}$) signals, and segments with missing or infinite values; 2) absolute amplitude of $150\ \mu\mathrm{V}$; 3) signal-to-noise ratio of 0.5; and 4) maximum correlation between channels of 0.6. The ODQ is then calculated as the percentage of segments that are classified as high quality, with a score of 0 indicating that all segments are of poor quality, and a score of 100 indicating that all segments are of high quality.

1.3 EEG normalization and source functional connectivity estimation

Following protocols for multicenter studies, EEG data were adjusted to minimize variability across different sites. This involved normalizing each dataset independently through Z-score transformation of the EEG time series. The Z-score quantifies the deviation of a data point from the mean, expressed in units of standard deviation.

From the normalized signals, the analysis of resting-state EEG was performed using the standardized Low-Resolution Brain Electromagnetic Tomography (sLORETA) method. sLORETA calculates the standardized current density within predefined virtual sensors located in the cortical gray matter and hippocampus of a standard brain model (MNI 305, Montreal Neurological Institute). This technique determines the current density based on the linear, weighted sum of a specific scalp voltage distribution or the EEG cross-spectrum at the sensor level. As a distributed inverse solution method for EEG, sLORETA standardizes the minimum norm estimation of current density, effectively addressing the challenges of deep EEG source estimation and providing precise localization for specified test regions, despite the high correlation among adjacent sources.

Electrode configurations were mapped onto the MNI152 scalp coordinates. A regularization method, set with a signal-to-noise ratio of 1, was utilized to derive the sLORETA transformation matrix, facilitating the forward operation for the inverse solution. The standardized current density maps were generated using a three-layer concentric sphere head model across a predefined source space of 6,242 voxels (each voxel being $5mm^3$) based on the MNI average brain. Brain segmentation into 78 anatomical cortical areas was conducted using the Automated Anatomical Labeling (AAL) atlas[1], excluding subcortical and cerebellar regions. Current densities for the 153,600 voltage distributions captured during five minutes of resting-state EEG recording (sampled at 512 Hz) were calculated. The voxels within each AAL[1] region were averaged to produce a single mean time series for each cortical area. Signals were bandpass filtered between 8 and 40 Hz. Then, functional connectivity matrices were built using linear Pearson's correlation between pairs of brain areas, generating 78x78 functional connectivity matrices.

1.4 MEG acquisition, preprocessing, and source reconstruction (musicians)

All MEG data analyzed in this study were sourced from the Open MEG Archive (OMEGA), a public access repository[2]. At the time of analysis, the OMEGA repository contained MEG data from 150 healthy participants and self-reported questionnaire data. Participants were classified into two groups based on their self-reported musical experience. The data used for this analysis included 5-minute resting-state MEG recordings, acquired with a 275-channel CTF-MEG system (MISL, Coquitlam, Canada), and sampled at 2400 Hz[2]. During preprocessing, the data were downsampled to 600 Hz. A third-order synthetic gradiometer configuration of the CTF-MEG system was applied, with a 150 Hz low-pass anti-aliasing filter. The OMEGA repository also provided digitized head shapes for each participant (Polhemus). Surface matching between the digitized head shape and the head shape extracted from anatomical scans was performed using in-house software, allowing for accurate co-registration of the MEG sensor geometry and brain anatomy.

For source localization, an atlas-based beamforming approach was applied. The cortex was parcellated into 78 cortical regions using the AAL atlas[4], excluding subcortical and cerebellar regions. A scalar beamformer was used to generate a time course of electrophysiological activity for each region, focusing on the center of mass. Voxel signals were weighted according to their distance from the center of mass using a Gaussian weighting function, ensuring that the time course was biased toward the region's center with a full width at half maximum of ~17 mm.

Covariance was computed over a frequency window of 0.5-150 Hz and across the entire recordings. Tikhonov regularization was applied with a regularization parameter set to 5% of the maximum eigenvalue of the unregularized covariance matrix. The forward model was based on a dipole approximation and a multiple local sphere head model. Dipole orientation was determined through a nonlinear search to maximize the signal-to-noise ratio. The beamformer-estimated time courses were sign-flipped, where necessary, to correct for the arbitrary polarity introduced during the source orientation estimation process. This resulted in 78 electrophysiological time courses, each representing a distinct AAL region, and the procedure was repeated individually for each subject. Signals were bandpass filtered between 8 and 40 Hz. Then, functional connectivity matrices were built using linear Pearson's correlation between pairs of brain areas, generating 78x78 functional connectivity matrices. MEG FC for musicians and non-musicians were adjusted to the average EEG FC connectivity of a subset of participants from the global north (age range 26-30 years).

1.5 MRI and DTI acquisition and preprocessing (gaming expertise)

The original dataset was published originally in previous works of our team[3,4].Detailed acquisition and preprocessing are described below.

MRI data were obtained using a 3-Tesla Siemens Magnetom Trio TIM system (Erlangen, Germany), equipped with a 32-channel phased array head coil. All participants were instructed to minimize head movement during the scan. Initially, anatomical brain images were captured with a T1-weighted MPRAGE sequence, using the following parameters: repetition time (TR) = 2530 ms, echo time (TE) = 3.32 ms, flip angle = 7°, 176 slices, and voxel dimensions of 1 × 1 × 1 mm^3. Following this, diffusion-weighted imaging (DWI) was performed using a spin-echo echo-planar imaging (DW-EPI) sequence, with parameters: TR = 8700 ms, TE = 92 ms, GRAPPA = 2, flip angle = 90°, voxel size = 2 × 2 × 2 mm^3, 64 gradient directions, and a b-value of 1000 s/mm^2. Additionally, two images without diffusion weighting (b = 0) were acquired. The DWI sequence was repeated to enhance the signal-to-noise ratio.

Diffusion tensor imaging (DTI) preprocessing was carried out using the "Pipeline for Analyzing Brain Diffusion Images" (PANDA) software. DICOM files (64 directions) were

converted into a four-dimensional NIFTI format. Images were visually inspected to check for artifacts from head motion. Corrections for eddy currents and motion artifacts were applied via affine alignment of the diffusion-weighted images to the average of the b0 images. Diffusion tensors were computed using weighted least squares, and fractional anisotropy (FA) matrices were generated for each participant using the DTIFIT tool. The native-space FA images were co-registered to the respective T1-weighted structural images via affine transformation, and the structural images were non-linearly normalized to MNI space using the MNI152_T1_2mm_brain template. The transformation was inverted to warp the AAL atlas[1] from MNI space to the T1 space. Each T1 image was co-registered with the native-space FA image, allowing the AAL atlas to be warped to FA space. Based on local gyri and sulci, the brain was then parcellated into 90 regions (45 per hemisphere). Deterministic whole-brain streamlined fiber tractography was performed for each image, using diffusion tensors to trace fiber pathways, with a maximum turning angle of 45° and an FA threshold of 0.2. The white matter tracts were reviewed using TrackVis software (http://trackvis.org/dtk/). A network matrix was constructed by counting the number of fibers connecting pairs of brain regions, with regions serving as nodes and fiber counts as edges. These 90×90 symmetric matrices, one per participant, were normalized between 0 and 1 by dividing each matrix by its global maximum value. We only kept the 78 cortical areas defined in **Table S2**; the resulting connectivity matrices were of dimensions 78x78.

1.6 EEG functional connectivity data augmentation

Data augmentation was used to improve the model's resilience to variability and its ability to generalize. Unlike conventional image augmentation methods, such as random rotations or crops, which do not suit EEG data, our strategy utilized linear interpolation between matrices that correspond to sequential age points.

This method involves calculating a new matrix for a target age that falls between two given ages, a_1 and a_2, using the matrices M_1 and M_2, that represent EEG connectivity at these ages. The interpolation formula used is

$$M_t = (1 - \alpha)M_1 + \alpha M_2 \qquad (1)$$

where $\alpha = (a_t - a_1)/(a_2 - a_1)$ serves as the interpolation factor, facilitating the creation of EEG connectivity matrices for ages not originally present in the dataset. By interpolating, we produce matrices that seamlessly transition between ages, preserving the physiological relevance of the EEG data. We applied data augmentation before training the SVMs with the entire dataset of EEG connectivity for training (N = 1,240). The interpolated data represented 35% of the total data used for training.

1.7 Support vector machine regression and brain age gap estimation

From the augmented data, we trained Support Vector Machines (SVMs) to predict the individuals' chronological age[5]. For training, we used a 5-fold cross-validation (80% training, 20% test) for up to 15 repetitions, where the split in training and test sets was randomized[5]. We performed a grid search to optimize the parameters, evaluating C between 0.1 and 10 and gamma between 0.1 and 10 using an 11x11 log-spaced parameter matrix. C controls the trade-off between maximizing the margin and minimizing classification error, while gamma defines the influence of individual training points. The evaluation of model accuracy involved measuring the Pearson's correlation coefficient, r, and the Mean Absolute Error (MAE) between the predicted ages and the actual chronological ages on the test sets. We used C = 4 and gamma = 0.1 from the grid search. We extracted feature importance by averaging the absolute SVR weights across folds and repetitions, identifying the most predictive connections for brain age.

We computed the BAGs by subtracting the real chronological age from the predicted brain age, the former inferred from SVMs trained with EEG functional connectivity[5]. The BAG serves as a standardized indicator of whether an individual's brain appears biologically older (if the gap is positive) or younger (if the gap is negative)[5]. To correct any bias towards regression to the mean, the chronological age was removed from all calculated BAGs, with the remaining residuals representing the adjusted BAGs, which were used throughout the next analyses. The coefficients used for adjusting BAGs were determined using the training set and then applied to the test set participants. The model performance was not assessed using age bias correction, as this step can artificially increase the model's performance. We finally normalized the BAGs, subtracting the average BAGs within each domain.

2 Supplementary Tables

Table S1. Participants' full demographics and experience with video games for the gaming expertise design.

Variable	Expert (N = 31 males)	Non-experts (N = 31 males)	t values (df = 60)	p values
Age (years)	24.7 (4.27)	24.4 (3.00)	0.320	0.750
Duration of education (years)	15.55 (2.77)	16.10 (2.95)	-0.757	0.452
OSPAN score (WMC)	51.77 (12.73)	51.71 (13.19)	0.018	0.986
Total number of fibers	10,764 (2,567.80)	10,131 (2,445.32)	0.994	0.324
Gaming experience				
Game experience	22.74 (11.78)	2.39 (2.28)	9.443	0.000
StarCraft II experience	18.23 (10.01)	0 (0.00)	10.140	0.000
Real-time strategy	16.06 (9.91)	0.05 (0.15)	8.994	0.000
First-person shooter	1.02 (2.15)	0.27 (0.60)	1.871	0.070
Platform	0 (0.00)	0.06 (0.21)	-1.591	0.122
Fighting	0.16 (0.57)	0 (0.00)	1.563	0.129
Turn-based strategy	1 (1.77)	0.35 (0.70)	1.901	0.065
Sports	0 (0.00)	0.65 (1.42)	-2.549	0.016
Role-play	1.60 (2.08)	0.19 (0.46)	3.685	0.001
Racing	0.13 (0.29)	0.27 (0.55)	-1.254	0.216
Logic	0.66 (1.32)	0.37 (0.66)	1.094	0.280
Multiplayer online battle arena	1.44 (2.99)	0.13 (0.39)	2.419	0.022
Adventure	0.68 (1.88)	0.03 (0.12)	1.921	0.064

Mean and standard deviation (SD in parentheses) represent the values. The term “Game experience” refers to the average number of hours per week spent playing video games in the last 6 months. Similarly, “StarCraft II experience” indicates the average number of weekly hours spent specifically on playing the StarCraft II game over the past 6 months. OSPAN score was used to assess the working memory capacity (WMC). The table was adapted from the original publication[3], and *t* values were calculated using the summary statistics provided there, with two-sided p-values and df degrees of freedom.

Table S2. Questionnaire to evaluate expertise in Tango.

Questions to evaluate expertise degree	Possible answers
a) Tango practicing	
1—Do you currently dance Tango?	Yes/no
2—For how long you have been dancing Tango?	Specified in years, months, and weeks
3—How many hours per week do you dance Tango?	Specified in hours
4—How many hours per month do you dance Tango?	Specified in hours
5—Have you ever received formal Tango instruction?	Yes/No
6—For how long you have received formal Tango instruction?	Specified in years, months, and weeks
7—What style of Tango do you usually perform?	1—Salon/2—Milonguero/3—Free style/4—Electronic/5—Other
b) Dance practicing	
8—Do you perform any other style of dance?	Yes/no
9—Have you ever received formal instruction in any other style of dance?	Yes/no
10—For how long you have received formal instruction in any other style of dance?	Specified in years, months, and weeks
11—Do you dance as a hobby (at discos, parties, etc.)?	Yes/no
12—How many hours per week do you dance as a hobby?	Specified in hours
c) Tango teaching	
13—Do you teach others to dance Tango?	Yes/no
14—How many hours per week do you teach Tango?	Specified in hours
15—Does your main income derive from teaching Tango?	Yes/no

Questions to evaluate expertise degree	Possible answers
16—Do you consider yourself a professional Tango dancer?	Yes/no
17—Do you consider yourself as a(n):	1—Naive/2—Beginner/3—Intermediate/4—Expert
d) Familiarity with observed videos	
18—What is the degree of familiarity that you have with the Tango steps previously observed in the videos?	1—None/2—Know 1 or 2 steps/3—Know half of the steps/4—Know most of the steps/5—Know all of the steps
19—How often do you execute the Tango steps previously observed in the videos?	1—Never/2—Few times a year/3—Few times a month/4—Few times a week/5—Everyday
20—How well do you know Tango Salon style?	1—Not at all/2—Very little/3—Moderately 4—Pretty well/5—Perfectly well

A self-rating questionnaire composed of twenty items to evaluate subjects' expertise degree in three different domains: Tango practicing, dance practicing, and tango teaching. Table adapted from the original publication[6].

Table S3. M/EEG acquisition parameters.

Center	Groups	Type of recording	Time	Eyes	Equipment	Channels	Ref.	Filter	Sampling rate
Centro de Neurociencias de Cuba	Training	EEG	2-20 min	Closed	Digital Electroencephalo gram system MEDICID	64/128 Passive	Linked earlobes	0.5-50 Hz	200 Hz

BrainLat	Training	EEG	5-25 min	Closed	Biosemi ADBOX-MODELO MK2	128 Active	Mastoids	0.16-100 Hz	2048 Hz
Izmir University of Economics and Dokuz Eylül University	Training	EEG	7-10 min	Closed 4 min Open 4 min	BrainAmp	32 Passive	A1 + A2 Electrodes	0.03-70 Hz	500 Hz
Trinity College Dublin	Training	EEG	3-12 min	Closed	Two Biosemi system	264+7 sensors Active	Average	0.1-95 Hz	512 Hz
Universidad de Antioquia	Training	EEG	5-8 min	Closed	Neuroscan Synamps 2	72 Passive	Vertex	0.1-200 Hz	200 Hz
	Training	EEG	7-18 min	Closed	Neuroscan Synamps 2	72 Passive	Right mastoid	0.1-200 Hz	1024 Hz
Universidad de Sao Paulo	Training	EEG	10-26 min	Closed	Braintech 3.0	21	unknown	1-60 Hz	200 Hz
Sapienza Università di Roma	Training	EEG	8-14 min	Closed 5 min Open 5 min	Brain Amp	61 Passive	Fcz Electrode	Highpass 0.016 Hz	1000 Hz
University of Strathclyde	Training	EEG	5-13 min	Closed	Two Biosemi system	64 Active	Left and right mastoids	0.1-35 Hz	512 Hz
Istanbul Medipol University	Training	EEG	2-11 min	Closed 4 min Open 4 min	Brain Amp	32 Passive	A1 + A2 Electrodes	0.01-250 Hz	500 Hz
Takeda	Training	EEG	7-8 min	Closed	Two Biosemi system	132 Active	Left and right mastoids	unknown	1024 Hz
SWPS University of Social Sciences and Humanities	Pre/post-learning	EEG	23 min	Open	SynAmps RT Neuroscan EEG amplifier and BrainProducts actiCap Ag/AG-Cl	64 Active	Average	0.1-40 Hz	250 Hz
Institute of Cognitive Neurology (INECO), C1126AAB Buenos Aires, Argentina	Tango dancers	EEG	4.4 min	Open	Biosemi 128-channel Active Two system (Amsterdam, NLD).	128 Active	Left and right mastoids	0.3-40 Hz	1024 Hz
McConnell Brain Imaging Centre, Montreal Neurological Institute	Musicians	MEG	5 min	Open	CTF-MEG system (MISL, Coquitlam, Canada)	275	26 MEG reference sensors (CTF system setup)	Lowpass 150 Hz	2400 Hz
Berlin School of Mind and Brain, Humboldt-Universität zu Berlin	Visual artists	EEG	2 min	Open	BioSemi Actiview 7.07 (Actiview)	64 Active	Left and right mastoids	0.1-100 Hz	2048 Hz

Detailed specifications for M/EEG data acquisition across various research centers, and groups, outlining the duration of recording sessions, type of recordings (eyes open or closed), the M/EEG equipment used, number of channels, reference electrode placements, filtering parameters, and sampling rates.

Table S4. AAL ROIs.

Brain regions	
Precentral	Cuneus

Superior frontal gyrus, dorsolateral gyrus	Lingual gyrus
Superior frontal gyrus, orbital part	Superior occipital gyrus
Middle frontal gyrus	Middle occipital gyrus
Middle frontal gyrus, orbital part	Inferior occipital gyrus
Inferior frontal gyrus, opercular part	Fusiform gyrus
Inferior frontal gyrus, triangular part	Postcentral gyrus
Inferior frontal gyrus, orbital part	Superior parietal gyrus
Rolandic operculum	Inferior parietal
Supplementary motor area	Supramarginal gyrus
Olfactory cortex	Angular gyrus
Superior frontal gyrus, medial	Precuneus
Superior frontal gyrus, medial orbital	Paracentral lobule
Gyrus rectus	Heschl gyrus
Insula	Superior temporal gyrus
Anterior cingulate and paracingulate gyri	Temporal pole: superior temporal gyrus
Median cingulate and paracingulate gyri	Middle temporal gyrus
Posterior cingulate gyrus	Temporal pole: middle temporal gyrus
Parahippocampal gyrus	Inferior temporal gyrus
Calcarine fissure and surrounding cortex	

78 brain regions of the employed parcellation, from the 116 original regions that belong to the AAL parcellation.

Table S5. Participants' cognitive and behavioral assessments for the dancing expertise design.

Variable	Experts (N = 23)	Non-Experts (N = 23)	t values (df = 44)	p values

Abstraction Capacity (Proverb interpretation)	2.83 (0.28)	2.79 (0.43)	0.406	0.686
Backward Digit Span	4.38 (1.13)	4.31 (1.11)	0.209	0.835
Go-No-Go	2.96 (0.20)	2.93 (0.26)	0.43	0.669
Verbal Inhibitory Control	5.29 (0.86)	5.62 (0.82)	-1.417	0.163
Verbal Working Memory	1.67 (0.64)	1.97 (0.94)	-1.369	0.177

Mean and standard deviation (SD in parentheses) represent the values. Differences between groups were assessed using t-Student tests with two-sided p-values and df degrees of freedom.

Table S6. Blind paradigm task outcomes for the pre/post-learning design.

Variable	Group	Pre	Post	t values	p values	Cohen's D	P (FDR-corrected)

T1 Acc	Active control	219.3 (14.2)	222.6 (17.6)	1.2890, df = 11	0.224	0.3721	0.256
T1 RT (msec)	Active control	658.1 (278.6)	560.8 (254.7)	-1.4452, df = 11	0.176	-0.4172	0.235
T2 Acc	Active control	139.0 (28.1)	148.8 (23.3)	1.4549, df = 11	0.174	0.4200	0.235
T2 RT (msec)	Active control	376.2 (130.7)	296.3 (159.0)	-2.1853, df = 11	0.051	-0.6308	0.137
T1 Acc	Learners	204.7 (41.1)	211.4 (32.4)	0.7950, df = 23	0.435	0.1623	0.435
T1 RT (msec)	Learners	857.0 (502.3)	715.7 (423.8)	-2.9960, df = 23	0.007	-0.6116	0.026
T2 Acc	Learners	136.5 (31.8)	153.7 (25.9)	5.9575, df = 23	< 0.001	1.2161	< 0.001
T2 RT (msec)	Learners	477.0 (485.7)	335.6 (205.7)	-1.7880, df = 23	0.087	-0.3650	0.174

Mean and standard deviation (SD in parentheses) represent the values. Differences between conditions were assessed using two-sided paired t-Student tests with df degrees of freedom. RT: reaction time, Acc: accuracy.

Table S7. Matching and normality testing for the expertise design groups.

Domain	T-test (age)	MWU (age)	T-test (edu)	MWU (edu)	Normality (ages)	Normality (education)
Tango dancers	t=1.32, p=0.1942, df = 44	U=338.50, p=0.1051, df = 44	t=0.48, p=0.6348, df = 44	U=295.00, p=0.5008, df = 44	Experts: p = 0.195. Non-experts: p = 0.006	Experts: p < 0.001. Non-experts: p = 0.189
Musicians	t=0.67, p=0.5077, df = 56	U=439.50, p=0.7731, df = 56	t=1.71, p=0.0931, df = 56	U=525.00, p=0.0919, df = 56	Experts: p = 0.007. Non-experts: p = 0.259	Experts: p = 0.002. Non-experts: p = 0.006
Visual artists	t=0.07, p=0.9418, df = 28	U=109.50, p=0.9167, df = 28	NA	NA	Experts: p = 0.272. Non-experts: p = 0.022	NA
Gaming	t=0.34, p=0.7319, df = 60	U=494.50, p=0.8486, df = 60	t=-0.76, p=0.4531, df = 60	U=415.00, p=0.3559, df = 60	Experts: p = 0.006. Non-experts: p = 0.256	Experts: p = 0.083. Non-experts: p = 0.054

Normality was assessed using the Shapiro-Wilk test, where a *p*-value < 0.05 indicates the lack of normality in the data. All groups are matched by education and age, both using two-sided t-Student and Mann-Whitney (MWU) tests, with df degrees of freedom. All groups are fully matched independently of the chosen test. NA: not available.

Table S8. Sensitivity analysis using ANCOVA.

Groups	Model components		coef	std err	t values	p values	p (FDR-corrected)	df
Tango	Baseline	Intercept	20.8885	10.392	2.01	0.051	-	41
	Confounders	Sex	2.0209	2.797	0.723	0.474	-	
		Age	-0.8504	0.231	-3.677	0.001	-	
		Education	0.549	0.458	1.198	0.238	-	
	Predictor	Expertise	-5.8611	2.563	-2.287	0.027	0.036	
Music	Baseline	Intercept	18.2994	8.789	2.082	0.042	-	53
	Confounders	Sex	1.0512	2.516	0.418	0.678	-	
		Age	-0.506	0.177	-2.862	0.006	-	
		Education	0.0161	0.358	0.045	0.964	-	
	Predictor	Expertise	-4.7632	2.38	-2.002	0.049	0.049	
Visual arts	Baseline	Intercept	22.5092	6.101	3.689	0.001	-	25
	Confounders	Sex	1.3454	2.417	0.557	0.583	-	
		Age	-0.5983	0.213	-2.811	0.009	-	
	Predictor	Expertise	-6.0263	2.041	-2.952	0.007	0.018	
Gaming	Baseline	Intercept	29.7519	4.984	5.97	0	-	57
	Confounders	Age	-0.9793	0.242	-4.049	0	-	
		Education	-0.1125	0.312	-0.361	0.72	-	
	Predictor	Expertise	-3.8048	1.409	-2.701	0.009	0.018	

The model included the brain age gaps (BAGs) as the dependent variable, and the group (expertise), age, education, and sex as predictors. The effect of expertise is significant across all the groups after FDR correction. We used two-sided p-values with df degrees of freedom.

Table S9. Domain-specific differences in expertise between BAG-younger and BAG-older groups.

Groups	BAG-younger expertise Mean (SD)	BAG-older expertise Mean (SD)	MWU	p-value	p (FDR-corrected)	df
Tango dancers	16.32 (13.63)	5.10 (6.58)	118.5	0.012	0.016	44
Musicians	3.64 (2.50)	0.86 (1.91)	26.5	0.030	0.030	56
Visual artists	8.20 (8.48)	3.45 (6.49)	138	0.008	0.016	28
Gaming	40.06 (46.18)	18.81 (46.37)	69.5	0.001	0.005	60

We classified participants as BAG-younger or BAG-older based on the overall BAG distribution (combining experts and non-experts). Specifically, individuals with BAG values below the 35th percentile were labeled as BAG-younger, while those above the 65th percentile were labeled as BAG-older. Mean and standard deviation (SD in parentheses) represent the values. Differences between groups were assessed using one-size Mann-Whitney (MWU) tests. *p*-values were FDR-corrected with df degrees of freedom.

Table S10. Comparison of prediction slopes by EEG sensor density.

Training data				
Channels	Slope	t values	p	df
64	0.922 (0.002)	1.27	0.213	110
128	0.921 (0.003)			
**Creativity data**				
Channels	Slope	t values	p	df
64	1.746 (0.0344)	-1.28	0.211	110
128	1.764 (0.0395)			

Slopes of predicted versus actual age were compared between 64- and 128-channel EEG data in training and creativity datasets. Numbers in “Slope” represent mean values across repetitions and folds ± standard deviation in parentheses. We used t-Student tests with two-sided p-values and df degrees of freedom.

Table S11. BAG differences in the pre/post-learning design.

BAG differences (pre/post-learning design)					
Group	ΔBAGs (years)	U	p	D	df
Learners	-3.115 (5.12)	90	0.0362	-0.49	34
Active control	0.057 (6.88)				

ΔBAGs represent the difference, in terms of BAGs, between the post- and pre-training conditions, with the standard deviation in parentheses. Significantly delayed brain age was observed in the learners in comparison with the active control group: one-sided Mann-Whitney test (Δ(ΔBAGs) = -3.152, U = 90, p = 0.036, D = -0.49).

3 Supplementary Figures

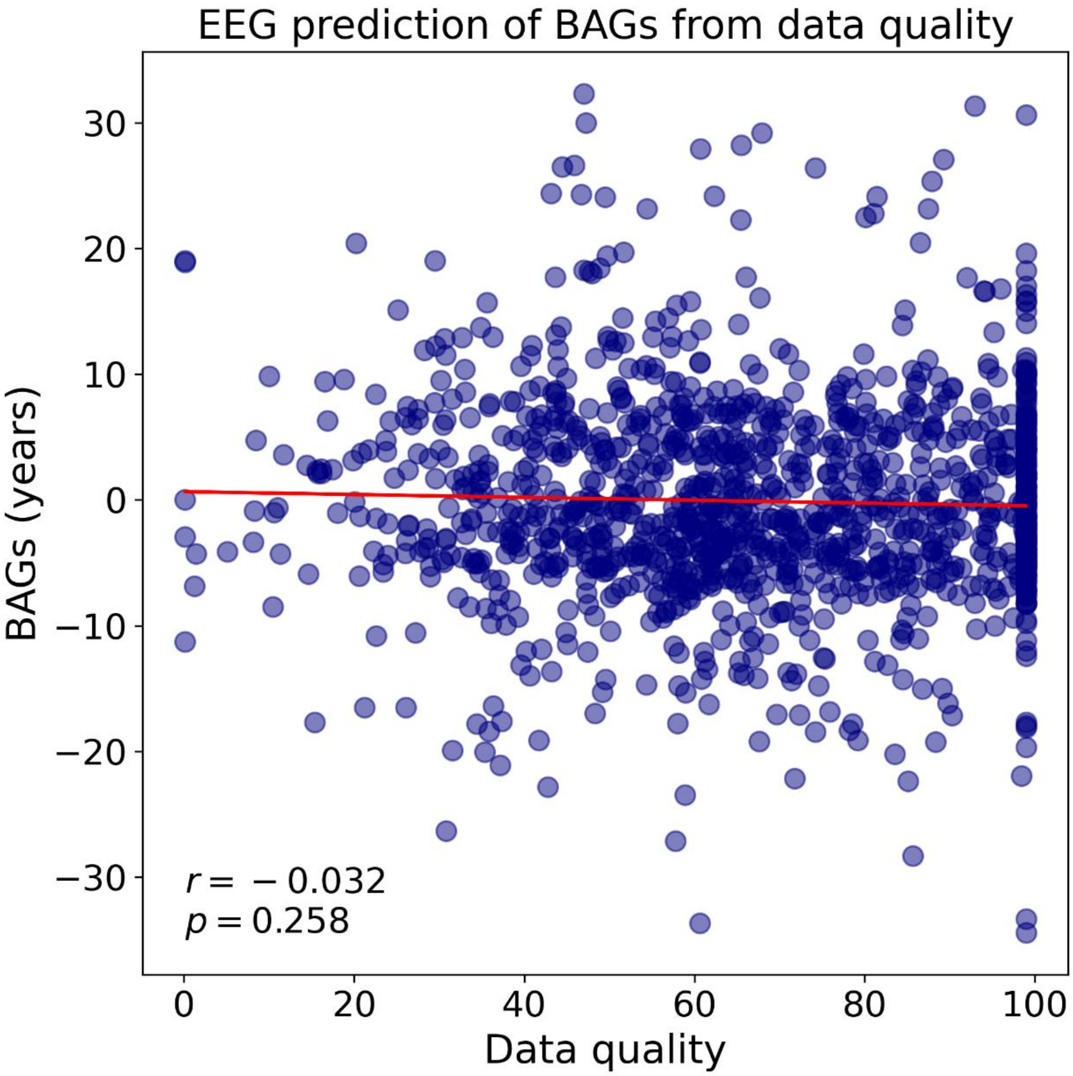

Figure S1. Prediction of the brain age gaps (BAGs) from EEG data quality. Using the training data (N = 1,240 participants, without considering data augmentation) we found no associations between BAGs and data quality ($r = -0.032, p = 0.258, \text{Cohen's } f^2 = 0.001, df = 1238$). Points represent participants.

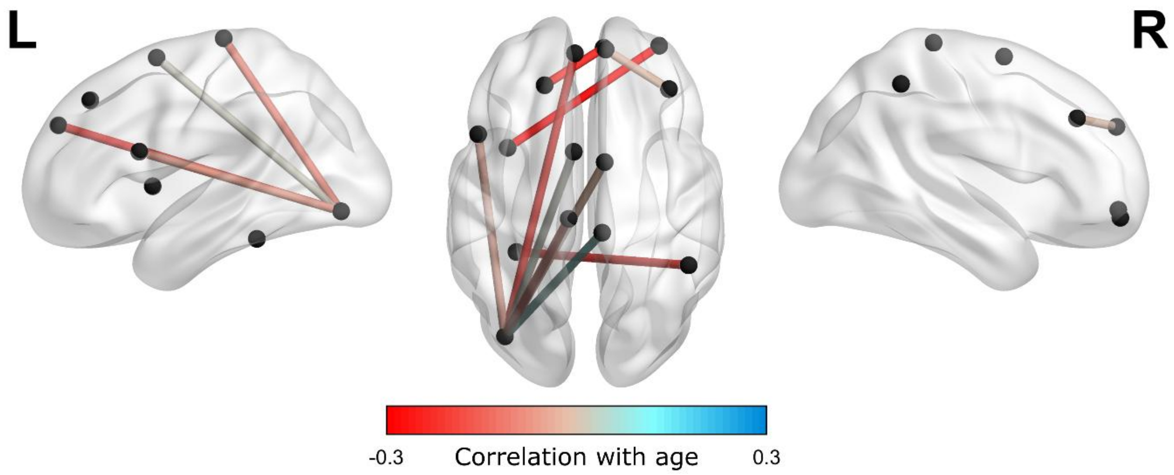

Figure S2. Brain age network. The connections here represent the top 10 features for predicting age using the training data. These connections are the same as the ones described in Figure 2b. Red and blue colors represent negative and positive correlations, respectively. Here, the top 10 most relevant features/connections for predicting age are, almost all of them, negatively correlated with age.

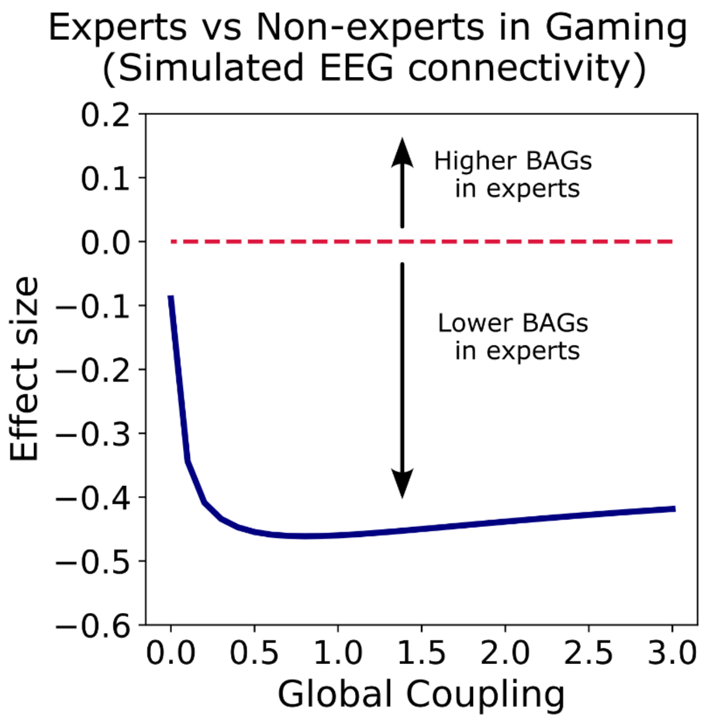

Figure S3. BAGs comparison between video game experts and non-expert participants using simulated data. As a validation, we compared the BAGs between experts and non-experts for the different values of the global coupling parameter. We aimed to test if the differences were consistent across the whole parameter range. We made those comparisons using Cohen's D effect size. We ran simulations for each participant using their structural connectivity matrices and the linearized version of the Hopf model. We found consistent results, i.e., lower BAGs (negative Cohen's D values) in experts versus non-experts, for global coupling values > 0.5.

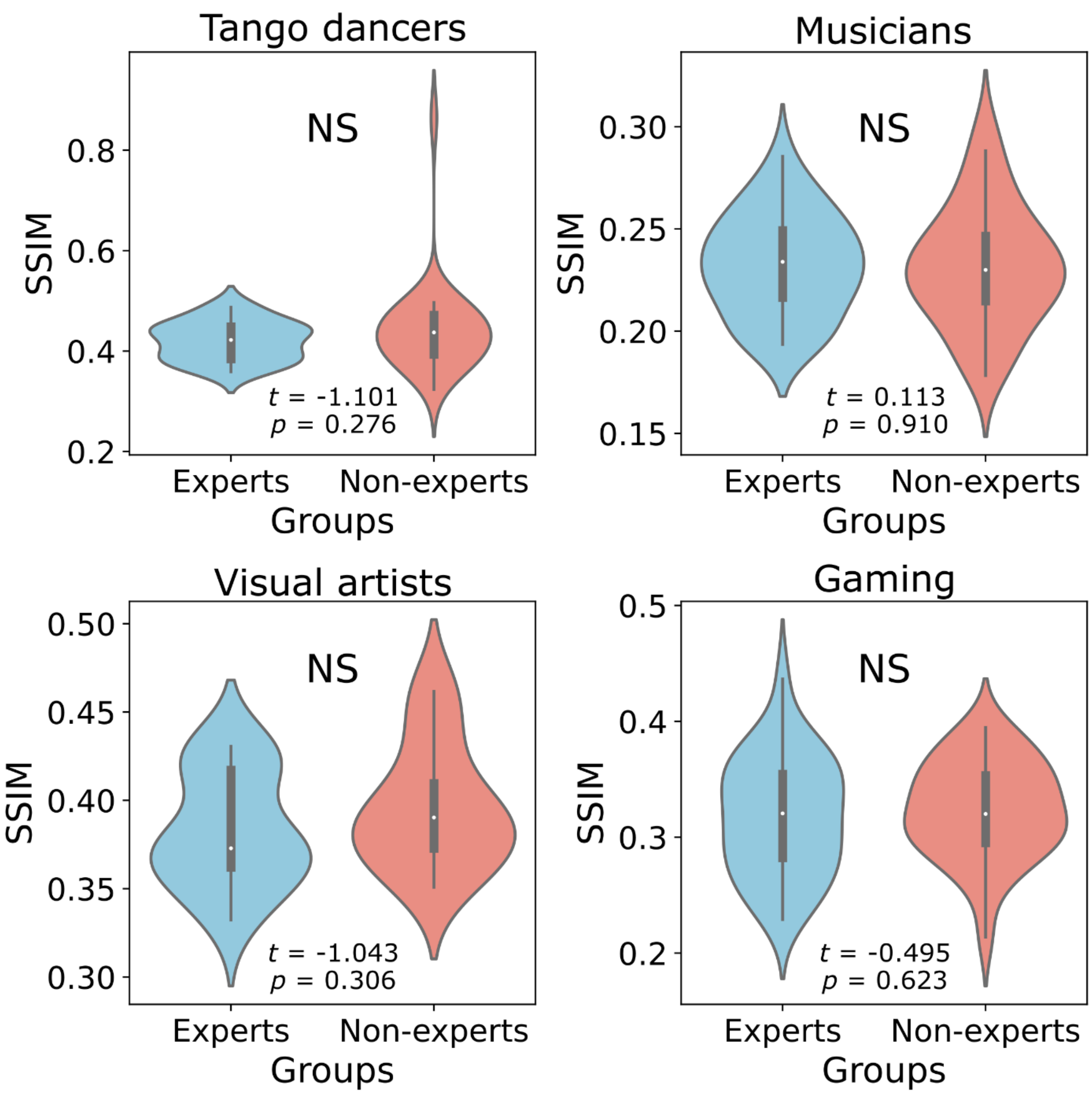

Figure S4. The goodness of fit of the whole-brain model across different groups of creative expertise. We used the structural similarity index (SSIM) to measure the similarity between simulated and empirical functional connectivity matrices. We found non-significant (NS) differences between the SSIM of experts versus non-expert participants across all domains. Box plots show the median and the first and third quartiles; whiskers mark the minimum and maximum values. We used t-Students tests with two-sided p-values and df degrees of freedom. Sample sizes are N = 23 experts and N = 23 non-experts for tango dancers (df = 44), N = 29 experts and N = 29 non-experts for musicians (df = 56), N = 15 experts and N = 15 non-experts for visual artists (df = 28), N = 31 experts and N = 31 non-experts for gaming (df = 60).

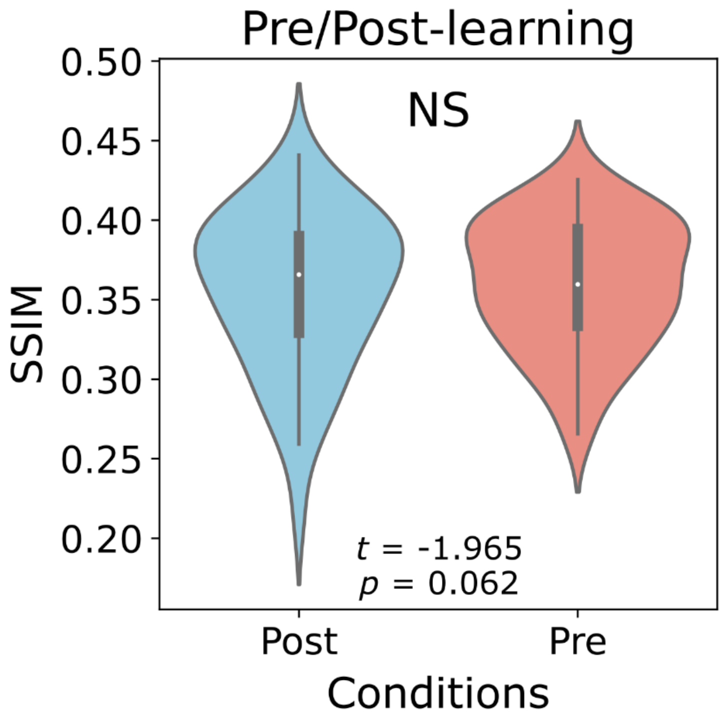

Figure S5. The goodness of fit of the whole-brain model for the pre/post-learning design (N = 24 participants, 23 degrees of freedom). We used the structural similarity index (SSIM) to measure the similarity between simulated and empirical functional connectivity matrices. We found non-significant (NS) differences between SSIMs of pre- and port-learning conditions. Box plots were built using the median, 1st and 3rd quartiles, and the maximum and minimum values of the distributions. We used paired t-Students tests with two-sided p-values. Box plots show the median and the first and third quartiles; whiskers mark the minimum and maximum values.

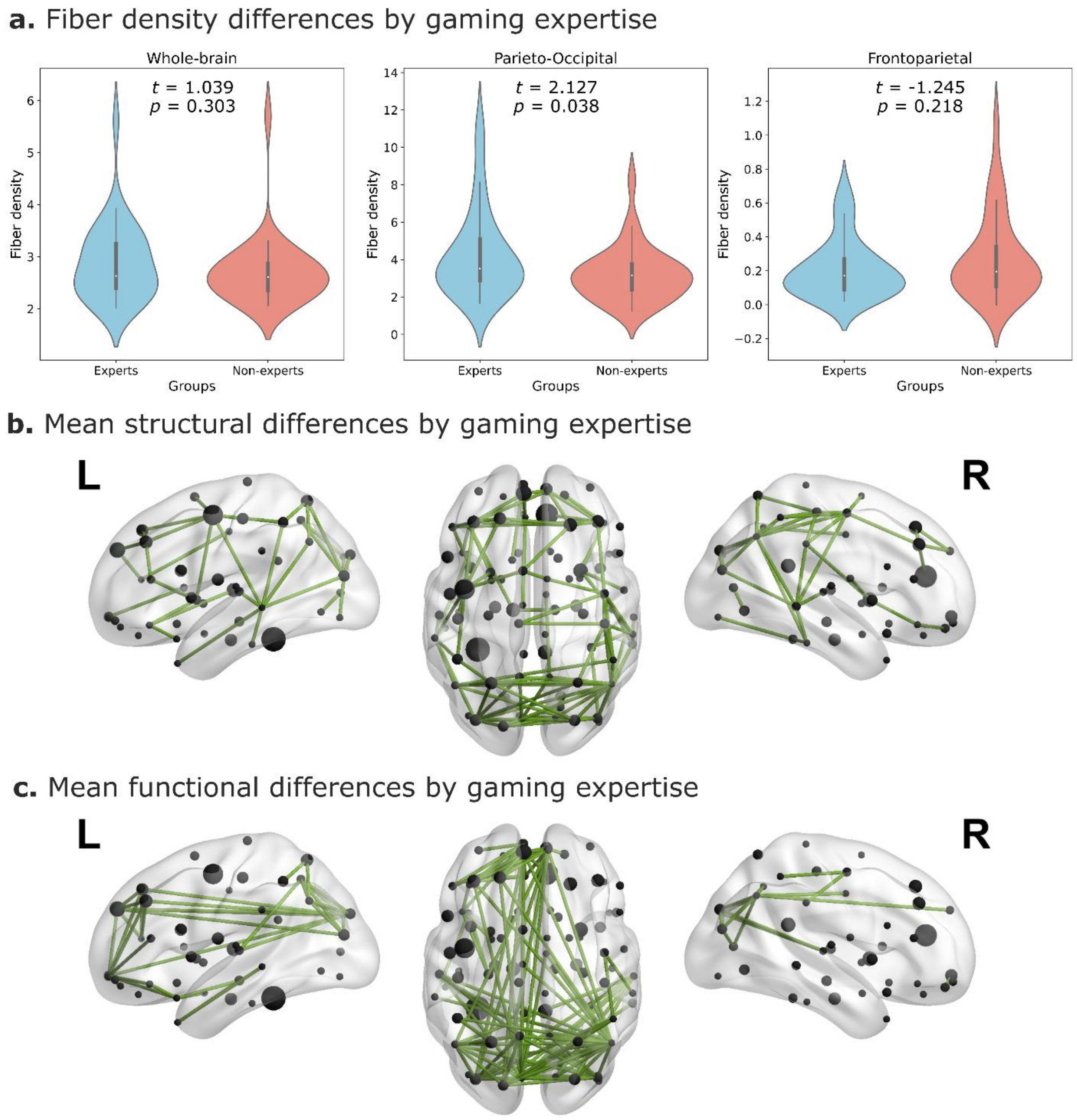

Figure S6. Structural and functional differences between expert and non-expert video game players. **a.** From the empirical structural connectivity matrices, we compared the mean number of fibers between experts (N = 31) and non-experts (N = 31), finding no significant differences at the whole brain level and between frontal and parietal regions, and a higher fiber density between parietal and occipital regions in experts than non-experts. **b.** DTI structural connectivity differences between experts and non-experts. **c.** EEG functional connectivity differences between experts and non-experts. We used t-Student tests with two-sided p-values with 60 degrees of freedom. Box plots show the median and the first and third quartiles; whiskers mark the minimum and maximum values.

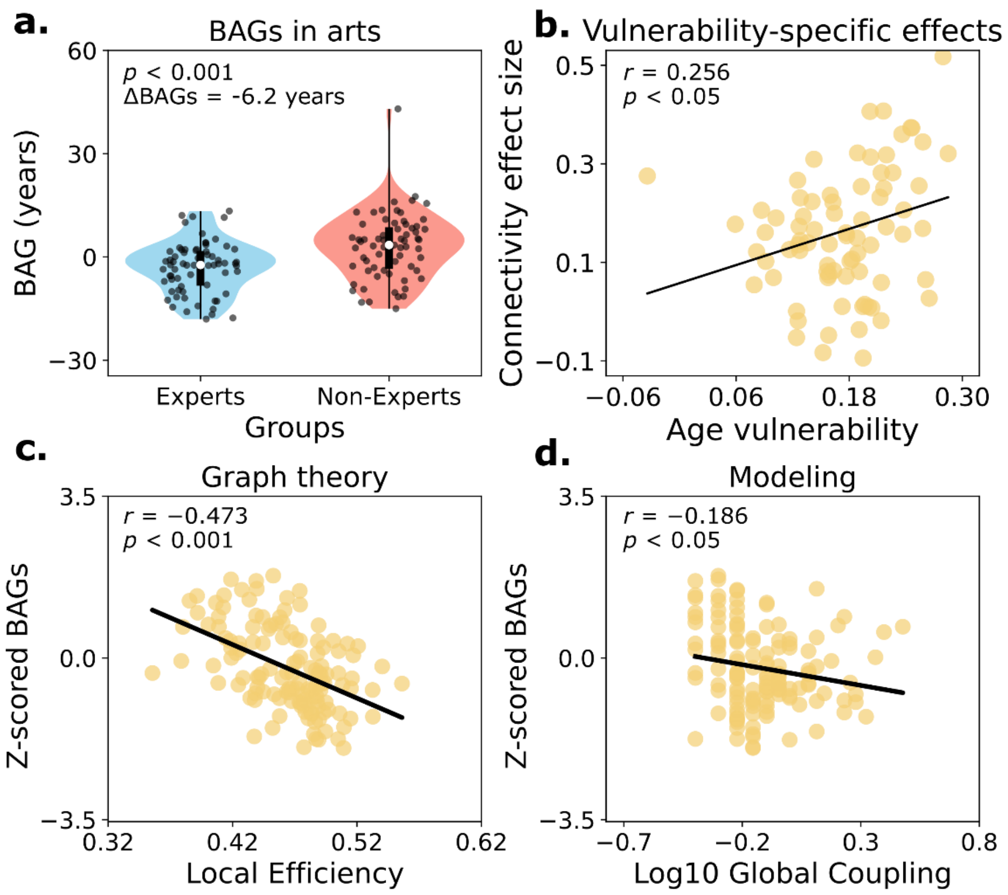

Figure S7. Validation of analyses using only artistic creativity datasets: tango dancers, musicians, and visual artists. **a.** BAGs between experts and non-experts (ΔBAGs = -6.2, t = -4.163, p < 0.001, D = -0.72, FDR-corrected) (N = 83 experts, N = 83 non-experts, 164 degrees of freedom). Correlations between **b.** vulnerability-specific effects and brain connectivity (r = 0.256, p = 0.016, Cohen's f^2 = 0.070, spin test, FDR-corrected, 77 degrees of freedom); **c.** local efficiency (segregation) and BAGs (r = -0.473, p < 0.001, Cohen's f^2 = 0.288, FDR-corrected, 164 degrees of freedom); **d.** Global coupling and BAGs (r = -0.186, p = 0.032, Cohen's f^2 = 0.036, FDR-corrected, 164 degrees of freedom). N = 166 participants for scatterplots. Box plots show the median and the first and third quartiles; whiskers mark the minimum and maximum values, and points represent participants. We used t-Student tests with two-sided p-values.

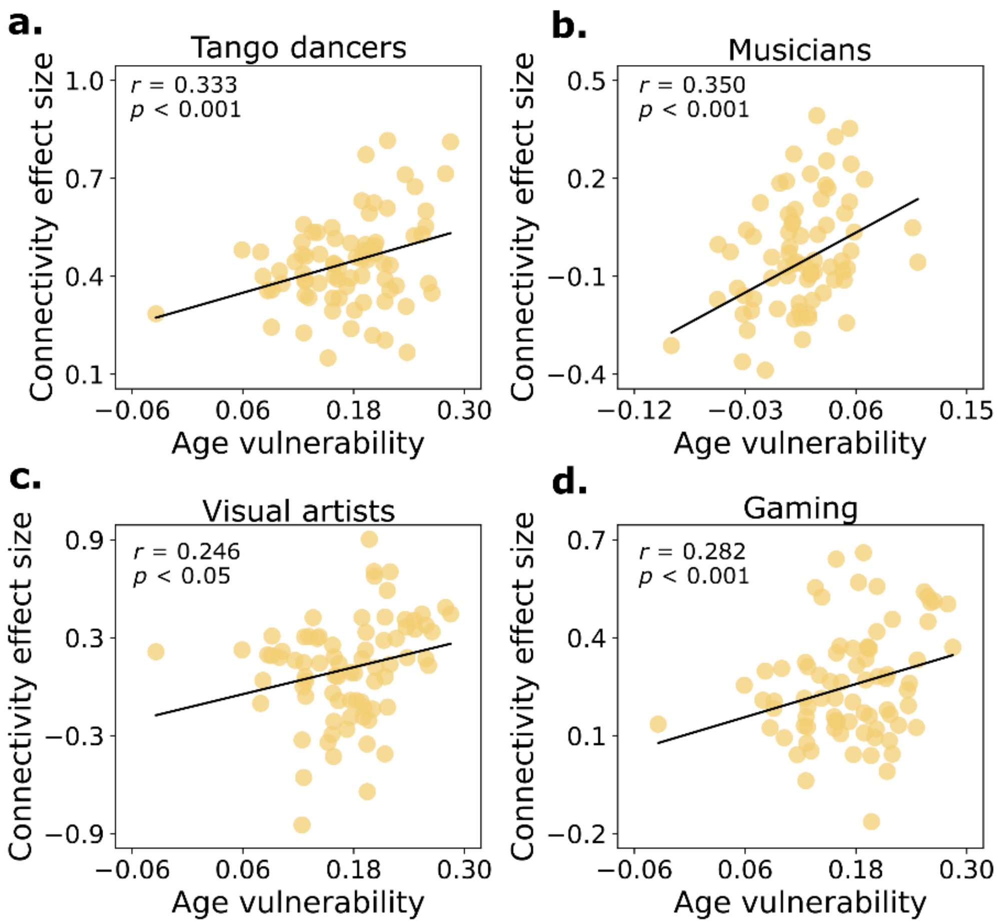

Figure S8. Spatial correlations for each domain in the expertise design. Age vulnerability versus connectivity effect size in **a.** tango dancers (r = 0.333, p = 0.002, Cohen's f^2 = 0.125, df = 77); **b.** musicians (r = 0.350, p = 0.001, Cohen's f^2 = 0.140, df = 77); **c.** visual artists (r = 0.246, p = 0.013, Cohen's f^2 = 0.064, df = 77); **d.** gaming (r = 0.282, p = 0.01, Cohen's f^2 = 0.086, df = 77). We used the EEG-derived age vulnerability map for groups related to tango dancers, musicians, and visual artists. For music expertise, we built the age vulnerability map from musicians' and non-musicians' MEG data. Points represent participants. Before FDR correction, the *p*-values were computed using the Spin test up to 10000 permutations.

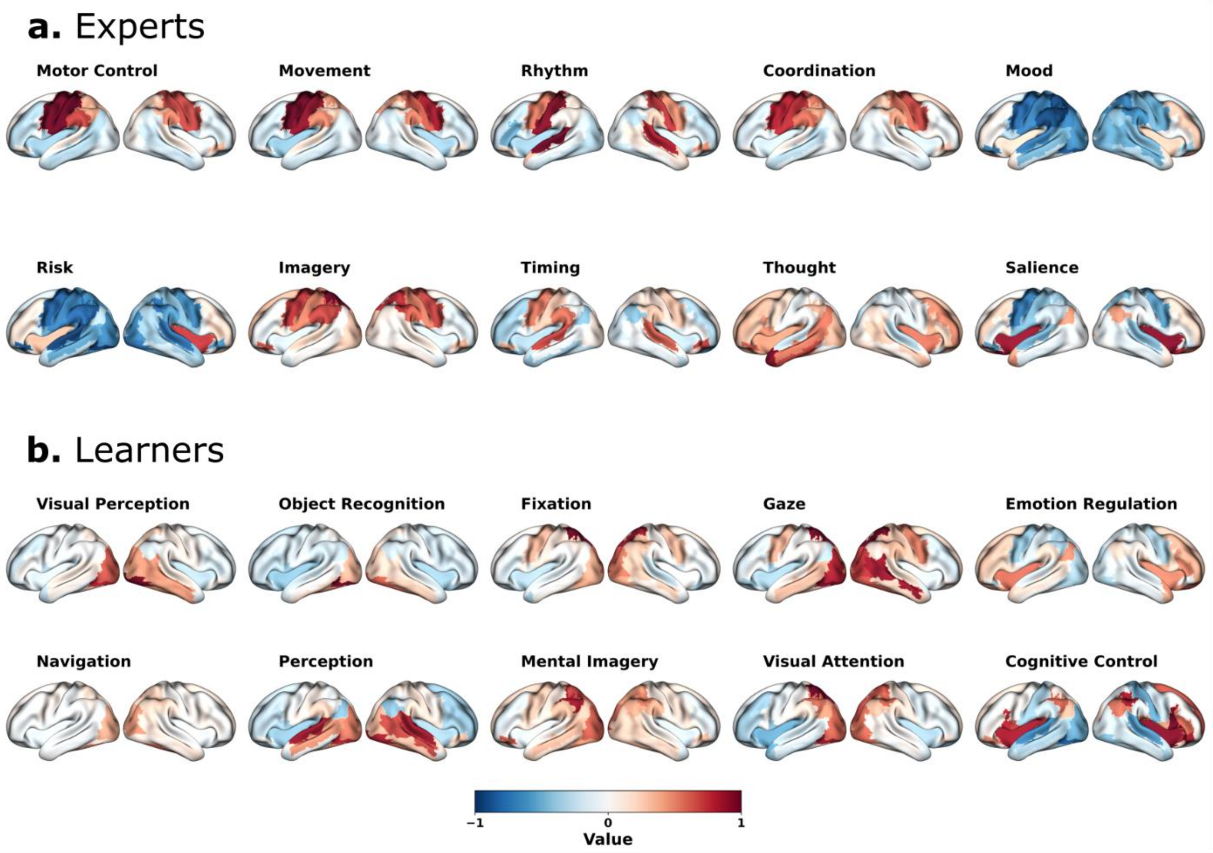

Figure S9. Brain association maps associated with the top 10 correlations with cognitive terms and connectivity effect size in **a.** experts and **b.** learners. For visualization purposes, all maps were normalized to be in the range [-1,1].

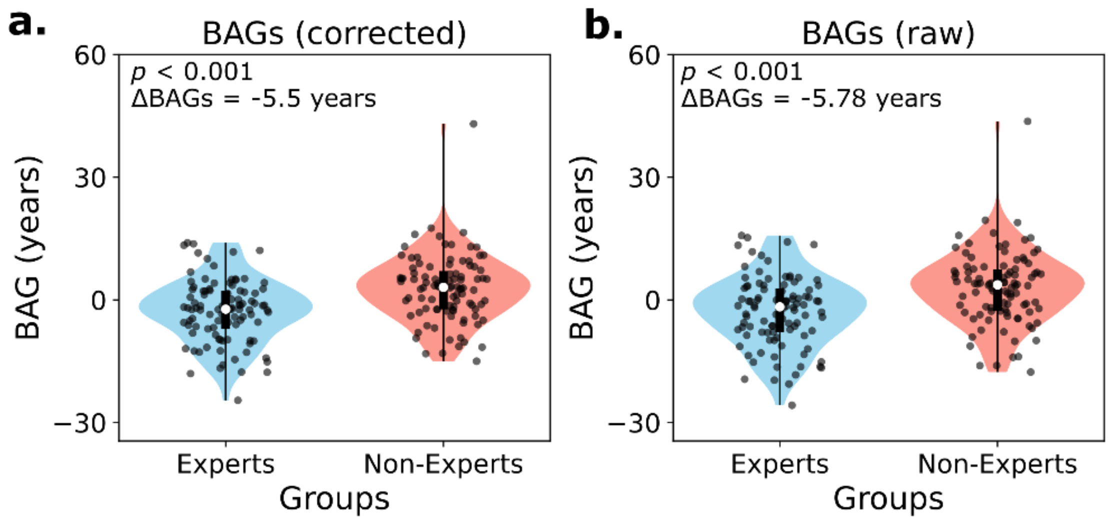

Figure S10. BAGs with and without age bias correction. We compared all experts and non-experts combined. Results are similar regardless of the approach employed. **a.** BAGs with age bias correction: ΔBAGs = -5.50, t = -4.823, p < 0.001, D = -0.69, FDR-corrected, df = 194. **b.** BAGs without age bias correction: ΔBAGs = -5.78, t = -4.625, p < 0.001, D = -0.66, FDR-corrected, df = 194. Results remained unchanged after removing outliers, i.e., values 2 standard deviations from the mean, for **a.** with correction (t = -5.496, p < 0.001), and **b.** without correction (t = -5.621, p < 0.001). Box plots show the median and the first and third quartiles; whiskers mark the minimum and maximum values, and points represent participants. We used t-Student tests with two-sided p-values and df degrees of freedom.

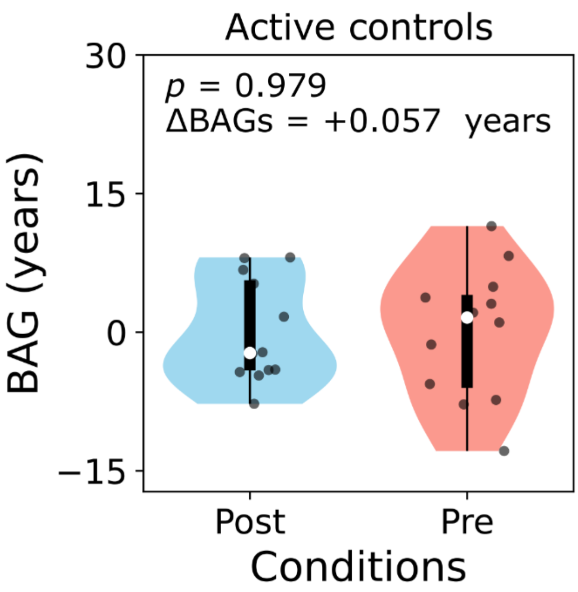

Figure S11. BAGs for the pre/post-learning design active control group. We compared the BAGs post- and pre-training with Hearthstone. We found no BAG differences between the two conditions (ΔBAGs = 0.057, t = 0.0274, p = 0.979, D = 0.0092, FDR-corrected, df = 34). Box plots show the median and the first and third quartiles; whiskers mark the minimum and maximum values, and points represent participants. We used paired t-Student tests with two-sided p-values and df degrees of freedom.

4 Supplementary references

1 Tzourio-Mazoyer, N. *et al.* Automated Anatomical Labeling of Activations in SPM Using a Macroscopic Anatomical Parcellation of the MNI MRI Single-Subject Brain. *NeuroImage* **15**, 273-289 (2002). https://doi.org/10.1006/nimg.2001.0978

2 Niso, G. *et al.* OMEGA: the open MEG archive. *Neuroimage* **124**, 1182-1187 (2016).

3 Kowalczyk, N. *et al.* Real-time strategy video game experience and structural connectivity–A diffusion tensor imaging study. *Human brain mapping* **39**, 3742-3758 (2018).

4 Coronel-Oliveros, C. *et al.* Gaming expertise induces meso-scale brain plasticity and efficiency mechanisms as revealed by whole-brain modeling. *NeuroImage* **293**, 120633 (2024).

5 Tian, Y. E. *et al.* Heterogeneous aging across multiple organ systems and prediction of chronic disease and mortality. *Nature Medicine* **29**, 1221-1231 (2023). https://doi.org/10.1038/s41591-023-02296-6

6 Amoruso, L. *et al.* Time to Tango: Expertise and contextual anticipation during action observation. *NeuroImage* **98**, 366-385 (2014). https://doi.org/10.1016/j.neuroimage.2014.05.005
