# Supplementary material for: Creative experiences and brain clocks
Source: Nat Commun. 2025 Oct 3;16:8336. doi: 10.1038/s41467-025-64173-9 (PMC12494922; doi:10.1038/s41467-025-64173-9)
Supplement: Supplementary file 2 — Reporting Summary [file 41467_2025_64173_MOESM2_ESM.pdf]

nature portfolio

Corresponding author(s): Agustin Ibañez

Last updated by author(s): Jul 14, 2025

Reporting Summary

Nature Portfolio wishes to improve the reproducibility of the work that we publish. This form provides structure for consistency and transparency in reporting. For further information on Nature Portfolio policies, see our Editorial Policies and the Editorial Policy Checklist.

Statistics

For all statistical analyses, confirm that the following items are present in the figure legend, table legend, main text, or Methods section.

n/a	Confirmed	
☐	☒	The exact sample size (*n*) for each experimental group/condition, given as a discrete number and unit of measurement
☐	☒	A statement on whether measurements were taken from distinct samples or whether the same sample was measured repeatedly
☐	☒	The statistical test(s) used AND whether they are one- or two-sided<br>*Only common tests should be described solely by name; describe more complex techniques in the Methods section.*
☐	☒	A description of all covariates tested
☐	☒	A description of any assumptions or corrections, such as tests of normality and adjustment for multiple comparisons
☐	☒	A full description of the statistical parameters including central tendency (e.g. means) or other basic estimates (e.g. regression coefficient) AND variation (e.g. standard deviation) or associated estimates of uncertainty (e.g. confidence intervals)
☐	☒	For null hypothesis testing, the test statistic (e.g. *F*, *t*, *r*) with confidence intervals, effect sizes, degrees of freedom and *P* value noted<br>*Give P values as exact values whenever suitable.*
☒	☐	For Bayesian analysis, information on the choice of priors and Markov chain Monte Carlo settings
☒	☐	For hierarchical and complex designs, identification of the appropriate level for tests and full reporting of outcomes
☐	☒	Estimates of effect sizes (e.g. Cohen's *d*, Pearson's *r*), indicating how they were calculated

Our web collection on statistics for biologists contains articles on many of the points above.

Software and code

Policy information about availability of computer code

Data collection	The M/EEG signals were re-referenced to an average reference and were resampled to a uniform sampling rate of 512 Hz. EEG preprocessing included re-differentiation, removal of muscle and eye movement artifacts, identification and interpolation of bad channels, and normalization. Source reconstruction was conducted using standardized Low-Resolution Brain Electromagnetic Tomography (sLORETA). Brain regions were defined according to the Automated Anatomical Labeling (AAL) atlas, including only the 78 cortical regions (regions listed in Supplementary Table S4). All MEG data were obtained from a public access data repository (OMEGA). Preprocessing included low-pass filtering, artifact removal, and co-registration of MEG with anatomical images. MEG source estimation was performed using an atlas-based beamforming approach. A dipole-based forward model and beamformer approach were used to estimate time courses for 78 AAL regions with adjustments for signal polarity. We filtered the M/EEG signals between 8 and 40 Hz using a 3rd-order Bessel filter, and then computed the Pearson correlation between pairs of brain regions, resulting in 78x78 functional connectivity matrices.
Data analysis	Graph analyses were performed using the Brain Connectivity Toolbox for Python (https://github.com/fiuneuro/brainconn). Neurosynth association maps were generated with the NiMARE Toolbox in Python (https://nimare.readthedocs.io/en/latest/index.html). Full codes to reproduce the main results can be found at https://github.com/carlosmig/Creativity_Brain_Clocks. The linearized Hopf model can be found at https://github.com/adrianponce/Linear-Hopf-model. From replays, StarCraft II telematic data were obtained using sc2reader (https://github.com/ggtracker/sc2reader) and PACanalyzer (https://github.com/Reithan/PACAnalyzer). Brain plots were made using BrainNet Viewer for MATLAB (https://www.nitrc.org/projects/bnv) and the Python surfplot library (https://pypi.org/project/surfplot/). The spin test analyses were conducted using the BrainSMASH Python library (https://brainsmash.readthedocs.io/).

For manuscripts utilizing custom algorithms or software that are central to the research but not yet described in published literature, software must be made available to editors and reviewers. We strongly encourage code deposition in a community repository (e.g. GitHub). See the Nature Portfolio guidelines for submitting code & software for further information.

Data

Policy information about availability of data

All manuscripts must include a data availability statement. This statement should provide the following information, where applicable:

- Accession codes, unique identifiers, or web links for publicly available datasets
- A description of any restrictions on data availability
- For clinical datasets or third party data, please ensure that the statement adheres to our policy

The processed functional-connectivity matrices, demographic metadata, and full analysis code for all cohorts except the music-expertise cohort are openly available at both GitHub (https://github.com/carlosmig/Creativity_Brain_Clocks) and the mirrored Zenodo archive https://doi.org/10.5281/zenodo.15915311. These files are sufficient to reproduce every analysis and figure in the paper. The datasets used in this work came from different independent studies: EEG from ReDLat and EuroLad-EEG55, tango EEG47, visual-artist EEG48, gaming-expertise DTI26, with simulated FC, StarCraft II learners EEG29,59, and musicians MEG49,58. The music-expertise cohort's MEG-derived functional-connectivity matrices, demographic variables, and expertise scores are available under restricted access because EU GDPR and original participant consent preclude public release. Qualified academic researchers may obtain these files for non-commercial research by emailing the corresponding author (agustin.ibanez@gbhi.org) and signing a GDPR-compliant data-sharing agreement; requests are acknowledged within ten business days and, once access is granted, no further time limit is placed on data use.

Research involving human participants, their data, or biological material

Policy information about studies with human participants or human data. See also policy information about sex, gender (identity/presentation), and sexual orientation and race, ethnicity and racism.

Reporting on sex and gender	695 females, 721 males. Sex-specific analyses were performed in this work. We have some missing values about sex (SVMs training data).<br><br>Sex was matched across groups but not analyzed further because it was not a study variable.
Reporting on race, ethnicity, or other socially relevant groupings	For training the SVMs -> Country of origin was reported: N = 724 for Global North healthy controls, N = 516 for Global South healthy controls.<br><br>N = 232 for creativity groups, from Germany, Canada, Argentina and Poland.
Population characteristics	Participants' age ranged from 17 to 91 years, with a mean of 48.82 and 21.43 years of standard deviation.
Recruitment	Participants were recruited as a part of a multisite study including centers from Global North (Turkey, Greece, Italy, the United Kingdom, and Ireland) and South (Cuba, Colombia, Brazil, Argentina, and Chile). Creativity groups participants were recruited from Germany, Canada, Argentina and Poland.<br><br>We reused five published cohorts, each recruited by the original authors. Tango experts and matched novices volunteered through three Buenos Aires tango schools after completing a self-assessment questionnaire. Musicians and controls came from the Canadian OMEGA project, where healthy adults self-enrolled for MEG recordings. Visual artists and non-artists were recruited in Berlin via social-media adverts and posters at art schools and universities. Polish StarCraft II experts and non-experts were drawn from an online gaming platform (GEX) through a detailed questionnaire. The learning group and its active controls were Poles who responded to an online call for novices willing to complete a 30-hour laboratory StarCraft II or Hearthstone programme at the SWPS NeuroCognitive Research Center.<br><br>Monetary compensation was given to the visual-artist cohort, the StarCraft II expertise cohort, and both the StarCraft II learner and Hearthstone control groups; the tango, musician, and EuroLaD participants were not paid in the original studies.
Ethics oversight	The Institutional Review Board (IRB) of each contributing institution approved the study protocols and image acquisitions, ensuring all participants provided informed consent by the Declaration of Helsinki.<br><br>The musicians MEG study was cleared by the Research Ethics Board of the Montreal Neurological Institute & Hospital (McGill University); the tango study by the Comité de Ética of Universidad de San Andrés / CONICET; the RTS-gaming DTI and attentional-blink studies by the Research Ethics Committee of SWPS University of Social Sciences and Humanities; and the visual-artist EEG study by the Ethics Commission of Humboldt-Universität zu Berlin, noting in every case that participants provided written informed consent under the Declaration of Helsinki.

Note that full information on the approval of the study protocol must also be provided in the manuscript.

Field-specific reporting

Please select the one below that is the best fit for your research. If you are not sure, read the appropriate sections before making your selection.

☒ Life sciences ☐ Behavioural & social sciences ☐ Ecological, evolutionary & environmental sciences

For a reference copy of the document with all sections, see nature.com/documents/nr-reporting-summary-flat.pdf

Life sciences study design

All studies must disclose on these points even when the disclosure is negative.

Sample size	This study comprises a total number of 1472 participants that consisted only of healthy controls.
Data exclusions	Four participants were excluded due to data quality concerns
Replication	We verified reproducibility at three levels. First, the brain age model was trained with five-fold, fifteen-repeat cross-validation and produced consistent age predictions across splits. Second, we applied the trained model to five independent cohorts of tango, music, visual art, gaming expertise and gaming learners spanning EEG, MEG and simulated functional connectivity, and in every cohort we replicated the central result of lower BAGs in creative experts or post-training learners relative to matched controls. Third, we ran sensitivity checks that included age-bias correction, data-quality indexing, domain-specific subsamples and exclusion of the gaming cohort, all of which confirmed the same direction and significance of BAG effects. No analysis contradicted the main findings
Randomization	We have no experimental groups, with the exception of the pre/post training design with videogames, where randomization does not apply.
Blinding	We performed a retrospective re-analysis of fully anonymised, previously published datasets: all raw signals were processed with automated pipelines, and group labels (expert vs non-expert or pre- vs post-training) were applied only at the statistical-testing stage; no subjective assessments or investigator-driven interventions depended on knowing group allocation during data collection or feature extraction.

Reporting for specific materials, systems and methods

We require information from authors about some types of materials, experimental systems and methods used in many studies. Here, indicate whether each material, system or method listed is relevant to your study. If you are not sure if a list item applies to your research, read the appropriate section before selecting a response.

Materials & experimental systems

n/a	Involved in the study	
☒	☐	Antibodies
☒	☐	Eukaryotic cell lines
☒	☐	Palaeontology and archaeology
☒	☐	Animals and other organisms
☒	☐	Clinical data
☒	☐	Dual use research of concern
☒	☐	Plants

Methods

n/a	Involved in the study	
☒	☐	ChIP-seq
☒	☐	Flow cytometry
☒	☐	MRI-based neuroimaging

Plants

Seed stocks	*Report on the source of all seed stocks or other plant material used. If applicable, state the seed stock centre and catalogue number. If plant specimens were collected from the field, describe the collection location, date and sampling procedures.*
Novel plant genotypes	*Describe the methods by which all novel plant genotypes were produced. This includes those generated by transgenic approaches, gene editing, chemical/radiation-based mutagenesis and hybridization. For transgenic lines, describe the transformation method, the number of independent lines analyzed and the generation upon which experiments were performed. For gene-edited lines, describe the editor used, the endogenous sequence targeted for editing, the targeting guide RNA sequence (if applicable) and how the editor was applied.*
Authentication	*Describe any authentication procedures for each seed stock used or novel genotype generated. Describe any experiments used to assess the effect of a mutation and, where applicable, how potential secondary effects (e.g. second site T-DNA insertions, mosiacism, off-target gene editing) were examined.*
