# Supplementary material for: Creative experiences and brain clocks
Source: Nat Commun. 2025 Oct 3;16:8336. doi: 10.1038/s41467-025-64173-9 (PMC12494922; doi:10.1038/s41467-025-64173-9)
Supplement: Supplementary file 3 — Transparent Peer review file [file 41467_2025_64173_MOESM3_ESM.pdf]

nature portfolio

Peer Review File

Creative experiences and brain clocks

Corresponding Author: Dr Agustín Ibáñez

This file contains all reviewer reports in order by version, followed by all author rebuttals in order by version.

Version 0:

Reviewer comments:

Reviewer #1

(Remarks to the Author)
This manuscript provides interesting and compelling evidence regarding the relationship between various experiences and brain aging. The basis of the study, as well as the study design and methodological approach, were well-justified. While I have one major conceptual concern regarding the framing of creative experiences (detailed below), as well as several methodological suggestions, I believe these issues can be addressed through revision.

The major conceptual issue that warrants attention is the framing of video gaming as a creative experience. While some video games can involve creative problem-solving and strategic thinking (e.g., Lubart et al., https://doi.org/10.1037/aca0000547), the authors should more explicitly justify why the specific video game used in this study constitutes a creative experience. The current findings could potentially be interpreted differently - rather than demonstrating unique effects of creative experiences on brain aging, the video gaming results might suggest that any cognitively engaging activity provides similar benefits. This alternative interpretation would align with existing research on cognitive stimulation and brain aging. The authors should address this explicitly by either: (1) providing stronger theoretical and empirical support for categorizing their specific video game as a creative experience, including how 'actions per minute' relates to creativity, or (2) acknowledging that the video gaming condition may serve more as an active control, which would suggest creative activities (visual arts, music) buffer against brain aging similarly to other engaging cognitive activities. This conceptual clarification would strengthen the theoretical contribution of the work.

“Only a few works addressed the impact of creativity, specifically music expertise, on brain structural correlates” — I don’t think this is an accurate statement as numerous studies have investigated how brain structure relates to creativity across domains (e.g. Shi et al., 2017; Zhu et al., 2016). I recommend that the authors rephrase this statement to be specific to the impacts of creativity on structural indices of brain health/age.
Shi, B., Cao, X., Chen, Q. et al. (2017). Different brain structures associated with artistic and scientific creativity: a voxel-based morphometry study. Scientific Reports, 7, 42911. https://doi.org/10.1038/srep42911
Zhu, W., Chen, Q., Tang, C., Cao, G., Hou, Y. & Qiu, J. (2016). Brain structure links everyday creativity to creative achievement, Brain and Cognition, 103, 70-76. https://doi.org/10.1016/j.bandc.2015.09.008.

How exactly was the training data (i.e., the sample of 1240 participants) collected? I understand that the sample of 227 is drawn from various studies, and I assume the same may be true about the sample of 1240 but this does not appear to be explicitly stated in the manuscript. Relatedly, further detail about the recruitment procedures, participant experiences (e.g., study length and tasks involved) and compensation, and exclusion criteria should be added to the manuscript.

“EEG functional connectivity obtained from simulations of DTI structural connectivity” — The authors should clarify this statement. Did the authors mean something like: EEG functional connectivity was estimated based on DTI structural connectivity? A brief explanation and justification of this approach should also be provided in the manuscript.

Line 351 says that 27 participants were recruited for the pre/post learning study, but section 4.3 only refers to 24 participants. Is one of these a typo, or was data collected from some participants but not included in analyses? If certain participants were excluded, the exclusion criteria should be delineated in the manuscript.

It would be helpful to readers if the authors clarified how exactly the video game used in study 2 involved creativity.

There seem to be typos on lines 129, 219-220, 239, and 454, as well as in Figure 2B.

Reviewer #2

(Remarks to the Author)
Summary

This is an excellent piece of work and the authors should be commended for the efforts in collating such an impressive dataset. I have some comments that I hope will further improve the manuscript.

Major points:

1. In general I am confused about the data used to train the brain age model. When I go to the OSF page it is quite disorganized and appears to relate to a different project. How does this project relate to the project on the OSF page? I am also unclear what EEG/MEG paradigm was used in the training/testing dataset. Please clarify and in general improve the description of the data used to train the brain age model. Please also extract the relevant info (or at least provide a readme) from the OSF page and clean this up so that it is clearer what scripts and data were used in this project specifically.
2. My biggest concern with the interpretations would be the effect of confounds related to expertise. It is good that you matched based on education but I am concerned about other socioeconomic and general cognitive (e.g., IQ) indicators that may be correlated with expertise. This concern is mitigated by the pre- post- data but do you have any other socioeconomic or cognitive indicators that you could control for in the cross-sectional analyses?
3. The effects of the video game study seem almost too good to be true: I work in cognitive training and finding meaningful effects with a learning paradigm of this length is unusual. It makes me question the validity of the measure: do you have any behavioral measures (not only from the game itself) that you think could relate to brain age gap that could be used to validate that this measure has real-world implications? I also think while the pre- post- study increases the “causal” inference you can make about creative expertise, it does seem to contradict the theme of the overall article that “expertise” is required if 30 hours of video gaming has a similar effect (-3.1 years compared to -4.1 years in the gaming expert sample) to years and years of training. Maybe you could elaborate on how these findings can be reconciled? Maybe due to effect size inflation on the small samples? It is also unclear why you would not expect the global coupling effects to emerge after 30 hours of training but you do expect to see a reduction in brain age.
4. Given the sample used to train the data probably included more non-experts than experts (experts are rarer in the general population), is it possible that the brain age models were better able to characterize brain age in non-experts? Is there a way you can evaluate this?
5. Please clarify where the samples came from: how were participants recruited and based on what criteria. Also, please clarify if the individual datasets are complete (i.e., were the experts and matched non-experts recruited as part of a specific study or was there a larger number of non-experts that you selected from during matching?). In general, the description of the datasets is insufficient: I would like to know, for example, if these datasets have been used individually in past papers or if they were collected specifically with the goal of collating and performing this analysis.
6. Please describe the matching process in more detail. I know that matching is challenging across multiple domains but some differences exist (non-significance is a low bar for matching success): e.g., age in the dance sample. Please also report normality for age and education to ensure t-tests are appropriate.
7. Having worked with brain age data before, I know that sometimes brain age gaps can be correlated with age, making it somewhat complicated to disentangle this variance. Was this true in your data and did you think about how to control for this?
8. Your brain age model only explains about half of the variance associated with age ($r=.742$, $R^2=55\%$). You cite several papers suggesting that this is in line with other work but the original Cole et al., “brain age predicts mortality” paper (using structural MRI) had an R^2 of 88%. These methods clearly work best if there is a strong correlation between predicted and actual age and while I think yours may be adequate it will produce a lot of noise in brain age gap estimates. I think you can make this clearer as a limitation.

Minor points:

1. I do not think the term “brain health clocks” is appropriate. We do not really know much about brain age as a measure and the extent to which it reflects brain health. I would prefer a more descriptive term (e.g., brain age or brain age gap) that has been more extensively used in the literature to refer to this specific type of method. “Brain clocks” is better but still seems more confusing than just using one term (brain age) throughout.
2. Please be clear when you are talking about brain age gaps: sometimes you use words like “larger” which could mean more positive or more positive/negative. Using directional terms avoids confusion (e.g., accelerated vs. decelerated brain aging). Another example: “Conversely, people with healthier habits such as systematic creative experiences could be hypothesized to have shorter BAGs”. Would you not expect these gaps to be larger but in the negative direction (brain age lower than chronological age)?
3. I do not typically think of occipital regions as particularly age-vulnerable. Can you provide some references to justify this hypothesis? Reading the references you provide they do not seem to suggest this: the main mention of occipital cortex in reference 32 reads as follows: “Regional heterogeneity of synaptic vulnerability in ageing has also been suggested. Similarities in protein expression between young and old occipital cortex synaptic populations in nonhuman primates and humans indicated resistance to age-related damage in this region, whereas the hippocampal synaptic population displayed proteomic heterogeneity on ageing, possibly indicating selective vulnerability of aged hippocampal synapses (Graham et al., 2019). Indeed, resistant (occipital cortex) and vulnerable (hippocampal) brain regions seemed to age differently in nonhuman primates and humans.” This paragraph suggests the occipital cortex is resistant to aging. Reference 34 similarly states: “Subsequently, we modeled cortical thickness changes over lifespan, revealing that fronto-temporo-parietal hubs

were among the brain regions that changed the most, whereas occipital hubs showed a quite spared cortical thickness across ages." Although reference 33 does show differences in occipital regions my general understanding of the literature is that these regions are less vulnerable to aging. It is also surprising that you do not mention the hippocampus or DMN: regions known to be vulnerable to aging.
4. Given brain age can be calculated using different modalities and is most commonly done using structural brain data, I think it would help the reader if you clarified that it was "EEG brain age" or at least "functional brain age" throughout the paper to differentiate from structural approaches.
5. You only talk about age-associations in terms of vulnerability. It might be worth including in the discussion that some age-related regions could be involved in processes that improve with age (e.g., semantic knowledge, emotional well-being) or are compensatory for aging.

Reviewer #3

(Remarks to the Author)
The manuscript titled "Creative Experiences and Brain Health Clocks" aims to enhance our understanding of how various creative experiences influence the functional connectivity characteristics of brain networks and assesses the implications of these changes on brain aging. The findings suggest that creative expertise may slow down brain aging, as reflected by functional connectivity alterations. Furthermore, by employing a longitudinal design, the study demonstrates that changes associated with slowed aging are also evident when individuals engage in creative experiences, specifically through playing video games.

While I find the results intriguing and relevant to the broad readership of Nature Communications, with potential transformative implications for promoting brain health through everyday activities, I do not believe that the manuscript, in its current form, provides sufficiently robust evidence to support its claims. However, I believe that most of my concerns can be resolved with substantial but feasible revisions.

Below, I outline the major limitations that form the basis of my decision.

Major Points:

1. Lack of adequate information in the introduction:
- The final paragraph presents multiple hypotheses that are not well justified. These read more like a foreshadowing of the results rather than a rational anticipation of testable hypotheses. Improved integration with prior literature could strengthen this section. For example, it is unclear why the study hypothesizes that creative experiences would be associated with delayed brain aging "especially in expertise study" but "to a lesser extent in the pre/post-learning study." Such hypotheses require clearer justification or should be revised.
- The manuscript posits that since "frontoparietal and occipital hubs" are most vulnerable to aging, creative experiences would primarily impact these regions. While frontoparietal regions are consistently reported as aging-sensitive, the involvement of occipital regions is less well-supported. Even within the cited literature, other vulnerable regions—such as centrotemporal areas—are mentioned, which are left out by the current article. Additionally, multiple studies indicate that aging affects regions within the default mode network (DMN) and subcortical structures, yet these are overlooked in both the introduction and discussion.
- The introduction claims that the "combination of graph theory and biophysical modeling" with brain age gap (BAG) measures can provide insight into brain aging mechanisms. However, this claim appears without context. The manuscript should provide appropriate references explaining why these specific methods are particularly informative.
- Brain age gap estimates can be derived from different imaging modalities (e.g., structural, volumetric, connectivity, or functional measures). The introduction appears to conflate these measures as if they are directly comparable, but this assumption is not well-established. I recommend avoiding interpretations of structural brain age findings to justify functional brain aging analyses unless explicitly supported by prior work.

2. Methodological Concerns: Overinterpretation of Multi-Modal Data Integration (This is my most significant methodological concern.)
- The study combines three different measures of functional connectivity across datasets: [1] EEG-derived functional connectivity (FC) [2] MEG-derived FC [3] Simulated EEG from diffusion tensor imaging (DTI). The manuscript argues that "EEG and MEG capture similar neural and can be integrated into a single pipeline." However, this is a problematic assumption. EEG and MEG have fundamental differences (see Coquelet et al., 2020, NeuroImage), and even within similar areas, correlations between EEG- and MEG-derived connectivity measures are modest (below 0.5). The cited justification for integration (Muthuraman et al., 2015) supports using concurrent EEG and MEG recordings for robust signal capture but does not endorse combining EEG and MEG connectivity data from different subjects in a unified comparison. The inclusion of simulated EEG from DTI is even more concerning. While EEG simulations from structural data provide insights into large-scale dynamics, they remain an approximation of real neural activity and cannot be assumed to be equivalent to either EEG or MEG data.
- Suggested solution to resolve this critical issue: Instead of pooling datasets to increase sample size, I strongly recommend performing separate analyses for each dataset (dancers, musicians, visual artists, and gamers). If similar spatial patterns and age-related effects emerge across independent tests, this would provide strong cross-modality replication without introducing confounds related to data integration across modalities.

3. Confounding Variables:

- Several confounds could influence the reported effects: Individuals engaged in creative activities may differ in socioeconomic status or exposure to adversity, both of which are known to influence BAG (see Cohen et al., 2023, Dev Psychol; Rakesh et al., 2023, Trends Cogn Sci). If such variables are available, I recommend including them in sensitivity analyses or otherwise acknowledging them as limitations.
- In the longitudinal study, post-learning scans show lower BAGs than pre-learning scans which is interpreted as the effect of the creative experience. However, this could be partially driven by increased familiarity with data collection procedures rather than true neurobiological change. If longitudinal data are available from individuals who did not undergo learning interventions, their trajectories should be examined to control for this potential confound. Otherwise, this should at the very least be indicated as a limitation due to the inavailability of such data.

4. Statistical Issues in Spatial Comparisons:
- The manuscript correlates different spatial maps (e.g., Fig. 4A, comparing age vulnerability maps with nodal strength differences) using Pearson's correlation p-values. This method is known to inflate false-positive rates due to spatial autocorrelation (see Markello et al., 2021, NeuroImage). More robust statistical approaches have previously been proposed to correct this issue, such as: [1] Spin tests (Alexander-Bloch et al., 2018, NeuroImage), [2] Moran spectral randomization (Wagner et al., 2015), and [3] Generative models accounting for spatial autocorrelation (Burt et al., 2020, NeuroImage). These methods are implemented in software such as NeuroMaps (Markello et al., 2022, Nat Methods) and can easily be applied instead of a simple Pearson's correlation. I strongly recommend re-evaluating spatial correlations using these techniques. Please note that this comment concerns all spatial comparisons (e.g., including the comparisons with Neurosynth maps).

5. Justification for Biophysical Modeling:
- The role of biophysical modeling and global coupling in this study remains unclear. Since these results are not reflected in the longitudinal analyses, their added value is questionable. Many connectivity metrics are interrelated, and it is unclear whether global coupling provides meaningful additional insights beyond traditional graph-theoretical measures such as global efficiency. I think it might help the overall readability of the manuscript if this information is left out. Otherwise, the article should provide additional discussion on the specific knowledge that was revealed by the additional step of biophysical modeling.

6. Issues with Data Visualization:
- The cortical projections of spatial brain maps lack clear delineations of the AAL atlas regions and appear to imply a high-resolution spatial map, which is not what is being analyzed. I recommend revising the visualizations to more accurately reflect the resolution of the data.

7. Important connections predicting age:
- Figure 2.b presents a comparison of connections that were "most important" for predicting age. I could not find any explanation of how this was measured in the manuscript. Could the authors clarify?

8. Choice of colormaps:
- Figure 4.a uses an unconventional colormap with a sharp transition from cyan to pink somewhere arbitrary in the value range, which is not good practice. I would suggest changing the colormaps to perceptually uniform alternatives (e.g., viridis/plasma) to avoid producing inaccurate perceptions. Moreover, for ranges that include both positive and negative ranges, I would suggest using a symmetric diverging colormap centered at zero to distinguish positive vs. negative effects.

Minor points:

- The manuscript had several typos/errors. Here are a few that I spotted (but this is not a complete list; I would recommend checking the whole document to ensure such errors are all fixed):
> line 141: shorter BAGs -> lower BAGs
> line 155: groups of experts and non-experts tango dancers -> groups of expert and non-expert tango dancers
> line 193: withing -> within
> line 228: The -> Then
> Line 273: undelaying -> underlying?

- In the first paragraph of the results section, I recommend including the age range of the participants in the dataset. I know this is later mentioned in the methods and supplementary material, but adding that information to the results could improve the overall readability.

- In Figure 2.a the BAG predictions are shown in a scatter plot against chronological age. I recommend including a supplementary figure that shows the predicted age (before accounting for the regression to the mean effect) to better reflect the actual model accuracy. It should be noted that even a random prediction with small variance may lead to low MAE and high correlations after accounting for RTM, and it is hence highly recommended to also report and visualize the accuracy before accounting for RTM to better portray model performance.

- Several previous works in the intersection of graph theory metrics and brain aging have reported changes in local efficiency, seggregation, integration, and global efficiency to be linked to aging. I'd recommend citing a few of such previous works to further link with the existing literature.

- The sentence in lines 257 to 259 was unclear to me. Did the authors mean to say global coupling instead of local

efficiency? I don't see how the two are linked.

- Figure 1.D seems to suggest that both EEG and MEG data were available for all domains, which is not the case. (it could maybe say EEG "or" MEG instead of EEG "&" MEG)

- In Figure 4.b, for the significant cognitive processes (reported in the middle), it would also be good to include the cortical map of each cognitive process to help interpretability of the figure for the reader.

Reviewer #4

(Remarks to the Author)
I co-reviewed this manuscript with one of the reviewers who provided the listed reports. This is part of the Nature Communications initiative to facilitate training in peer review and to provide appropriate recognition for Early Career Researchers who co-review manuscripts.

Version 1:

Reviewer comments:

Reviewer #1

(Remarks to the Author)
The revised manuscript is much improved and my prior comments were all appropriately addressed. I commend the authors for their efforts. I do not have any additional concerns about the manuscript. I believe this manuscript provides a high quality contribution to the literature regarding creative experiences and brain age.

(Remarks on code availability)
N/A

Reviewer #2

(Remarks to the Author)
The authors have overall made a commendable effort to address the reviewers' comments. There are just a couple of situations in which I feel their response either side-steps a reviewer comment or brings up further questions and I would like to see these clarified before publication:
1. Response to 1.2. I don't think the response really deals with the issue the reviewer is raising. Removing the gaming group does not remove the fact that these results exist and still point to the alternative hypothesis that cognitively engaging activities are important rather than creativity, necessarily. I think that the active control group does help address this question but also raises its own issues (see point 2).
2. The active control group. I am confused by this: why was this group not originally included? Was the group recruited and analyzed especially for the rebuttal? The timing of this would seem unlikely but I suppose it is possible. In any case, a better explanation of the games and justification for why this game is less creative is needed: really the idea that creativity in itself is what is important rests on this very small (N=12) control group and this should be included as a major limitation with acknowledgement that cognitive stimulation is an alternative explanation. Additionally, given the inclusion of a control group it is now possible to use statistical tests that are recommended for assessing change vs. a control group (controlling for baseline differences). Showing change in each group in isolation is not typically considered robust for this kind of design. It also seems important to me to better clarify exactly what cognitive process is being measured via the "blind paradigm" (should this be "blink"?) and whether you think this should relate to the Brain Age Gaps and why (also do they relate?).

3. As a separate point I tried to clone and run the code but I am having trouble with the "plot_violins" module (error: there is no module named 'plot_violins'). Is this a script you have written that is not available on the GitHub or it is from a larger module that is included in the dependencies (I have installed all dependencies successfully)? This might be my mistake but please elaborate in your readme to make it easier for others to run this code.

(Remarks on code availability)
I am not an expert at coding and have not used all of the packages provided here. A brief examination suggests the code exists and the readme is sufficient if not amazing. Similar with the commenting: not the best I've seen but not terrible. I am having an issue running the code due to the "plot_violins" module and have asked the authors to further clarify where this is coming from.

Reviewer #3

(Remarks to the Author)
I appreciate the authors' thoughtful and thorough revisions. They have addressed all of the concerns I previously raised. In particular, I commend the rigorous effort put into the additional sensitivity analyses, which clearly demonstrate the robustness of the findings.

I believe the manuscript is now suitable for publication and have no further comments or requests.

(Remarks on code availability)
I have reviewed the Git repository prepared by the authors. While I have not executed the code, the repository appears to be well-organized and includes adequate documentation and supporting data. In my view, it provides sufficient detail to allow for future replication and reuse.

Reviewer #4

(Remarks to the Author)
I co-reviewed this manuscript with one of the reviewers who provided the listed reports. This is part of the Nature Communications initiative to facilitate training in peer review and to provide appropriate recognition for Early Career Researchers who co-review manuscripts.

(Remarks on code availability)

Reviewer #5

(Remarks to the Author)
This manuscript presents an innovative and ambitious study linking creative expertise and learning to delayed brain aging, as measured by EEG/MEG-based brain clocks. While I have a generally positive impression of the work, I have limited my assessment to the methodological aspects, particularly those involving M/EEG source reconstruction and connectivity analysis.

The authors use standard and appropriate preprocessing and source localization techniques for both EEG and MEG. The use of sLORETA and beamforming represents a robust and widely accepted approach to solving the inverse problem, providing a valid pipeline to estimate neural activity at the source level.

However, my main concerns relate to how this source-level activity is used to infer functional connectivity (FC) and, by extension, brain age gaps (BAGs). Several methodological choices which, although not necessarily incorrect, lack justification and their limitations are not acknowledged. As these choices do not invalidate the present results, I recommend publication of this manuscript, once all these aspects are properly addressed:

1- Choice of Features and Frequency Range. There are many features derived from EEG/MEG data that correlate with brain age (e.g., power spectral density, entropy, kurtosis, etc.; see Engemann et al., NeuroImage, 2022). The authors' decision to rely exclusively on functional connectivity in the 8–40 Hz band should be justified. Why was this specific frequency range chosen, and why were other features or narrower bands not considered?

2- Use of Pearson Correlation for Functional Connectivity. My second concern relates to the use of Pearson correlation as the metric for functional connectivity. While it can provide a rough approximation of connectivity, it has well-known limitations that are not acknowledged in the manuscript. The most significant issue is volume conduction: high correlations between brain regions may simply reflect shared signal sources rather than genuine functional interactions. This can lead to inflated or misleading connectivity estimates. The choice of Pearson correlation over more robust alternatives—such as imaginary coherence, phase-lag index, or orthogonalized connectivity measures—is not justified. A critical discussion of this decision, and its implications for the interpretation of the results, would be necessary. For a review of functional connectivity maps in EEG and MEG, see van Diessen et al., Clinical Neurophysiology, 2015.

3- Sensor Density and Source Reconstruction Accuracy. The accuracy of source-level functional connectivity estimates is highly dependent on the number of recording sensors. Estimating activity from 78 cortical regions using only 32 EEG channels may lead to substantial spatial leakage and inflated correlations due to low spatial resolution. Have the authors assessed whether FC estimates — or BAGs — are systematically higher or more variable in datasets recorded with fewer sensors (e.g., 32 vs. 264 channels)? This is especially relevant given the multi-site nature of the dataset.

Minor comments:

The manuscript should include the number of participants contributed by each center or dataset, ideally in the main table. This would help assess the potential for site-specific effects or sampling bias.

Typo in line 460: "exclusion criteria included normal or corrected-to-normal vision..." I do not think these are exclusion criteria.

(Remarks on code availability)

Version 2:

Reviewer comments:

Reviewer #2

(Remarks to the Author)
Thank you for further addressing my comments. Although the analysis chosen for comparing active and control groups is unusual I do not think it requires an additional round of revisions by itself. I think I have requested sufficient revisions at this point and the authors have done a commendable job of addressing them.

I will note what I think is a typo: "These effects were specific to StarCraft II itself, as we did not find any differences in the active control of the pre/post design (ΔBAGs = 0.057, t = 0.979, p = 0.0274, D = 0.0092, FDR-corrected, Figure S11 in Supplementary Materials)." this p-value seems unlikely for the t-statistic and effect sizes shown and appears to be significant... I am wondering if it is a typo.

(Remarks on code availability)
The code still doesn't run for me. Thank you to the authors for adding plot_violins.py: the script successfully gets past this point now but fails at importing from surfplot. Maybe another custom plotting script that needs to be added?

Reviewer #5

(Remarks to the Author)
I apprecaite the thorough and thoughtful responses to all my comments. The additional analyses and clarifications on the choices and linimations have fully addressed my methodological concerns. I have no further comments, and I support the publication of this manuscript in its current form.

(Remarks on code availability)

Open Access This Peer Review File is licensed under a Creative Commons Attribution 4.0 International License, which permits use, sharing, adaptation, distribution and reproduction in any medium or format, as long as you give appropriate credit to the original author(s) and the source, provide a link to the Creative Commons license, and indicate if changes were made.
In cases where reviewers are anonymous, credit should be given to 'Anonymous Referee' and the source.
The images or other third party material in this Peer Review File are included in the article’s Creative Commons license, unless indicated otherwise in a credit line to the material. If material is not included in the article’s Creative Commons license and your intended use is not permitted by statutory regulation or exceeds the permitted use, you will need to obtain permission directly from the copyright holder.
To view a copy of this license, visit https://creativecommons.org/licenses/by/4.0/

RESPONSE TO REVIEWERS

The following point-by-point responses in red have been prepared to address the Reviewers' comments. All additions and modifications made in the revised manuscript are highlighted in yellow. All new references have been incorporated into the References section.

Reviewer #1:

Remarks to the Author:

Comment 1.1: This manuscript provides interesting and compelling evidence regarding the relationship between various experiences and brain aging. The basis of the study, as well as the study design and methodological approach, were well-justified. While I have one major conceptual concern regarding the framing of creative experiences (detailed below), as well as several methodological suggestions, I believe these issues can be addressed through revision.

Response 1.1: Thank you for the positive comments about our manuscript. We addressed all your comments in the responses below, including the framing of video gaming as a creative experience.

Comment 1.2: The major conceptual issue that warrants attention is the framing of video gaming as a creative experience. While some video games can involve creative problem-solving and strategic thinking (e.g., Lubart et al., https://doi.org/10.1037/aca0000547), the authors should more explicitly justify why the specific video game used in this study constitutes a creative experience. The current findings could potentially be interpreted differently - rather than demonstrating unique effects of creative experiences on brain aging, the video gaming results might suggest that any cognitively engaging activity provides similar benefits. This alternative interpretation would align with existing research on cognitive stimulation and brain aging. The authors should address this explicitly by either: (1) providing stronger theoretical and empirical support for categorizing their specific video game as a creative experience, including how 'actions per minute' relates to creativity, or (2) acknowledging that the video gaming condition may serve more as an active control, which would suggest creative activities (visual arts, musicians) buffer against brain aging similarly to other engaging cognitive activities. This conceptual clarification would strengthen the theoretical contribution of the work.

Response 1.2: Thanks for raising this issue. We now defined creativity early in the introduction and have clarified how video games involve creativity and provided additional results excluding gaming (**Figure S7** in **Supplementary Material**):

Introduction paragraph 1:

> Creativity is defined here as the ability to produce ideas or solutions that are both novel and effective using one's imagination[1]. Creativity traditionally includes arts, but video games can incorporate creative elements[2,3]. For instance, strategy video games, such as StarCraft II, materialized creativity in unique tactics, adaptive problem-solving, and personalized playstyles[4].

Discussion paragraph 5:

> Gaming can be considered a type of creative experience[2,3,5], even though it is not traditionally classified as such. For this reason, we repeated the analyses in the expertise study, excluding the gaming groups. The results remained unchanged (**Figure S7** in **Supplementary Materials**).

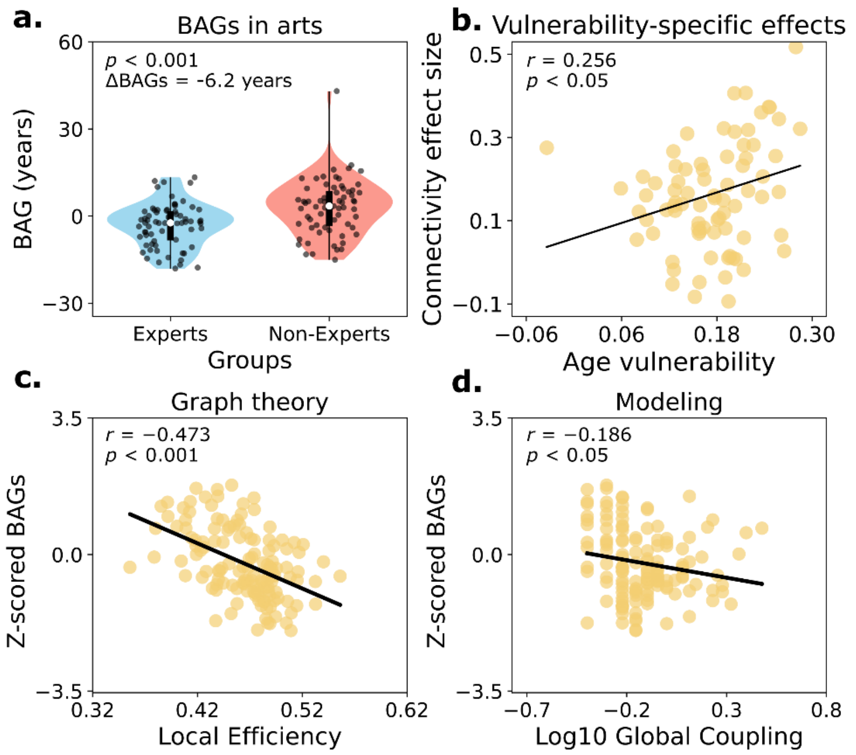

Figure S7. Validation of analyses using only artistic creativity datasets: tango dancers, musicians, and visual artists. **a.** BAGs between experts and non-experts (ΔBAGs = -6.2, t = -4.163, p = 1.128x10^{-4}, D = -0.72, FDR-corrected). Correlations between **b.** vulnerability-specific effects and brain connectivity (r = 0.256, p = 0.016, Cohen's f^2 = 0.070, spin test, FDR-corrected); **c.** local efficiency (segregation) and BAGs (r = -0.473, p = 3.46x10^{-8}, Cohen's f^2 = 0.288, FDR-corrected); **d.** Global coupling and BAGs (r = -0.186, p = 0.032, Cohen's f^2 = 0.036, FDR-corrected). Points represent participants.

Comment 1.3: "Only a few works addressed the impact of creativity, specifically music expertise, on brain structural correlates" — I don't think this is an accurate statement as numerous studies have investigated how brain structure relates to creativity across domains (e.g. Shi et al., 2017; Zhu et al., 2016). I recommend that the authors rephrase this statement to be specific to the impacts of creativity on structural indices of brain health/age.
Shi, B., Cao, X., Chen, Q. et al. (2017). Different brain structures associated with artistic and scientific creativity: a voxel-based morphometry study. Scientific Reports, 7, 42911. https://doi.org/10.1038/srep42911
Zhu, W., Chen, Q., Tang, C., Cao, G., Hou, Y. & Qiu, J. (2016). Brain structure links everyday creativity to creative achievement, Brain and Cognition, 103, 70-76. https://doi.org/10.1016/j.bandc.2015.09.008.

Response 1.3: Thank you for these suggestions. We have revised the statement about the novelty and clarified as follows:

> Multiple works have characterized the brain's structural and functional associations with creativity and artistic experiences[6,7]. However, these studies have not linked creative experiences with delayed brain aging. Only a few works addressed the impact of creativity on BAGs[8,9] but using a single domain (music expertise) and addressing structural brain age, which is not sensitive to functional changes[10]. Furthermore, no previous work has provided mechanistic explanations underlying changes in brain age. Studies have also failed to assess diverse forms of creative experience or to use robust methodological designs. These would include appropriate control and matching procedures, as well as testing across multiple experiments when analyzing the effects of art on brain health[11].

Comment 1.4: How exactly was the training data (i.e., the sample of 1240 participants) collected? I understand that the sample of 227 is drawn from various studies, and I assume the same may be true about the sample of 1240 but this does not appear to be explicitly stated in the manuscript. Relatedly, further detail about the

recruitment procedures, participant experiences (e.g., study length and tasks involved) and compensation, and exclusion criteria should be added to the manuscript.

Response 1.4: Thank you for raising this concern. In the revised version of the manuscript, we have provided additional details about the training sample as follows:

Methods: Participants and demographics

> We used data from 1,240 healthy controls (HCs) from the EuroLaD EEG consortium[12], to train the SVM models, including participants from global settings. Specifically, 724 HCs were from the global north (Turkey, Greece, Italy, the United Kingdom, and Ireland) (age range 17–91 years, mean age 45.7 ± 22.6 years, 48.8% female/male distribution), while 516 were from the global south (Cuba, Colombia, Brazil, Argentina, and Chile) (age range 18–89 years, mean age 46.4 ± 18.5 years, 50% female/male distribution). Inclusion criteria required normal cognitive function and no history of disease. Study protocols were approved by each contributing institution's Institutional Review Board (IRB), and all participants provided informed consent following the Declaration of Helsinki. The complete demographics are presented in **Table 1.**

We also added more information about the creativity groups:

Methods: Participants and demographics

> As an out-of-sample validation, we incorporated 232 participants from different independent studies about creativity expertise (study 1) and pre/post-learning (study 2, **Figure 1C**).

Methods: Expertise criteria: Tango dancers

> Expert and beginner tango dancers were recruited from three tango schools in Buenos Aires — DNI, Flor de Milonga, and Divino Estudio del Abasto Tango school. [...] Exclusion criteria included had normal or corrected-to-normal vision, reported no past neurological or psychiatric history, and were right-handed, verified by the Edinburgh Inventory.

Methods: Expertise criteria: Musicians

> Data were obtained from the OMEGA repository. Musicians were selected based on self-reported experience of playing a musical instrument for five or more years.

Methods: Expertise criteria: Visual artists

> Recruitment occurred through social media, posters in locations related to the topic (e.g., universities, art schools, and art institutions), and word of mouth. After initial email contact, candidates were screened for inclusion and exclusion criteria and asked about their artistic background. Suitable participants were then invited to schedule an EEG session, during which a more detailed questionnaire on their art practice and interests was completed. [...] Exclusion criteria included psychiatric, neurological, or cardiac conditions, and hairstyles or accessories (e.g., dreadlocks) that could impair EEG data acquisition. [...] Participants were informed about the data collected and signed informed consent before the session. Compensation was provided, although the specific form or amount is not mentioned in the article.

Methods: Expertise criteria: Gaming

Participants were recruited using an online questionnaire administered via the GEX platform (GEX Immergo, Funds Auxilium Sp. z o.o), which gathered demographic information, education status, and detailed data on video game habits. As part of the questionnaire, participants provided their Battle.net ID, allowing verification of their StarCraft II league ranking and recent gameplay activity. [...] All participants were right-handed males, had no history of neurological illness or psychoactive substance use, and were matched on undergraduate-level education. Working memory capacity was assessed using a modified online version of the operation span (OSPAN) task, with participants required to maintain at least 85% accuracy on the math component to be included. Two participants were excluded due to poor MRI data quality. All participants gave written informed consent and received monetary compensation for their participation.

Methods: Pre/post-learning design

Participants were initially recruited online via a covert questionnaire. [...] All participants reported normal or corrected vision, normal hearing, and were right-handed. Exclusion criteria included any history of neurological or psychiatric disorders, head injuries, surgeries, brain tumors, current medication use, or more than five hours of video gaming per week in the prior six months, especially RTS or FPS games. [...] Only participants who completed both sessions and met training requirements were included. Each eligible participant received compensation of approximately 184 USD. […] EEG data were collected during two lab sessions lasting up to two hours each, which included instructions, electrode setup, and an attentional blink task. Only those who completed both sessions and met the training requirements were included in the final analysis. The attentional blink task involved rapid serial visual presentation (RSVP) of letters at the center of the screen. Participants were instructed to detect and report two target letters (T1 and T2) appearing in the stream, with T2 occurring shortly after T1 to measure the transient lapse in attention characteristic of the attentional blink effect. T1 corresponded to a green capital letter (vowel or consonant), and T2 to a black "X" presented at lag 1, 2, or 7 after T1. Participants answered two yes/no questions: whether a vowel (T1) and/or an "X" (T2) appeared. From the test, we reported the T1 and T2 reaction times and accuracy.

Comment 1.5: "EEG functional connectivity obtained from simulations of DTI structural connectivity" — The authors should clarify this statement. Did the authors mean something like: EEG functional connectivity was estimated based on DTI structural connectivity? A brief explanation and justification of this approach should also be provided in the manuscript.

Response 1.5: Thanks for this suggestion. We now clarified in the new manuscript how and why we estimated FC from DTI for the gaming expertise design.

Methods: Expertise criteria: Gaming

As our brain clock models were trained from EEG FC, and this data was not available for participants, we estimated EEG FC from DTI structural connectivity using whole-brain modeling[13,14]. Previous simulated FC from empirical DTI[15] successfully revealed connectivity differences between players and non-players[14]. We also reported the structural differences between groups in **Supplementary Materials Figure S6**.

Comment 1.6: Line 351 says that 27 participants were recruited for the pre/post learning study, but section 4.3 only refers to 24 participants. Is one of these a typo, or was data collected from some participants but not included in analyses? If certain participants were excluded, the exclusion criteria should be delineated in the

manuscript.

Response 1.6: Thank you for highlighting this issue. It was a typo that has been now fixed (N = 24 for the learning design).

Comment 1.7: It would be helpful to readers if the authors clarified how exactly the video game used in study 2 involved creativity.

Response 1.7: Thank you for this comment that we addressed in **response 1.2**

Comment 1.8: There seem to be typos on lines 129, 219-220, 239, and 454, as well as in Figure 2B.

Response 1.8: All these typos have been corrected in the new manuscript.

Reviewer #2:

Remarks to the Author:

Comment 2.1: Summary. This is an excellent piece of work and the authors should be commended for the efforts in collating such an impressive dataset. I have some comments that I hope will further improve the manuscript.

Response 2.1: Thank you for your positive feedback. We have addressed your concerns point by point below.

Comment 2.2: In general I am confused about the data used to train the brain age model. When I go to the OSF page it is quite disorganized and appears to relate to a different project. How does this project relate to the project on the OSF page? I am also unclear what EEG/MEG paradigm was used in the training/testing dataset. Please clarify and in general improve the description of the data used to train the brain age model. Please also extract the relevant info (or at least provide a readme) from the OSF page and clean this up so that it is clearer what scripts and data were used in this project specifically.

Response 2.2: Thanks, we now clarified properly the data and code availability in the new manuscript. We provide FC matrices and data to reproduce our main results.

Data availability:

> FC data and basic demographics can be found at https://github.com/carlosmig/Creativity_Brain_Clocks. The raw data, as well as the FC matrices and demographic for the music expertise design, are available from the corresponding author upon reasonable request.

Code availability:

> Graph analyses were performed using the Brain Connectivity Toolbox for Python (https://github.com/fiuneuro/brainconn)[16]. *Neurosynth* association maps were generated with the NiMARE Toolbox in Python (https://nimare.readthedocs.io/en/latest/index.html)[17]. Full codes to reproduce the main results can be found at https://github.com/carlosmig/Creativity_Brain_Clocks. The linearized Hopf model can be found at https://github.com/adrianponce/Linear-Hopf-model[13]. From replays, StarCraft II telematic data were obtained using sc2reader (https://github.com/ggtracker/sc2reader) and PACanalyzer (https://github.com/Reithan/PACAnalyzer). Brain plots were made using BrainNet Viewer for MATLAB (https://www.nitrc.org/projects/bnv)[18] and the

Python surfplot library (https://pypi.org/project/surfplot/)[19]. The spin test analyses[20] were conducted using the BrainSMASH Python library (https://brainsmash.readthedocs.io/)[21].

Comment 2.3: My biggest concern with the interpretations would be the effect of confounds related to expertise. It is good that you matched based on education but I am concerned about other socioeconomic and general cognitive (e.g., IQ) indicators that may be correlated with expertise. This concern is mitigated by the pre- post-data but do you have any other socioeconomic or cognitive indicators that you could control for in the cross-sectional analyses?

Response 2.3: Thank you for raising these concerns. We have now clarified all the available controls in each experiment. The following changes have been implemented in the manuscript:

Methods: Participants and demographics

> Both expert and non-expert participants were age-, sex-, education-, and geography-matched (**Table 1**). Participants in the gaming group were also matched by working memory capacity (**Table S1 in Supplementary Materials)**. Participants in the dancing group were matched by abstraction capacity, working memory, attention, verbal inhibitory control, and verbal working memory **(Table S5 in Supplementary Materials).**

Table S1. Participants' full demographics and experience with video games for the gaming expertise design.

Variable	Expert (N = 31 males)	Non-experts (N = 31 males)	*t* values	*p* values
Age (years)	24.7 (4.27)	24.4 (3.00)	0.320	0.750
Duration of education (years)	15.55 (2.77)	16.10 (2.95)	-0.757	0.452
OSPAN score (WMC)	51.77 (12.73)	51.71 (13.19)	0.018	0.986
Total number of fibers	10,764 (2,567.80)	10,131 (2,445.32)	0.994	0.324
**Gaming experience**				
Game experience	22.74 (11.78)	2.39 (2.28)	9.443	0.000
StarCraft II experience	18.23 (10.01)	0 (0.00)	10.140	0.000
Real-time strategy	16.06 (9.91)	0.05 (0.15)	8.994	0.000
First-person shooter	1.02 (2.15)	0.27 (0.60)	1.871	0.070
Platform	0 (0.00)	0.06 (0.21)	-1.591	0.122
Fighting	0.16 (0.57)	0 (0.00)	1.563	0.129
Turn-based strategy	1 (1.77)	0.35 (0.70)	1.901	0.065
Sports	0 (0.00)	0.65 (1.42)	-2.549	0.016
Role-play	1.60 (2.08)	0.19 (0.46)	3.685	0.001
Racing	0.13 (0.29)	0.27 (0.55)	-1.254	0.216
Logic	0.66 (1.32)	0.37 (0.66)	1.094	0.280
Multiplayer online battle arena	1.44 (2.99)	0.13 (0.39)	2.419	0.022
Adventure	0.68 (1.88)	0.03 (0.12)	1.921	0.064

Mean and standard deviation (SD in parentheses) represent the values. The term “Game experience” refers to the average number of hours per week spent playing video games in the last 6 months. Similarly, “StarCraft II experience” indicates the average number of weekly hours spent specifically on playing the StarCraft II game

over the past 6 months. OSPAN score was used to assess the working memory capacity (WMC). The table was adapted from the original publication[15], and *t* values were calculated using the summary statistics provided there.

Table S5. Participants' cognitive and behavioral assessments for the dancing expertise design.

Variable	Experts (N = 23)	Non-Experts (N = 23)	t values	p values
Abstraction Capacity (Proverb interpretation)	2.83 (0.28)	2.79 (0.43)	0.406	0.6862
Backward Digit Span	4.38 (1.13)	4.31 (1.11)	0.209	0.8354
Go-No-Go	2.96 (0.20)	2.93 (0.26)	0.43	0.669
Verbal Inhibitory Control	5.29 (0.86)	5.62 (0.82)	-1.417	0.1629
Verbal Working Memory	1.67 (0.64)	1.97 (0.94)	-1.369	0.1772

Mean and standard deviation (SD in parentheses) represent the values. Differences between groups were assessed using t-Student tests.

Comment 2.4: The effects of the video game study seem almost too good to be true: I work in cognitive training and finding meaningful effects with a learning paradigm of this length is unusual. It makes me question the validity of the measure: do you have any behavioral measures (not only from the game itself) that you think could relate to brain age gap that could be used to validate that this measure has real-world implications? I also think while the pre- post- study increases the "causal" inference you can make about creative expertise, it does seem to contradict the theme of the overall article that "expertise" is required if 30 hours of video gaming has a similar effect (-3.1 years compared to -4.1 years in the gaming expert sample) to years and years of training. Maybe you could elaborate on how these findings can be reconciled? Maybe due to effect size inflation on the small samples? It is also unclear why you would not expect the global coupling effects to emerge after 30 hours of training, but you do expect to see a reduction in brain age.

Response 2.4: Thanks for raising this issue. We have now performed additional analyses including a) an active control group for the learning design, and b) the reaction times and accuracy of the blink attentional task. We found differences in both the level of BAGs and cognition (improved reaction times and accuracy) in the experimental group (StarCraft) but not with active control. Similar effects in gaming have been reported by Goldin et al. (2014)[22] demonstrating that short training can improve specific executive function and performance. We have made the next changes in the manuscript.

Results: Higher levels of creative experiences are associated with delayed brain age.

> In the pre/post-learning study, lower BAGs were observed in the post-learning condition compared to the pre-learning one (ΔBAGs = -3.05, t = -2.863, p = 0.034, D = -0.46, FDR-corrected, **Figure 2E**). These effects were specific to StarCraft II itself, as we did not find any differences in the active control pre/post design (ΔBAGs = 0.056, t = 0.027, p = 0.979, D = 0.01, **Figure S11** in Supplementary Materials).

Results: Degree of expertise and performance modulate the brain age gaps

> Using the blind paradigm task outcomes, we then analyzed how the effects of pre/post learning can be translated to generalized performance. Results showed significant improvements after training: participants responded faster to the first question (T1; t = -2.996, p = 0.0258, D = -0.61, FDR-corrected),

and exhibited higher accuracy on the second question (T2; t = 5.958, p = 3.6 x 10^{-4}, D = 1.22, FDR-corrected). We found no effects in the active control group (results in **Supplementary Materials Table S6**).

New Figure 2:

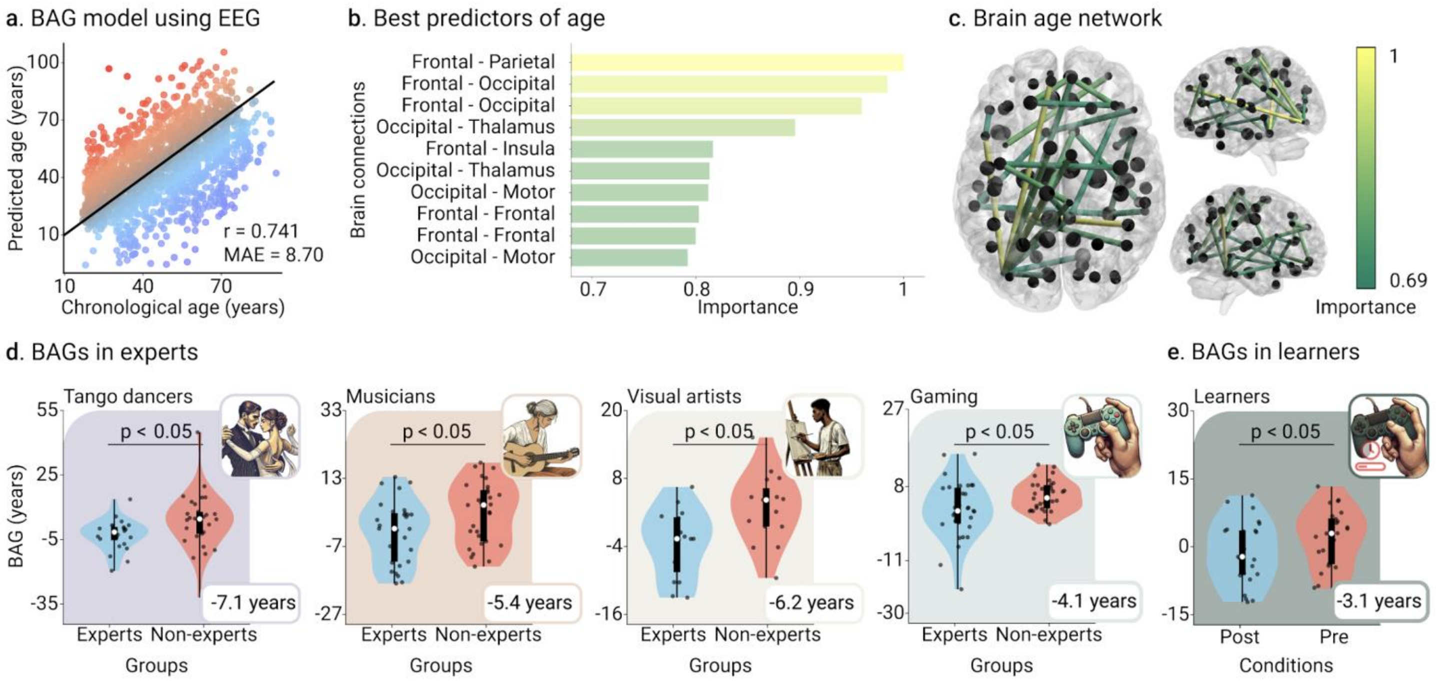

Figure 2. Brain clock model and brain age gaps (BAGs). **a.** The model's performance was assessed by computing the Pearson correlation and the mean absolute error (MAE) between the predicted age and the real chronological age of participants. **b.** Most important (informative) brain connections for predicting age. Top connections reflect the highest absolute SVR weights, indicating their importance in age prediction. **c.** The brain age network comprises the set of most informative connections; the thickness of the edges represents the features' importance of connections for predicting age using SVMs. **d.** BAGs in the expertise study, i.e., tango dancers, musicians, visual arts, and gaming. **e.** BAGs in the pre/post-learning study. Points correspond to participants.

New Figure S11 in Supplementary Materials:

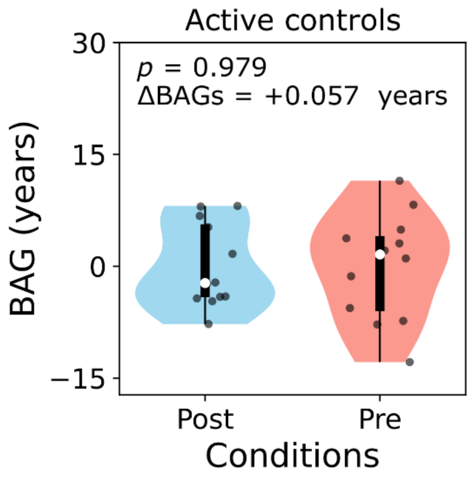

Figure S11. BAGs for the pre/post-learning design active control group. We compared the BAGs post- and pre-training with Hearthstone. We found no BAG differences between the two conditions (ΔBAGs = 0.057, *t* = 0.979, *p* = 0.0274, D = 0.0092, FDR-corrected). Box plots were built using the median, 1st and 3rd quartiles, and the maximum and minimum values of the distributions.

Table S6. Blind paradigm task outcomes for the pre/post-learning design.

Variable	Group	Pre	Post	t values	p values	Cohen's D	P (FDR-corrected)
T1 Acc	Active control	219.3 (14.2)	222.6 (17.6)	1.2890	0.2238	0.3721	0.2558
T1 RT (msec)	Active control	658.1 (278.6)	560.8 (254.7)	-1.4452	0.1763	-0.4172	0.2350
T2 Acc	Active control	139.0 (28.1)	148.8 (23.3)	1.4549	0.1736	0.4200	0.2350
T2 RT (msec)	Active control	376.2 (130.7)	296.3 (159.0)	-2.1853	0.0514	-0.6308	0.1370
T1 Acc	Learner s	204.7 (41.1)	211.4 (32.4)	0.7950	0.4347	0.1623	0.4347
T1 RT (msec)	Learner s	857.0 (502.3)	715.7 (423.8)	-2.9960	0.0065	-0.6116	0.0258
T2 Acc	Learner s	136.5 (31.8)	153.7 (25.9)	5.9575	0.0000	1.2161	0.0000
T2 RT (msec)	Learner s	477.0 (485.7)	335.6 (205.7)	-1.7880	0.0870	-0.3650	0.1739

Mean and standard deviation (SD in parentheses) represent the values. Differences between conditions were assessed using paired t-Student tests. RT: reaction time, Acc: accuracy.

Discussion paragraph 4:

The inclusion of an active control group allowed us to isolate the specific effects of learning, as we observed no BAGs or cognitive changes associated with active controls. This reflects the genuine

training-related plasticity effects in our pre/post learning design, showing that short, targeted training can improve specific and generalized performance[22].

Discussion paragraph 5:

An additional limitation of our work is the small sample size used for the pre/post-learning design, possibly overestimating the magnitude of the outcomes, and may not fully capture the distinction between long-term expertise and short-term video game training.

Methods: Pre/post-learning design

We used an active control group (N = 12) that was trained in Hearthstone (which is less creative than StarCraft regarding real-time strategic innovation and improvisation), with identical recruitment, training time, and inclusion/exclusion criteria as the StarCraft II group.

Comment 2.5: Given the sample used to train the data probably included more non-experts than experts (experts are rarer in the general population), is it possible that the brain age models were better able to characterize brain age in non-experts? Is there a way you can evaluate this?

Response 2.5: Thank you for raising this point, which has been discussed as follows:

Discussion paragraph 6:

As the sample used to train the data likely includes more non-experts than experts, brain age models may present a bias. However, there are multiple reasons to believe this is not the case. The experimental design controls for typical variables (age, sex, education, and in some domains, additional factors such as general cognition). Differences between BAGs—at least in terms of the variables typically used for group comparisons—are better explained by differences in creativity. Moreover, the consistency of delayed brain age was observed across groups, domains, and comparisons (between-group contrasts and pre/post designs), suggesting a systematic association between creativity and delayed brain age, despite potential random factors. Furthermore, the association and consistent directionality of BAGs with performance measures, along with the same effects in the pre/post design, reinforces this idea. Nonetheless, we cannot fully rule out other contributing factors, and future research should address potential additional confounders.

Comment 2.6: Please clarify where the samples came from: how were participants recruited and based on what criteria. Also, please clarify if the individual datasets are complete (i.e., were the experts and matched non-experts recruited as part of a specific study or was there a larger number of non-experts that you selected from during matching?). In general, the description of the datasets is insufficient: I would like to know, for example, if these datasets have been used individually in past papers or if they were collected specifically with the goal of collating and performing this analysis.

Response 2.6: Thanks for raising this point. We improved the descriptions of all datasets (expertise and learning), including the SVMs training data (**response 1.4**). Further, we clarified that independent studies were used in previous works unrelated to BAGs (**response 1.4**).

Comment 2.7: Please describe the matching process in more detail. I know that matching is challenging across multiple domains, but some differences exist (non-significance is a low bar for matching success): e.g., age in

the dance sample. Please also report normality for age and education to ensure t-tests are appropriate.

Response 2.7: Thanks for your suggestion. We have improved the matching process as detailed in **response 1.4** and addressed the normality for matching as detailed here. After removing the youngest/oldest participants from the non-experts/experts in the dancing group, they were fully age-matched independently of the chosen test. To strictly control the effects of different covariates, we ran an ANCOVA using age, sex, and education. All the effects of expertise on BAGs across all groups remained consistent (**Table S8** in **Supplementary Materials**)

Results: Sensitivity analyses

ANCOVAs controlling for age, sex, and education confirmed that expertise significantly predicted BAGs across all creative domains (tango dancers, musicians, visual artists, and gaming). These effects remained significant after FDR correction, supporting the robustness of our main findings (**Table S8** in **Supplementary Materials**).

Table S7. Matching and normality testing for the expertise design groups.

Domain	T-test (age)	MWU (age)	T-test (edu)	MWU (edu)	Normality
Tango dancers	t=1.32, p=0.1942	U=338.50, p=0.1051	t=0.48, p=0.6348	U=295.00, p=0.5008	No normality (p < 0.05)
Musicians	t=0.67, p=0.5077	U=439.50, p=0.7731	t=1.71, p=0.0931	U=525.00, p=0.0919	No normality (p < 0.05)
Visual artists	t=0.07, p=0.9418	U=109.50, p=0.9167	NA	NA	No normality (p < 0.05)
Gaming	t=0.34, p=0.7319	U=494.50, p=0.8486	t=-0.76, p=0.4531	U=415.00, p=0.3559	No normality (p < 0.05)

Normality was assessed using the Shapiro-Wilk test, where a *p*-value < 0.05 indicates the lack of normality in data. All groups are matched by education and age, both using t-Student and Mann-Whitney (MWU) tests. All groups are fully matched independently of the chosen test.

Table S8. Sensitivity analysis using ANCOVA.

Groups	Model components		coef	std err	t values	p values	p (FDR-corrected)
Tango	Baseline	Intercept	20.8885	10.392	2.01	0.051	-
	Confounders	Sex	2.0209	2.797	0.723	0.474	-
		Age	-0.8504	0.231	-3.677	0.001	-
		Education	0.549	0.458	1.198	0.238	-
	Predictor	Expertise	-5.8611	2.563	-2.287	0.027	0.036
Music	Baseline	Intercept	18.2994	8.789	2.082	0.042	-
	Confounders	Sex	1.0512	2.516	0.418	0.678	-
		Age	-0.506	0.177	-2.862	0.006	-
		Education	0.0161	0.358	0.045	0.964	-
	Predictor	Expertise	-4.7632	2.38	-2.002	0.049	0.049

Visual arts	Baseline	Intercept	22.5092	6.101	3.689	0.001	-
	Confounders	Sex	1.3454	2.417	0.557	0.583	-
		Age	-0.5983	0.213	-2.811	0.009	-
	Predictor	Expertise	-6.0263	2.041	-2.952	0.007	0.018
Gaming	Baseline	Intercept	29.7519	4.984	5.97	0	-
	Confounders	Age	-0.9793	0.242	-4.049	0	-
		Education	-0.1125	0.312	-0.361	0.72	-
	Predictor	Expertise	-3.8048	1.409	-2.701	0.009	0.018

The model included the brain age gaps (BAGs) as the dependent variable, and the group (expertise), age, education, and sex as predictors. The effect of expertise is significant across all the groups after FDR correction.

Method: Sensitivity analyses

Sensitivity analysis 1: To control for potential confounding variables, we conducted an ANCOVA with BAGs as the dependent variable and the expertise (group), age, sex, and education as covariables. This analysis was performed separately for each creative domain (tango dancers, musicians, Visual artists, and gaming). Normality tests and matching using parametric and non-parametric tests are reported in **Table S7** in **Supplementary Material**.

Comment 2.8: Having worked with brain age data before, I know that sometimes brain age gaps can be correlated with age, making it somewhat complicated to disentangle this variance. Was this true in your data, and did you think about how to control for this?

Response 2.8: Thank you for raising this issue. In **response 2.7,** we controlled for covariables. Our BAGs accounted for regression to the age mean bias[23-25]. This has been clarified in the manuscript (see the section **Brain clock models and brain age gaps** in **Methods**). We also confirm that performing the analysis with no age bias correction does not alter the main effects. We added this as an extra sensitivity analysis as follows:

Results: Sensitivity analyses

We compared BAG estimates with and without age bias correction. The main group effects remained consistent across approaches (**Figure S10** in **Supplementary Materials**), confirming that our findings are not driven by regression-to-the-mean artifacts.

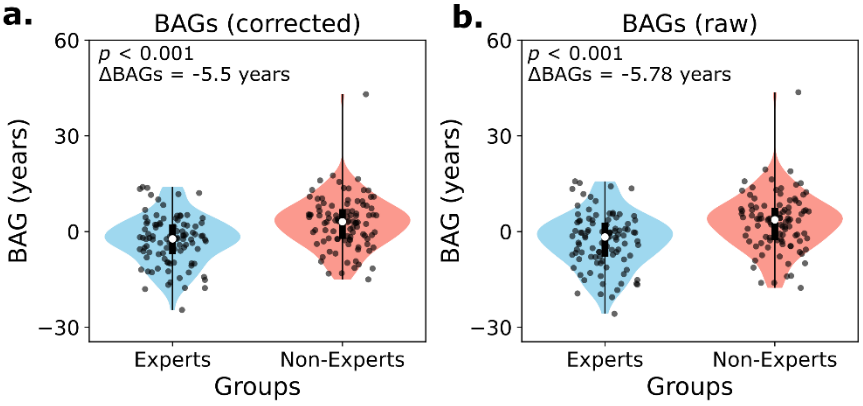

Figure S10. BAGs with and without age bias correction. We compared all experts and non-experts combined. Results are similar regardless of the approach employed. **a.** BAGs with age bias correction: ΔBAGs = -5.50, t = -4.823, $p = 2.86 \times 10^{-6}$, D = -0.69, FDR-corrected. **b.** BAGs without age bias correction: ΔBAGs = -5.78, t = -4.625, $p = 6.82 \times 10^{-6}$, D = -0.66, FDR-corrected. Box plots were built using the median, 1st and 3rd quartiles, and the

maximum and minimum values of the distributions. Results remained unchanged after removing outliers, i.e., values 2 standard deviations from the mean, for **a.** with correction (t = -5.496, p = 1.25×10^{-7}), and **b.** without correction (t = -5.621, p = 6.90×10^{-8}).

Method: Sensitivity analyses

> Sensitivity analysis 2: Further, we analyzed the effects of age bias correction comparing the experts and non-experts with and without the correction.

Comment 2.9: Your brain age model only explains about half of the variance associated with age (r=.742, R2=55%). You cite several papers suggesting that this is in line with other work but the original Cole et al., "brain age predicts mortality" paper (using structural MRI) had an R2 of 88%. These methods clearly work best if there is a strong correlation between predicted and actual age and while I think yours may be adequate it will produce a lot of noise in brain age gap estimates. I think you can make this clearer as a limitation.

Response 2.9: Thank you for this comment. We now cite works using EEG for brain age prediction, and we discussed the expected effects of interventions and lifestyle on brain aging.

Results: Second paragraph

> The training brain clock model yielded robust results. Accurate age estimation performance was consistent with previous studies using the mean absolute error (MAE) and Pearson correlation[23,26].

Discussion paragraph 6:

> Finally, our results are consistent with previous findings on the effects of lifestyle[23] and interventions[27] on brain aging, including the scalability of these effects[27]. Moreover, they align with the notion that lifestyle and intervention-related changes tend to produce smaller effects than those observed in patient-control comparisons[23,24].

Minor points:

Comment 2.10: I do not think the term "brain health clocks" is appropriate. We do not really know much about brain age as a measure and the extent to which it reflects brain health. I would prefer a more descriptive term (e.g., brain age or brain age gap) that has been more extensively used in the literature to refer to this specific type of method. "Brain clocks" is better but still seems more confusing than just using one term (brain age) throughout.

Response 2.10: Thank you for raising this point. After a discussion with the co-authors, we have followed your suggestion and now use "brain clocks" throughout the titles and manuscript.

Comment 2.11: Please be clear when you are talking about brain age gaps: sometimes you use words like "larger" which could mean more positive or more positive/negative. Using directional terms avoids confusion (e.g., accelerated vs. decelerated brain aging). Another example: "Conversely, people with healthier habits such as systematic creative experiences could be hypothesized to have shorter BAGs". Would you not expect these gaps to be larger but in the negative direction (brain age lower than chronological age)?

Response 2.11: Thanks for these suggestions. We reviewed the entire manuscript using terms that indicate the direction of the differences and associations.

Comment 2.12: I do not typically think of occipital regions as particularly age-vulnerable. Can you provide some references to justify this hypothesis? Reading the references you provide they do not seem to suggest this: the main mention of occipital cortex in reference 32 reads as follows: "Regional heterogeneity of synaptic vulnerability in ageing has also been suggested. Similarities in protein expression between young and old occipital cortex synaptic populations in nonhuman primates and humans indicated resistance to age-related damage in this region, whereas the hippocampal synaptic population displayed proteomic heterogeneity on ageing, possibly indicating selective vulnerability of aged hippocampal synapses (Graham et al., 2019). Indeed, resistant (occipital cortex) and vulnerable (hippocampal) brain regions seemed to age differently in nonhuman primates and humans." This paragraph suggests the occipital cortex is resistant to aging. Reference 34 similarly states: "Subsequently, we modeled cortical thickness changes over lifespan, revealing that fronto-temporo-parietal hubs were among the brain regions that changed the most, whereas occipital hubs showed a quite spared cortical thickness across ages." Although reference 33 does show differences in occipital regions my general understanding of the literature is that these regions are less vulnerable to aging. It is also surprising that you do not mention the hippocampus or DMN: regions known to be vulnerable to aging.

Response 2.12: Thank you for raising this observation. We have better clarified that (a) the main connections affected by aging involved mainly frontal regions (**Figure 1**, **Figure 4**, and Supplementary **Figure S2**) and (b) toned down the statements related to parieto-occipital hubs.

Introduction paragraph 4:

> The lower BAGs in people with more creative experiences would be observed in frontoparietal hubs, as these are regions vulnerable to aging[28-31].

Results paragraph 2:

> BAGs were associated with reduced connectivity in age-related networks, specifically frontoparietal and frontal-to-occipital connections[28-31] (**Figures 2B-C** and **Figure S2** in **Supplementary Materials**).

Results: The BAG networks comprise regions with age-related vulnerability and expertise

> In the expertise study, a correlation between age-related vulnerability regions and changes in nodal strength (r = 0.345, p = 0.0012, Cohen's f^2 = 0.135, spin test, FDR-corrected, **Figure 4A**) involved frontoparietal hubs.

Discussion paragraph 3:

> Creativity experiences have been linked to structural and functional changes in brain regions vulnerable to aging and involved in creativity-related cognitive processes[28-31]. These consisted mainly of frontoparietal hubs, critical for attention, motor control, coordination, and rhythm[31-35].

Comment 2.13: Given brain age can be calculated using different modalities and is most commonly done using structural brain data, I think it would help the reader if you clarified that it was "EEG brain age" or at least "functional brain age" throughout the paper to differentiate from structural approaches.

Response 2.13: We now use the term "functional BAGs" in the manuscript and clarified the computation of BAGs.

Comment 2.14: You only talk about age-associations in terms of vulnerability. It might be worth including in the discussion that some age-related regions could be involved in processes that improve with age (e.g., semantic knowledge, emotional well-being) or are compensatory for aging.

Response 2.14: We appreciate your comment and have added the following text:

Discussion paragraph 5:

> Accelerated aging, as measured with increased positive BAGs in literature, is typically associated with adverse outcomes, including disease, unhealthy lifestyles, or increased disparities[23,24]. However, future studies should develop methodologies that disentangle the potential positive healthy aging outcomes.

Reviewer #3:

Remarks to the Author:

Comment 3.1: The manuscript titled "Creative Experiences and Brain Health Clocks" aims to enhance our understanding of how various creative experiences influence the functional connectivity characteristics of brain networks and assesses the implications of these changes on brain aging. The findings suggest that creative expertise may slow down brain aging, as reflected by functional connectivity alterations. Furthermore, by employing a longitudinal design, the study demonstrates that changes associated with slowed aging are also evident when individuals engage in creative experiences, specifically through playing video games. While I find the results intriguing and relevant to the broad readership of Nature Communications, with potential transformative implications for promoting brain health through everyday activities, I do not believe that the manuscript, in its current form, provides sufficiently robust evidence to support its claims. However, I believe that most of my concerns can be resolved with substantial but feasible revisions. Below, I outline the major limitations that form the basis of my decision.

Response 3.1: Thank you for the feedback regarding our manuscript. Below, we have addressed each concern individually.

Comment 3.2: Lack of adequate information in the introduction. The final paragraph presents multiple hypotheses that are not well justified. These read more like a foreshadowing of the results rather than a rational anticipation of testable hypotheses. Improved integration with prior literature could strengthen this section. For example, it is unclear why the study hypothesizes that creative experiences would be associated with delayed brain aging "especially in expertise study" but "to a lesser extent in the pre/post-learning study." Such hypotheses require clearer justification or should be revised.

Response 3.2: Following your observation, we have improved the hypothesis while keeping the original predictions intact, as we do not want to create *ad hoc* specific hypotheses based on the observed results. We have also provided better justification for related antecedents in the introduction (**responses 1.2, 3.3, 3.4**, and **3.5**). The new paragraph on the hypothesis now reads as follows:

Introduction paragraph 4:

> We hypothesize that cross-domain creative experiences are associated with delayed brain aging as measured by BAGs[8,9,23,24]. The lower BAGs in people with more creative experiences would be mainly observed in frontoparietal hubs, as these are regions vulnerable to aging[28-31]. An association between

delayed brain aging and the degree of creative expertise should be observed in experts (e.g., years of practice) and learners (pre/post-learning outcomes)[14,15]. These effects may be larger in the expertise study that involves long-term training than pre/post effects in non-expert participants involving short-term learning[36]. Using biophysical modeling and graph theory, we hypothesize that creative experiences are related to neural plasticity-driven functional alterations[14,37-41], such as increased local and global efficiency[14,15,42,43] and modulations in biophysical coupling[44-47], as previous reports show impaired segregation and reduced connectivity strength in aging[48-50]. The design allows us to investigate cross-domain convergent impacts of creativity on BAGs, with specific mechanisms related to these potential protective effects, providing new insights about the impact of cumulative creative experiences on brain health. These results may inform future public policies to improve health and well-being through creativity and the arts[51].

Comment 3.3: The manuscript posits that since "frontoparietal and occipital hubs" are most vulnerable to aging, creative experiences would primarily impact these regions. While frontoparietal regions are consistently reported as aging-sensitive, the involvement of occipital regions is less well-supported. Even within the cited literature, other vulnerable regions—such as centrotemporal areas—are mentioned, which are left out by the current article. Additionally, multiple studies indicate that aging affects regions within the default mode network (DMN) and subcortical structures, yet these are overlooked in both the introduction and discussion.

Response 3.3: Thanks for your suggestion. We have addressed it in response **2.12**.

Comment 3.4: The introduction claims that the "combination of graph theory and biophysical modeling" with brain age gap (BAG) measures can provide insight into brain aging mechanisms. However, this claim appears without context. The manuscript should provide appropriate references explaining why these specific methods are particularly informative.

Response 3.4: We appreciate your observation and have made the following changes in the manuscript:

Introduction paragraph 2:

A critical concern in the literature on the health effects of creative experiences is the lack of mechanistic insight[11]. Biophysical models and graph theory can help elucidate general mechanisms of aging, such as the inter-area coupling modulated by plasticity and the underlying organization of brain networks[52-61]. These mechanisms and organizational features, as revealed through generative models and graph-theoretical approaches, could inform the properties of brain clocks and their potential modulation by creative experiences.

We also improved our hypothesis while keeping the original predictions unaltered (see **response 3.2)**.

Comment 3.5: Brain age gap estimates can be derived from different imaging modalities (e.g., structural, volumetric, connectivity, or functional measures). The introduction appears to conflate these measures as if they are directly comparable, but this assumption is not well-established. I recommend avoiding interpretations of structural brain age findings to justify functional brain aging analyses unless explicitly supported by prior work.

Response 3.5: Thanks for this observation. We have clarified in the revised manuscript that our study exclusively focuses on functional BAGs, and that we do not intend to conflate or directly compare them with structural BAGs. We also clarified in the Introduction that functional BAGs are more suitable for capturing lifestyle changes and short-term intervention effects, such as those examined in the design.

Introduction paragraph 2:

> Only a few works addressed the impact of creativity, specifically music expertise, on brain structural correlates and reported contradictory results[8,9]. While structural and functional BAGs can be estimated, the latter may be more sensitive to plasticity-driven effects of interventions and lifestyle on brain aging[24,27].

Comment 3.6: 2. Methodological Concerns: Overinterpretation of Multi-Modal Data Integration (This is my most significant methodological concern). The study combines three different measures of functional connectivity across datasets: [1] EEG-derived functional connectivity (FC) [2] MEG-derived FC [3] Simulated EEG from diffusion tensor imaging (DTI). The manuscript argues that "EEG and MEG capture similar neural and can be integrated into a single pipeline." However, this is a problematic assumption. EEG and MEG have fundamental differences (see Coquelet et al., 2020, NeuroImage), and even within similar areas, correlations between EEG- and MEG-derived connectivity measures are modest (below 0.5). The cited justification for integration (Muthuraman et al., 2015) supports using concurrent EEG and MEG recordings for robust signal capture but does not endorse combining EEG and MEG connectivity data from different subjects in a unified comparison. The inclusion of simulated EEG from DTI is even more concerning. While EEG simulations from structural data provide insights into large-scale dynamics, they remain an approximation of real neural activity and cannot be assumed to be equivalent to either EEG or MEG data. Suggested solution to resolve this critical issue: Instead of pooling datasets to increase sample size, I strongly recommend performing separate analyses for each dataset (dancers, musicians, visual artists, and gaming). If similar spatial patterns and age-related effects emerge across independent tests, this would provide strong cross-modality replication without introducing confounds related to data integration across modalities.

Response 3.6: Thank you for pointing out this critical issue, which we have addressed through several approaches, as detailed below.

Regarding M/EEG: We toned down the assumption that these metrics can capture similar neural activity and clarified that the BAGs estimation allows to compare different functional technique[62]".

Regarding domains: We performed individual domain analyses for (a) age vulnerability and connectivity effect sizes and (b) associations between expertise and BAGs. The results remained consistent at the individual domain level. In addition, we (c) compared the expertise between participants with lower BAGs (BAG-younger) and higher BAGs (BAG-older). As shown in the results provided in Figure 3A, participants with delayed brain aging possess higher expertise. Finally, we (d) acknowledge the inclusion of simulated EEG from DTI as a limitation of our work.

We made the following changes in the manuscript:

Results: Sensitivity analyses

> We found that the spatial correlations between age vulnerability and connectivity effect sizes remained significant when analyzed separately for each domain. Specifically, robust positive correlations were observed for tango dancers (r = 0.333, p = 0.0016, f^2 = 0.125, spin test, FDR-corrected), musicians (r = 0.350, p = 0.0012, f^2 = 0.140, spin test, FDR-corrected), visual artists (r = 0.246, p = 0.013, f^2 = 0.064, spin test, FDR-corrected), and gaming (r = 0.282, p = 0.01, f^2 = 0.086, spin test, FDR-corrected) (**Figure S8** in **Supplementary Materials**).

Participants classified as BAG-younger (i.e., with brain age gaps below the 35th percentile) showed significantly higher domain-specific expertise than those classified as BAG-older (i.e., above the 65th percentile). This pattern was consistent across domains: tango dancers (U = 118.5, p = 0.0160, FDR-corrected), musicians (U = 26.5, p = 0.0300, FDR-corrected), visual artists (U = 138, p = 0.0080, FDR-corrected p = 0.0160), and gaming (U = 69.5, p = 0.005, FDR-corrected). Results are summarized in **Table S9** in **Supplementary Materials.**

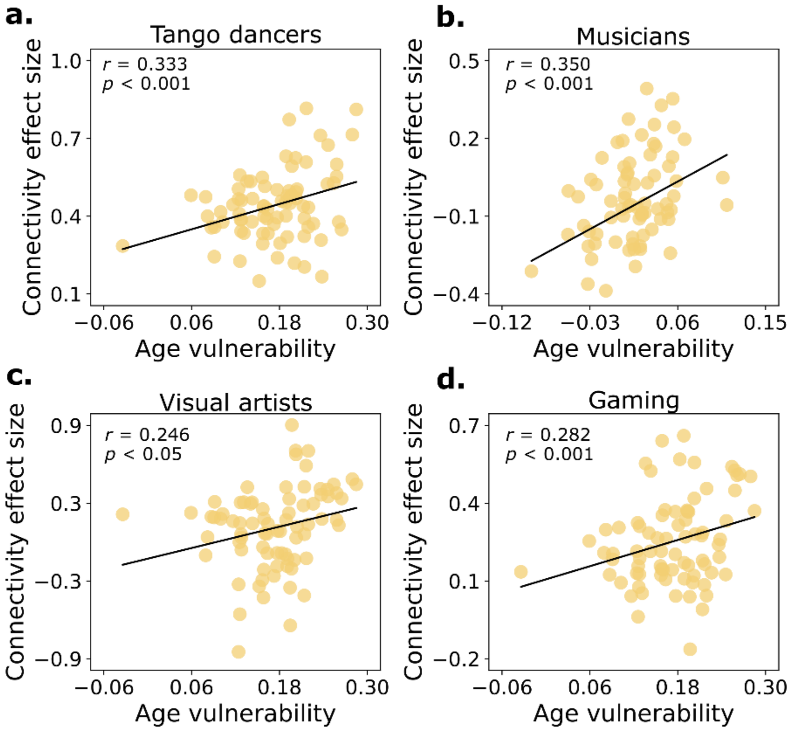

Figure S8. Spatial correlations for each domain in the expertise design. Age vulnerability versus connectivity effect size in **a.** tango dancers (r = 0.333, p = 0.0016, Cohen's f^2 = 0.125); **b.** musicians (r = 0.350, p = 0.0012, Cohen's f^2 = 0.140); **c.** visual artists (r = 0.246, p = 0.013, Cohen's f^2 = 0.064); **d.** gaming (r = 0.282, p = 0.01, Cohen's f^2 = 0.086). We used the EEG-derived age vulnerability map for groups related to tango dancers, musicians, and visual artists. For music expertise, we built the age vulnerability map from musicians' and non-musicians' MEG data. Points represent participants. Before FDR correction, the p-values were computed using the Spin test up to 10000 permutations.

Table S9. Domain-specific differences in expertise between BAG-younger and BAG-older groups.

Groups	BAG-younger expertise Mean (SD)	BAG-older expertise Mean (SD)	MWU	p-value	p (FDR-corrected)
Tango dancers	16.32 (13.63)	5.10 (6.58)	118.5	0.0118	0.0160
Musicians	3.64 (2.50)	0.86 (1.91)	26.5	0.0300	0.0300
Visual artists	8.20 (8.48)	3.45 (6.49)	138	0.0080	0.0160
Gaming	40.06 (46.18)	18.81 (46.37)	69.5	0.0013	0.0050

We classified participants as BAG-younger or BAG-older based on the overall BAG distribution (combining experts and non-experts). Specifically, individuals with BAG values below the 35th percentile were labeled as

BAG-younger, while those above the 65th percentile were labeled as BAG-older. Mean and standard deviation (SD in parentheses) represent the values. Differences between groups were assessed using one-size Mann-Whitney (MWU) tests. *P*-values were FDR-corrected.

Methods: M/EEG acquisition, preprocessing, and connectivity

We changed "Despite their differences, MEG and EEG capture similar neural activity and can be integrated into a single pipeline" for "BAGs derived from different functional sources can be compared using harmonization steps[62]".

Method: Sensitivity analyses

Sensitivity analysis 3: We repeated the spatial correlation analyses between age vulnerability maps and connectivity-based effect sizes separately for each domain of expertise. For the tango dancers, musicians, and visual artists groups, we used the EEG-derived age vulnerability maps; for the gaming group, the age vulnerability map was constructed using MEG data from musicians and non-musicians.

Sensitivity analysis 4: We investigated whether the BAGs are associated with domain-specific expertise levels. Participants were classified as BAG-younger or BAG-older based on the overall BAG distribution, merging experts and non-experts. Specifically, individuals with BAG values below the 35th percentile were labeled as BAG-younger, while those above the 65th percentile were labeled as BAG-older. We then compared expertise scores between these two groups using one-size Mann-Whitney U tests and FDR correction.

Discussion paragraph 4:

Our approach applies the same BAG estimation method across different functional techniques by standardizing the signals and integrating them into a shared space, as done in other functional studies[23,24] Moreover, our results were confirmed at the individual dataset level and showed a consistent pattern across studies, confirming the expected BAG modulation within each domain. However, future research should explore in greater detail how different functional techniques impact BAG estimation.

Sensitivity analyses 1 and 2 can be found in **responses 2.7** and **2.8**.

Discussion paragraph 5:

While EEG simulations derived from structural data (DTI) offer valuable insights into large-scale brain dynamics[14,63]. EEG simulations and other datasets showed similar BAG effects. However, the former remain approximations of actual neural activity and should, therefore, be interpreted with caution.

Comment 3.7: Confounding Variables: Several confounds could influence the reported effects: Individuals engaged in creative activities may differ in socioeconomic status or exposure to adversity, both of which are known to influence BAG (see Cohen et al., 2023, Dev Psychol; Rakesh et al., 2023, Trends Cogn Sci). If such variables are available, I recommend including them in sensitivity analyses or otherwise acknowledging them as limitations.

Response 3.7: Thank you for your observation. In the **responses 2.3** and **2.7**, we have added additional information on the matching procedures based on age, sex, education, and geography. In addition, in two data sets (gaming and tango dancers), the groups were also matched by cognitive abilities. Furthermore, as clarified in responses **2.4** and **3.8**, the pre/post-learning design with the additional active control group helps to control

these confounding factors. We have also additionally controlled the effects by demographics variables with an ANCOVA (**response 2.7**).

Finally, we have added this text to the manuscript (Discussion paragraph 6):

> Demographic and individual factors are known to influence BAG[23,64,65]. Our comparisons controlled for standard variables, including age, sex, education, and geography (and cognition in the gaming and tango dancers). Likewise, the pre/post-learning intrasubject design controls all confounding and familial effects with an active control condition. Nonetheless, future studies should examine how the protective effects of creative experiences are potentially influenced by socioeconomic status and other individual-level variables.

Comment 3.8: In the longitudinal study, post-learning scans show lower BAGs than pre-learning scans which is interpreted as the effect of the creative experience. However, this could be partially driven by increased familiarity with data collection procedures rather than true neurobiological change. If longitudinal data are available from individuals who did not undergo learning interventions, their trajectories should be examined to control for this potential confound. Otherwise, this should at the very least be indicated as a limitation due to the inavailability of such data.

Response 3.8: Thanks for raising this issue. We have addressed this concern in response **2.4** by incorporating an active control group.

Comment 3.9: Statistical Issues in Spatial Comparisons: The manuscript correlates different spatial maps (e.g., Fig. 4A, comparing age vulnerability maps with nodal strength differences) using Pearson's correlation p-values. This method is known to inflate false-positive rates due to spatial autocorrelation (see Markello et al., 2021, NeuroImage). More robust statistical approaches have previously been proposed to correct this issue, such as: [1] Spin tests (Alexander-Bloch et al., 2018, NeuroImage), [2] Moran spectral randomization (Wagner et al., 2015), and [3] Generative models accounting for spatial autocorrelation (Burt et al., 2020, NeuroImage). These methods are implemented in software such as NeuroMaps (Markello et al., 2022, Nat Methods) and can easily be applied instead of a simple Pearson's correlation. I strongly recommend re-evaluating spatial correlations using these techniques. Please note that this comment concerns all spatial comparisons (e.g., including the comparisons with Neurosynth maps).

Response 3.9: Thanks for sharing your concern. We have repeated all the correlation analyses using the Spin Test implementation in Python (BrainSMASH). When correcting p-values with the Spin Test and FDR correction, we replicated the same effects. The following text has been added to the manuscript:

Figures 4, S7, and S8: We added the assessment of p-values using the spin test: Before FDR correction, the p-values were computed using the Spin test up to 10000 permutations.

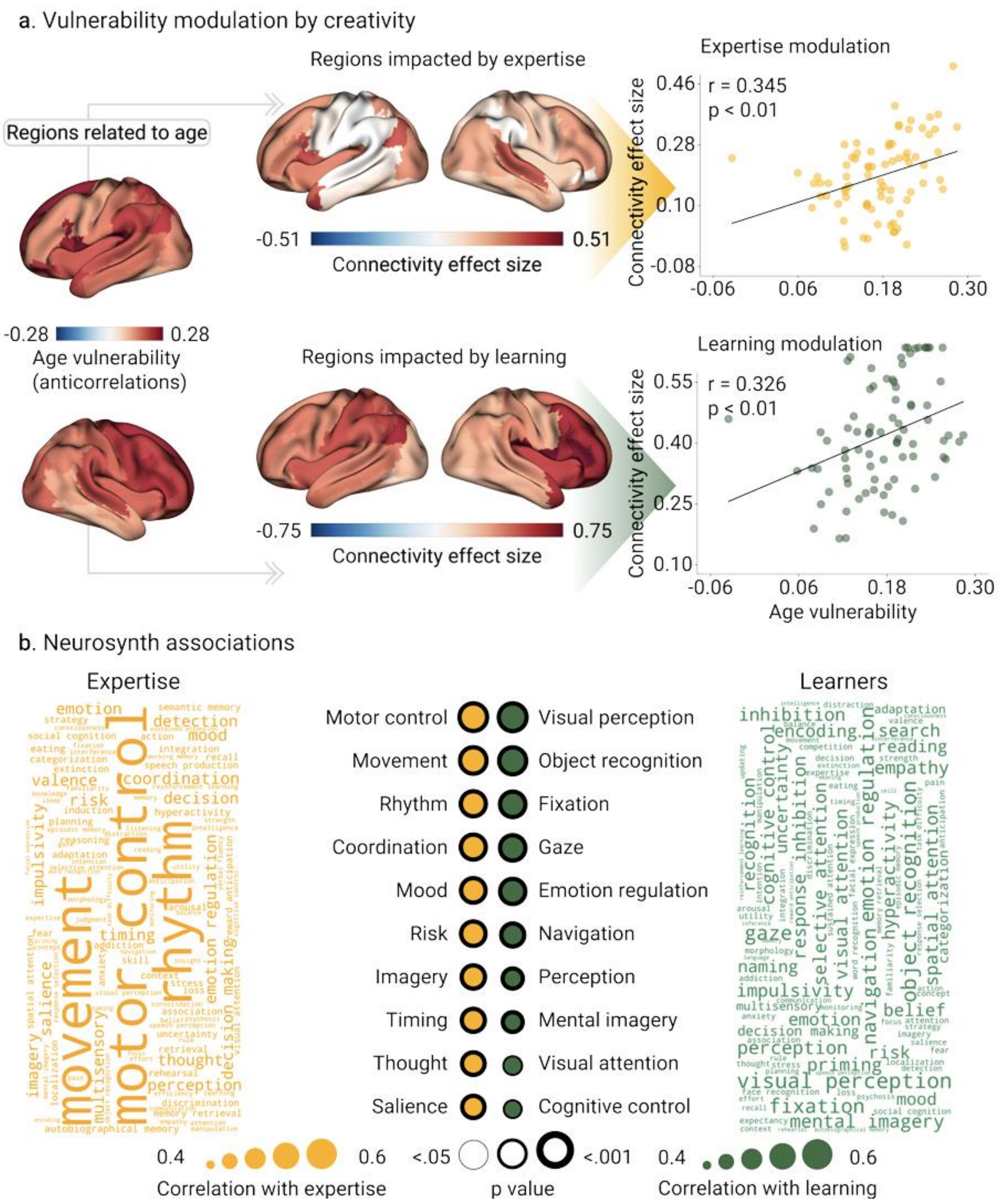

Figure 4. Topographic patterns of connectivity associated with creative experiences. A) The anticorrelation between nodal functional connectivity and age represents age vulnerability. Associations between age vulnerability and brain connectivity increase are driven by creative experiences. The areas that have the greatest increase in connectivity are the ones with higher Cohen's D effect sizes. The brain of the experts' group is the average brain of tango dancers, musicians, visual artists, and gaming experts. Points represent brain areas. B) *Neurosynth* associations with brain connectivity increase by creative experiences. We reported the absolute Pearson correlation between brain connectivity and association maps of different cognitive processes. FDR-corrected *p*-values are shown, and the thickness of the circles represents statistical significance. The p-values were computed using the Spin test up to 10000 permutations before performing the FDR correction.

Methods: Brain maps and Neurosynth associations:

To account for potential spatial autocorrelation in brain maps, we conducted Spin Test analyses[20] using the BrainSMASH Python library (https://brainsmash.readthedocs.io/)[21]. We applied this method to all correlation analyses involving cortical surface data. The Spin Test generates spatially constrained null models by randomly rotating the spherical projection of cortical surface maps while preserving their spatial structure. We used 10000 permutations to generate null distributions of correlation values. Empirical correlations were then compared to these null distributions to obtain p-values corrected for spatial autocorrelation. We additionally applied false discovery rate (FDR) correction across all comparisons.

Comment 3.10: Justification for Biophysical Modeling: The role of biophysical modeling and global coupling in this study remains unclear. Since these results are not reflected in the longitudinal analyses, their added value is questionable. Many connectivity metrics are interrelated, and it is unclear whether global coupling provides meaningful additional insights beyond traditional graph-theoretical measures such as global efficiency. I think it might help the overall readability of the manuscript if this information is left out. Otherwise, the article should provide additional discussion on the specific knowledge that was revealed by the additional step of biophysical modeling.

Response 3.10: We appreciate your observation. We have provided a clear explanation in **response 3.4** regarding the relevance of assessing brain mechanisms of BAG using biophysical models and graph theory.

Comment 3.11: Issues with Data Visualization: The cortical projections of spatial brain maps lack clear delineations of the AAL atlas regions and appear to imply a high-resolution spatial map, which is not what is being analyzed. I recommend revising the visualizations to more accurately reflect the resolution of the data.

Response 3.11: Thanks for this suggestion. We re-designed the brain plots, avoiding surface smoothing and plotting the original AAL delineations (see the changes in **responses 3.9** and **3.25**).

Comment 3.12: Important connections predicting age: Figure 2.b presents a comparison of connections that were "most important" for predicting age. I could not find any explanation of how this was measured in the manuscript. Could the authors clarify?

Response 3.12: Thanks for raising this issue. The most important connections are the weights of the trained SVM. We clarified this in the new MS:

Figure 2 Caption:

Top connections reflect the highest absolute SVR weights, indicating their importance in age prediction.

Methods: Brain clock models and brain age gaps

Feature importance was defined as the absolute value of SVR weight coefficients, averaged across cross-validation folds and repetitions.

Supplementary Methods (Section 1.7):

We extracted feature importance by averaging the absolute SVR weights across folds and repetitions, identifying the most predictive connections for brain age.

Comment 3.13: Choice of colormaps: Figure 4.a uses an unconventional colormap with a sharp transition from cyan to pink somewhere arbitrary in the value range, which is not good practice. I would suggest changing the colormaps to perceptually uniform alternatives (e.g., viridis/plasma) to avoid producing inaccurate perceptions. Moreover, for ranges that include both positive and negative ranges, I would suggest using a symmetric diverging colormap centered at zero to distinguish positive vs. negative effects.

Response 3.13: Thank you for the suggestion. We have changed the colormap to a diverging colormap (inverted Red and Blues, see **response 3.25**).

Minor points:

Comment 3.14: The manuscript had several typos/errors. Here are a few that I spotted (but this is not a complete list; I would recommend checking the whole document to ensure such errors are all fixed):

Response 3.14: We have corrected all typos and grammar errors.

Comment 3.15: line 141: shorter BAGs -> lower BAGs.

Response 3.15: Corrected.

Comment 3.16: line 155: groups of experts and non-experts tango dancers -> groups of expert and non-expert tango dancers.

Response 3.16: Corrected.

Comment 3.17: line 193: withing -> within.

Response 3.17: Corrected.

Comment 3.18: > line 228: The -> Then.

Response 3.18: Corrected.

Comment 3.19: > Line 273: undelaying -> underlying?

Response 3.19: Corrected to “underlying”.

Comment 3.20: In the first paragraph of the results section, I recommend including the age range of the participants in the dataset. I know this is later mentioned in the methods and supplementary material, but adding that information to the results could improve the overall readability.

Response 3.20: We now include the age range of the participants across all datasets at the beginning of the Results section and summarize them here: a) tango dancers (18-50 years), b) musicians (22-41 years), c) Visual artists (20-37 years), d) gaming (18-31 years), and e) pre/post-learning (20-30 years).

Comment 3.21: In Figure 2.a the BAG predictions are shown in a scatter plot against chronological age. I recommend including a supplementary figure that shows the predicted age (before accounting for the regression to the mean effect) to better reflect the actual model accuracy. It should be noted that even a random prediction with small variance may lead to low MAE and high correlations after accounting for RTM, and it is hence highly

recommended to also report and visualize the accuracy before accounting for RTM to better portray model performance.

Response 3.21: Thanks for raising this issue. Figure 2 scatter plot shows chronological age versus predicted age, and these values were not corrected by the age bias. We only applied age bias correction in the out-of-sample validation (the expertise and pre/post-learning designs). We compared the BAGs with and without the correction, and the results remained unchanged (**response 2.8**). Further, our results were replicated when fully controlled by age and other covariables (**response 2.7**)

Comment 3.22: Several previous works in the intersection of graph theory metrics and brain aging have reported changes in local efficiency, segregation, integration, and global efficiency to be linked to aging. I'd recommend citing a few of such previous works to further link with the existing literature.

Response 3.22: Thanks for this suggestion, and the following clarification has been provided

Discussion paragraph 3:

> Finally, many studies have assessed graph properties associated with aging[48-50], but no previous study has used them to describe the organizational properties of BAGs. Our results suggest that increased local efficiency with lower BAGs is similar to other brain organizational changes observed with aging, i.e., the reduction of brain segregation[48-50].

Comment 3.23: The sentence in lines 257 to 259 was unclear to me. Did the authors mean to say global coupling instead of local efficiency? I don't see how the two are linked.

Response 3.23: Yes, we have corrected this to “global coupling”.

Comment 3.24: Figure 1.D seems to suggest that both EEG and MEG data were available for all domains, which is not the case. (it could maybe say EEG "or" MEG instead of EEG "&" MEG).

Response 3.24: Thanks. We now specify in Figure 1 in which dataset EEG or MEG are available.

Comment 3.25: In Figure 4.b, for the significant cognitive processes (reported in the middle), it would also be good to include the cortical map of each cognitive process to help interpretability of the figure for the reader.

Response 3.25: Thanks for your suggestion. We have reported the cortical maps related to each cognitive process in the new manuscript. To avoid overloading **Figure 4**, we have provided maps in a supplementary figure (**Figure S9** in **Supplementary Material**).

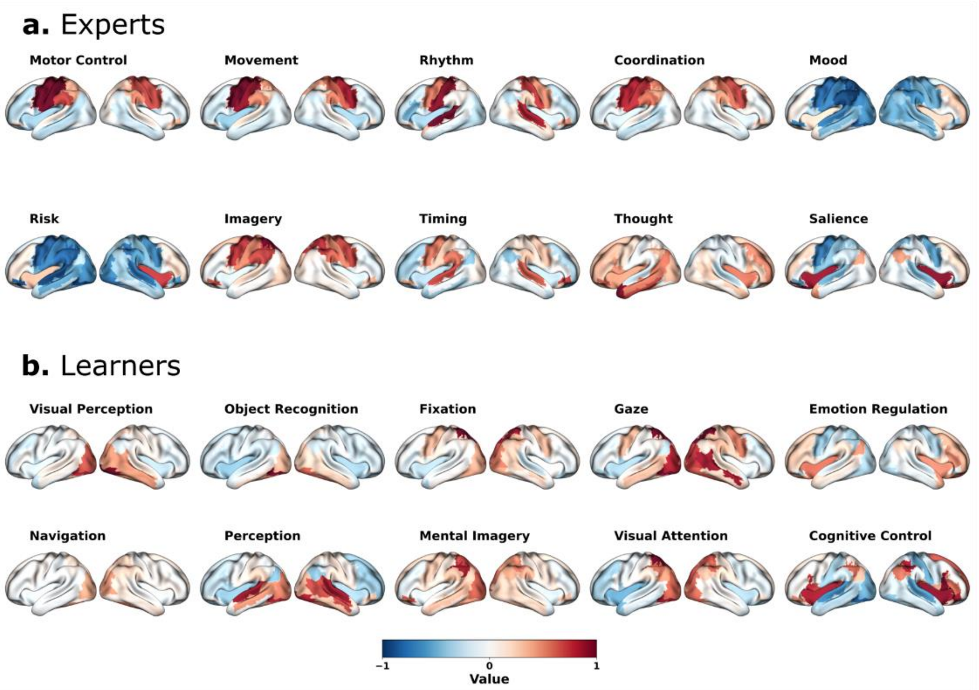

Figure S9. Brain association maps associated with the top 10 correlations with cognitive terms and connectivity effect size in **a.** experts and **b.** learners. For visualization purposes, all maps were normalized to be in the range [-1,1].

References

1 Runco, M. A. & Jaeger, G. J. The standard definition of creativity. *Creativity research journal* **24**, 92-96 (2012).

2 Mercier, M. & Lubart, T. Video games and creativity: The mediating role of psychological capital. *Journal of Creativity* **33**, 100050 (2023).

3 Ganter-Argast, C., Schipper, M., Shamsrizi, M., Stein, C. & Khalil, R. The light side of gaming: creativity and brain plasticity. *Frontiers in Human Neuroscience* **17**, 1280989 (2024).

4 Jackson, L. A. *et al.* Information technology use and creativity: Findings from the Children and Technology Project. *Computers in human behavior* **28**, 370-376 (2012).

5 Blanco-Herrera, J. A., Gentile, D. A. & Rokkum, J. N. Video games can increase creativity, but with caveats. *Creativity Research Journal* **31**, 119-131 (2019).

6 Shi, B., Cao, X., Chen, Q., Zhuang, K. & Qiu, J. Different brain structures associated with artistic and scientific creativity: a voxel-based morphometry study. *Scientific Reports* **7**, 42911 (2017). https://doi.org/10.1038/srep42911

7 Zhu, W. *et al.* Brain structure links everyday creativity to creative achievement. *Brain and Cognition* **103**, 70-76 (2016).

8 Matziorinis, A. M., Gaser, C. & Koelsch, S. Is musical engagement enough to keep the brain young? *Brain Structure and Function* **228**, 577-588 (2023).

9 Rogenmoser, L., Kernbach, J., Schlaug, G. & Gaser, C. Keeping brains young with making music. *Brain Structure and Function* **223**, 297-305 (2018).

10 Klug, S. *et al.* Learning induces coordinated neuronal plasticity of metabolic demands and functional brain networks. *Communications biology* **5**, 428 (2022).

11 Skov, M. & Nadal, M. Can arts-based interventions improve health? A conceptual and methodological critique. *Physics of Life Reviews* **53**, 239-259 (2025). https://doi.org/https://doi.org/10.1016/j.plrev.2025.03.003

12 Hernandez, H. *et al.* Brain health in diverse settings: How age, demographics and cognition shape brain function. *NeuroImage* **295**, 120636 (2024). https://doi.org/10.1016/j.neuroimage.2024.120636

13 Ponce-Alvarez, A. & Deco, G. The Hopf whole-brain model and its linear approximation. *Scientific reports* **14**, 2615 (2024).

14 Coronel-Oliveros, C. *et al.* Gaming expertise induces meso-scale brain plasticity and efficiency mechanisms as revealed by whole-brain modeling. *NeuroImage* **293**, 120633 (2024).

15 Kowalczyk, N. *et al.* Real-time strategy video game experience and structural connectivity–A diffusion tensor imaging study. *Human brain mapping* **39**, 3742-3758 (2018).

16 Rubinov, M. & Sporns, O. Complex network measures of brain connectivity: Uses and interpretations. *NeuroImage* **52**, 1059-1069 (2010). https://doi.org/10.1016/j.neuroimage.2009.10.003

17 Salo, T. *et al.* NiMARE: a neuroimaging meta-analysis research environment. *F1000Research* **7**, 1221-1221 (2018).

18 Xia, M., Wang, J. & He, Y. BrainNet Viewer: A Network Visualization Tool for Human Brain Connectomics. *PLoS ONE* **8**, e68910-e68910 (2013). https://doi.org/10.1371/journal.pone.0068910

19 Gale, D., de Wael, R. V., Benkarim, O. & Bernhardt, B. Surfplot: Publication-ready brain surface figures. *Zenodo* (2021).

20 Alexander-Bloch, A. F. *et al.* On testing for spatial correspondence between maps of human brain structure and function. *Neuroimage* **178**, 540-551 (2018).

21 Burt, J. B., Helmer, M., Shinn, M., Anticevic, A. & Murray, J. D. Generative modeling of brain maps with spatial autocorrelation. *NeuroImage* **220**, 117038 (2020).

22 Goldin, A. P. *et al.* Far transfer to language and math of a short software-based gaming intervention. *Proceedings of the National Academy of Sciences* **111**, 6443-6448 (2014).

23 Moguilner, S. *et al.* Brain clocks capture diversity and disparities in aging and dementia across geographically diverse populations. *Nature Medicine* (2024). https://doi.org/https://doi.org/10.1038/s41591-024-03209-x

24 Tian, Y. E. *et al.* Heterogeneous aging across multiple organ systems and prediction of chronic disease and mortality. *Nature Medicine* **29**, 1221-1231 (2023). https://doi.org/10.1038/s41591-023-02296-6

25 Smith, S. M., Vidaurre, D., Alfaro-Almagro, F., Nichols, T. E. & Miller, K. L. Estimation of brain age delta from brain imaging. *NeuroImage* **200**, 528-539 (2019). https://doi.org/10.1016/j.neuroimage.2019.06.017
26 Engemann, D. A. *et al.* A reusable benchmark of brain-age prediction from M/EEG resting-state signals. *Neuroimage* **262**, 119521 (2022).
27 Yilmaz, D. *et al.* Brain Age Gap Reduction Following Physical Exercise Mirrors Negative Symptom Improvement in Schizophrenia Spectrum Disorders. *medRxiv*, 2025.2001. 2008.24319554 (2025).
28 Pandya, V. A. & Patani, R. Region-specific vulnerability in neurodegeneration: lessons from normal ageing. *Ageing Research Reviews* **67**, 101311 (2021).
29 Khalilian, M. *et al.* Age-related differences in structural and resting-state functional brain network organization across the adult lifespan: A cross-sectional study. *Aging Brain* **5**, 100105 (2024).
30 Filippi, M. *et al.* Age-related vulnerability of the human brain connectome. *Molecular Psychiatry* **28**, 5350-5358 (2023).
31 Kraeutner, S. N. *et al.* Frontoparietal function and underlying structure reflect capacity for motor skill acquisition during healthy aging. *Neurobiology of Aging* **133**, 78-86 (2024).
32 Vuust, P., Heggli, O. A., Friston, K. J. & Kringelbach, M. L. Music in the brain. *Nature Reviews Neuroscience* **23**, 287-305 (2022).
33 Bavelier, D. & Green, C. S. Enhancing Attentional Control: Lessons from Action Video Games. *Neuron* **104**, 147-163 (2019). https://doi.org/10.1016/j.neuron.2019.09.031
34 Küchenhoff, S. *et al.* Visual processing speed is linked to functional connectivity between right frontoparietal and visual networks. *European Journal of Neuroscience* **53**, 3362-3377 (2021). https://doi.org/10.1111/ejn.15206
35 Ptak, R. The Frontoparietal Attention Network of the Human Brain. *The Neuroscientist* **18**, 502-515 (2012). https://doi.org/10.1177/1073858411409051
36 Lewandowska, P. *et al.* Association between real-time strategy video game learning outcomes and pre-training brain white matter structure: preliminary study. *Scientific Reports* **12**, 20741-20741 (2022). https://doi.org/10.1038/s41598-022-25099-0
37 Fields, R. D. A new mechanism of nervous system plasticity: activity-dependent myelination. *Nature Reviews Neuroscience* **16**, 756-767 (2015). https://doi.org/10.1038/nrn4023
38 Münte, T. F., Altenmüller, E. & Jäncke, L. The musician's brain as a model of neuroplasticity. *Nature Reviews Neuroscience* **3**, 473-478 (2002).
39 Schmidt, S. *et al.* Experience-dependent structural plasticity in the adult brain: How the learning brain grows. *NeuroImage* **225**, 117502-117502 (2021). https://doi.org/10.1016/j.neuroimage.2020.117502
40 Sampaio-Baptista, C. & Johansen-Berg, H. White Matter Plasticity in the Adult Brain. *Neuron* **96**, 1239-1251 (2017). https://doi.org/10.1016/j.neuron.2017.11.026
41 Gong, D. *et al.* Action Video Game Experience Related to Altered Large-Scale White Matter Networks. *Neural Plasticity* **2017**, 1-7 (2017). https://doi.org/10.1155/2017/7543686
42 Cantou, P., Platel, H., Desgranges, B. & Groussard, M. How motor, cognitive and musical expertise shapes the brain: Focus on fMRI and EEG resting-state functional connectivity. *Journal of Chemical Neuroanatomy* **89**, 60-68 (2018).
43 Leipold, S., Klein, C. & Jäncke, L. Musical expertise shapes functional and structural brain networks independent of absolute pitch ability. *Journal of Neuroscience* **41**, 2496-2511 (2021).
44 Lynn, C. W. & Bassett, D. S. The physics of brain network structure, function and control. *Nature Reviews Physics* **1**, 318-332 (2019). https://doi.org/10.1038/s42254-019-0040-8
45 Deco, G. & Kringelbach, Morten L. Great Expectations: Using Whole-Brain Computational Connectomics for Understanding Neuropsychiatric Disorders. *Neuron* **84**, 892-905 (2014). https://doi.org/10.1016/j.neuron.2014.08.034
46 Gerstner, W., Sprekeler, H. & Deco, G. Theory and simulation in neuroscience. *science* **338**, 60-65 (2012).
47 Carlos, C.-O., Carsten, G., Vicente, M., Rodrigo, C. & Patricio, O. Whole-brain modeling explains the context-dependent effects of cholinergic neuromodulation. *bioRxiv* (2022).

48 Deery, H. A., Di Paolo, R., Moran, C., Egan, G. F. & Jamadar, S. D. The older adult brain is less modular, more integrated, and less efficient at rest: A systematic review of large-scale resting-state functional brain networks in aging. *Psychophysiology* **60**, e14159 (2023).

49 Damoiseaux, J. S. Effects of aging on functional and structural brain connectivity. *Neuroimage* **160**, 32-40 (2017).

50 Chan, M. Y., Park, D. C., Savalia, N. K., Petersen, S. E. & Wig, G. S. Decreased segregation of brain systems across the healthy adult lifespan. *Proceedings of the National Academy of Sciences* **111**, E4997-E5006 (2014).

51 Dow, R., Warran, K., Letrondo, P. & Fancourt, D. The arts in public health policy: Progress and opportunities. *The Lancet Public Health* **8**, e155-e160 (2023).

52 Ranasinghe, K. G. *et al.* Altered excitatory and inhibitory neuronal subpopulation parameters are distinctly associated with tau and amyloid in Alzheimer's disease. *eLife* **11** (2022). https://doi.org/10.7554/eLife.77850

53 Coronel-Oliveros, C. *et al.* Viscous dynamics associated with hypoexcitation and structural disintegration in neurodegeneration via generative whole-brain modeling. *Alzheimer's & Dementia* (2024). https://doi.org/10.1002/alz.13788

54 Maestú, F., de Haan, W., Busche, M. A. & DeFelipe, J. Neuronal excitation/inhibition imbalance: core element of a translational perspective on Alzheimer pathophysiology. *Ageing Research Reviews* **69**, 101372-101372 (2021). https://doi.org/10.1016/j.arr.2021.101372

55 Lopatina, O. L. *et al.* Excitation/inhibition imbalance and impaired neurogenesis in neurodevelopmental and neurodegenerative disorders. *Reviews in the Neurosciences* **30**, 807-820 (2019). https://doi.org/10.1515/revneuro-2019-0014

56 Martínez-Cañada, P. *et al.* Combining Aperiodic 1/f Slopes and Brain Simulation: An EEG/MEG Proxy Marker of Excitation/Inhibition Imbalance in Alzheimer's Disease. *bioRxiv* (2023). https://doi.org/https://doi.org/10.1101/2022.12.21.521529

57 van den Heuvel, M. P. & Sporns, O. Network hubs in the human brain. *Trends in Cognitive Sciences* **17**, 683-696 (2013). https://doi.org/10.1016/j.tics.2013.09.012

58 van den Heuvel, M. P. & Sporns, O. Rich-Club Organization of the Human Connectome. *Journal of Neuroscience* **31**, 15775-15786 (2011). https://doi.org/10.1523/JNEUROSCI.3539-11.2011

59 Coronel-Oliveros, C., Castro, S., Cofré, R. & Orio, P. Structural Features of the Human Connectome That Facilitate the Switching of Brain Dynamics via Noradrenergic Neuromodulation. *Frontiers in Computational Neuroscience* **15** (2021). https://doi.org/10.3389/fncom.2021.687075

60 Deco, G., Van Hartevelt, T. J., Fernandes, H. M., Stevner, A. & Kringelbach, M. L. The most relevant human brain regions for functional connectivity: Evidence for a dynamical workspace of binding nodes from whole-brain computational modelling. *NeuroImage* **146**, 197-210 (2017). https://doi.org/10.1016/j.neuroimage.2016.10.047

61 Dai, Z. *et al.* Identifying and Mapping Connectivity Patterns of Brain Network Hubs in Alzheimer's Disease. *Cerebral Cortex* **25**, 3723-3742 (2015). https://doi.org/10.1093/cercor/bhu246

62 Moguilner, S. *et al.* Brain clocks capture diversity and disparities in aging and dementia across geographically diverse populations. *Nature medicine* **30**, 3646-3657 (2024). https://doi.org/10.1038/s41591-024-03209-x

63 Deco, G. *et al.* Resting-state functional connectivity emerges from structurally and dynamically shaped slow linear fluctuations. *Journal of Neuroscience* **33**, 11239-11252 (2013).

64 Cohen, S. S., Ngo, C. T., Olson, I. R. & Newcombe, N. S. Pattern separation and pattern completion in early childhood. *Proceedings of the National Academy of Sciences* **122**, e2416985122 (2025).

65 Rakesh, D., McLaughlin, K. A., Sheridan, M., Humphreys, K. L. & Rosen, M. L. Environmental contributions to cognitive development: The role of cognitive stimulation. *Developmental Review* **73**, 101135 (2024).

June, 2025
Manuscript: NCOMMS-24-68019A
Title: Creative experiences and brain clocks

Journal: Nature Communications

RESPONSE TO REVIEWERS
The following point-by-point responses in red have been prepared to address the Reviewers' comments. All additions and modifications made in the revised manuscript are highlighted in yellow. All new references have been incorporated into the References section.

Response to Reviewer #1

Comment 1.1 The revised manuscript is much improved and my prior comments were all appropriately addressed. I commend the authors for their efforts. I do not have any additional concerns about the manuscript. I believe this manuscript provides a high quality contribution to the literature regarding creative experiences and brain age.

Response 1.1: We thank you for the positive feedback.

Response to Reviewer #2

Comment 2.1: The authors have overall made a commendable effort to address the reviewers' comments. There are just a couple of situations in which I feel their response either side-steps a reviewer comment or brings up further questions and I would like to see these clarified before publication:

Response 2.1: We appreciate your positive evaluation and have addressed the remaining point as detailed below.

Comment 2.2: I don't think the response really deals with the issue the reviewer is raising. Removing the gaming group does not remove the fact that these results exist and still point to the alternative hypothesis that cognitively engaging activities are important rather than creativity, necessarily. I think that the active control group does help address this question but also raises its own issues (see point 2).

Response 2.2: Please note that Reviewer 1 already agreed with our previous response, as did not ask for more clarifications. However, we appreciate your point, which we have addressed as follows in the manuscript:

Paragraph 5 in Discussion:

> Despite the heterogeneity of the groups, the findings consistently suggest delayed brain aging across all domains. Moreover, we observed domain-specific associations between artistic performance and brain clock metrics. If other than creativity effects (i.e., cognitively engaging activities) underlie these effects, they likely constitute core components of creative experience that manifest across modalities. Future studies should investigate the underlying subprocesses of creative experiences and their potential distinct effects on brain clocks.

Regarding the active control group, please see **response 2.3** below.

Comment 2.3: The active control group. I am confused by this: why was this group not originally included? Was the group recruited and analyzed especially for the rebuttal? The timing of this would seem unlikely but I suppose it is possible. In any case, a better explanation of the games and justification for why this game is less creative is needed: really the idea that creativity in itself is what is important rests on this very small (N=12) control group and this should be included as a major limitation with acknowledgement that cognitive stimulation is an alternative explanation. Additionally, given the inclusion of a control group, it is now possible to use statistical tests that are recommended for assessing change vs. a control group (controlling for baseline differences).

Showing change in each group in isolation is not typically considered robust for this kind of design.

Response 2.3: Thank you for pointing this out. The group was not recruited ad hoc for the revision but was part of the original study design. We didn't include it because we originally focused on the pre- and post-measures. We have clarified all remaining points as follows:

Methods: Study 2. Pre/post-learning design:

> We included an active control group (N = 12) trained in Hearthstone. This group was part of the original study design[1], with identical recruitment, training time, and inclusion/exclusion criteria as the StarCraft II group. The Hearthstone game was selected due to its more rule-based and turn-based mechanics, with limited improvisation and creative play compared to StarCraft II's real-time decision-making[1].

Paragraph 5 in Discussion:

> An additional limitation of our work is the small sample size used for the pre/post-learning design, and, especially, the active control group. Although both results convergently showed the effects of the intervention, the limited sample size could under/overestimate the magnitude of the outcomes.

In addition, we now include a direct comparison between learners and active control groups, as you recommended:

Results: Higher levels of creative experiences are associated with delayed brain age:

> These effects were specific to StarCraft II itself, as we did not find any differences in the active control of the pre/post design (ΔBAGs = 0.057, t = 0.979, p = 0.0274, D = 0.0092, FDR-corrected, **Figure S11** in **Supplementary Materials**). Conversely, significant differences were observed when comparing learners and active controls (Δ(ΔBAGs) = -3.152, U = 90, p = 0.0362, D = -0.49, **Table S11** in **Supplementary Materials**). Thus, results showed moderate to large effects of creativity expertise on BAGs, and small to moderate effects of short-term learning.

Supplementary Materials: Supplementary Table S11:

Table S11. BAG differences in the pre/post-learning design.

BAG differences (pre/post-learning design)				
**Group**	**ΔBAGs (years)**	**U**	**p**	**D**
Learners	-3.115 (5.12)	90	0.0362	-0.49
Active control	0.057 (6.88)			

ΔBAGs represent the difference, in terms of BAGs, between the post- and pre-training conditions, with the standard deviation in parentheses. Significantly delayed brain age was observed in the learners in comparison with the active control group: one-sided Mann-Whitney test ($\Delta(\Delta BAGs) = -3.152$, $U = 90$, $p = 0.0362$, D = -0.49).

Comment 2.4: It also seems important to me to better clarify exactly what cognitive process is being measured via the "blind paradigm" (should this be "blink"?) and whether you think this should relate to the Brain Age Gaps and why (also do they relate?).

Response 2.4: We appreciate the catch. This was a typo and has been corrected to "attentional blink" and we have clarified the point as follows:

Methods: Study 2. Pre/post-learning design

… This task was included to assess generalized attention and temporal processing improvements post-training. Although not directly associated with BAGs, improvements in attentional blink performance may reflect learning-induced changes[2].

Comment 2.5 (code): As a separate point I tried to clone and run the code but I am having trouble with the "plot_violins" module (error: there is no module named 'plot_violins'). Is this a script you have written that is not available on the GitHub or it is from a larger module that is included in the dependencies (I have installed all dependencies successfully)? This might be my mistake but please elaborate in your readme to make it easier for others to run this code.

Response 2.5: Thank you for bringing this to our attention. The plot_violins.py script was inadvertently omitted from the GitHub repository. We have now added this module and updated the **README** file to clarify its usage and provide step-by-step instructions to reproduce the plots.

Comment 2.6: I am not an expert at coding and have not used all of the packages provided here. A brief examination suggests the code exists and the readme is sufficient if not amazing. Similar with the commenting: not the best I've seen but not terrible. I am having an issue running the code due to the "plot_violins" module and have asked the authors to further clarify where this is coming from.

Response 2.6: We have now amended the violin plot code issues (**response 2.5** above).

Response to Reviewer #3

Comment 3.1: I appreciate the authors' thoughtful and thorough revisions. They have addressed all of the concerns I previously raised. In particular, I commend the rigorous effort put into the additional sensitivity analyses, which clearly demonstrate the robustness of the findings.

I believe the manuscript is now suitable for publication and have no further comments or requests.

I have reviewed the Git repository prepared by the authors. While I have not executed the code, the repository appears to be well-organized and includes adequate documentation and supporting data. In my view, it provides sufficient detail to allow for future replication and reuse.

Response 3.1: Thanks for your careful reading and constructive input throughout the process. We have amended the violin plot code issues as detailed in **response 2.5**.

Response to Reviewer #4

Comment 4.1: I co-reviewed this manuscript with one of the reviewers who provided the listed reports. This is part of the Nature Communications initiative to facilitate training in peer review and to provide appropriate recognition for Early Career Researchers who co-review manuscripts.

Response 4.1: We are grateful for your involvement and acknowledge the collaborative effort in the peer-review process.

Response to Reviewer #5

Comment 5.1: This manuscript presents an innovative and ambitious study linking creative expertise and learning to delayed brain aging, as measured by EEG/MEG-based brain clocks. While I have a generally positive impression of the work, I have limited my assessment to the methodological aspects, particularly those involving M/EEG source reconstruction and connectivity analysis. The authors use standard and appropriate preprocessing and source localization techniques for both EEG and MEG. The use of sLORETA and beamforming represents a robust and widely accepted approach to solving the inverse problem, providing a valid pipeline to estimate neural activity at the source level. However, my main concerns relate to how this source-level activity is used to infer functional connectivity (FC) and, by extension, brain age gaps (BAGs). Several methodological choices which, although not necessarily incorrect, lack justification and their limitations are not acknowledged. As these choices do not invalidate the present results, I recommend publication of this manuscript, once all these aspects are properly addressed:

Response 5.1: Thanks for your detailed evaluation and constructive input throughout the process. We have addressed all your points as detailed below.

Comment 5.2: Choice of Features and Frequency Range. There are many features derived from EEG/MEG data that correlate with brain age (e.g., power spectral density, entropy, kurtosis, etc.; see Engemann et al., NeuroImage, 2022). The authors' decision to rely exclusively on functional connectivity in the 8–40 Hz band should be justified. Why was this specific frequency range chosen, and why were other features or narrower bands not considered?

Response 5.2: Thanks for your valuable observation. We have clarified this point as follows:

Methods. M/EEG acquisition, preprocessing, and connectivity:

> The 8–40 Hz band was selected to cover alpha to low-gamma rhythms, which are linked to creative and attentional processes[3-7] as well as brain age[8,9]. Although many features derived from M/EEG correlate with brain age (e.g., power spectral density, entropy, kurtosis)[10], we focused on functional connectivity for specific reasons[11]. Functional connectivity is a robust marker for assessing brain aging, particularly when considering diverse datasets[8,9,11]. It also allows for comparability with fMRI-based brain clock estimations[11]. By using a mesh model and a common source space, we ensured standardized brain mapping across participants[11]. This approach offers a balanced trade-off between spatial and temporal

resolution, which is important in multi-site data. In any case, future studies should explore additional M/EEG-derived metrics[10] to further enrich brain age modeling.

Comment 5.3: Use of Pearson Correlation for Functional Connectivity. My second concern relates to the use of Pearson correlation as the metric for functional connectivity. While it can provide a rough approximation of connectivity, it has well-known limitations that are not acknowledged in the manuscript. The most significant issue is volume conduction: high correlations between brain regions may simply reflect shared signal sources rather than genuine functional interactions. This can lead to inflated or misleading connectivity estimates. The choice of Pearson correlation over more robust alternatives—such as imaginary coherence, phase-lag index, or orthogonalized connectivity measures—is not justified. A critical discussion of this decision, and its implications for the interpretation of the results, would be necessary. For a review of functional connectivity maps in EEG and MEG, see van Diessen et al., Clinical Neurophysiology, 2015.

Response 5.3: We appreciate your observation and have addressed it as follows:

Paragraph 6 in Discussion:

We used Pearson correlation for its interpretability and reproducibility across different modalities. Source localization reduces, but does not eliminate, volume conduction and spatial leakage, improving the reliability of correlation-based connectivity relative to sensor-level analyses[12,13]. However, as Pearson's correlation is susceptible to volume conduction artifacts[14], future works should be replicated using alternative connectivity metrics[15,16] (e.g., imaginary coherence, phase-lag index, or high-order interactions) to ensure the robustness of findings.

Comment 5.4: Sensor Density and Source Reconstruction Accuracy. The accuracy of source-level functional connectivity estimates is highly dependent on the number of recording sensors. Estimating activity from 78 cortical regions using only 32 EEG channels may lead to substantial spatial leakage and inflated correlations due to low spatial resolution. Have the authors assessed whether FC estimates — or BAGs — are systematically higher or more variable in datasets recorded with fewer sensors (e.g., 32 vs. 264 channels)? This is especially relevant given the multi-site nature of the dataset.

Response 5.4: Thanks for your point. We clarified our approach to account for differences in electrode count and added an analysis comparing the slopes of predicted vs. chronological age for both 64- and 128-channel data. Similar slopes indicate consistent model performance, and no significant differences were found, suggesting that sensor density did not bias predictions. This has been added as a sensitivity analysis in the revised manuscript, where we made the following changes:

Results. Sensitivity analyses

We observed no meaningful differences in prediction slopes between 64- and 128-channel EEG data in either the training or creativity datasets. Results are reported in **Supplementary Table S10.**

Supplementary Table S10

Table S10. Comparison of prediction slopes by EEG sensor density.

Training data			
**Channels**	**Slope**	**t values**	**p**
64	0.922 (0.002)	1.27	0.213
128	0.921 (0.003)		

Creativity data			
**Channels**	**Slope**	**t values**	**p**
64	1.746 (0.0344)	-1.28	0.211
128	1.764 (0.0395)		

Slopes of predicted versus chronological age were compared between 64- and 128-channel EEG data in training and creativity datasets.

Methods. Sensitivity analyses

> To assess the impact of sensor density on model performance, we computed the slope of predicted versus chronological age using linear regression, separately for 64- and 128-channel EEG datasets, in both the training and creativity datasets.

Further, we have clarified the specific control procedures to address any remaining effects of channel density in the Discussion of the manuscript.

Paragraph 6 in Discussion:

> Another limitation was the use of EEG data recorded with different numbers of electrodes, as lower sensor density may increase signal leakage and distort connectivity estimates[17]. However, our sensitivity analyses showed that electrode count did not affect the brain age models. Compared to raw functional connectivity measures, BAGs provide coarser-grained estimates that are more robust to sensor density variations. In previous work[11], BAG estimates remained stable across EEG systems, channel counts, and signal quality when our data harmonization was applied. Importantly, each dataset in the creativity study included its own control group, ensuring that differences between experts and non-experts or pre- and post-learning were not confounded by electrode numbers.

Minor comments

Comment 5.5: participant numbers: The manuscript should include the number of participants contributed by each center or dataset, ideally in the main table. This would help assess the potential for site-specific effects or sampling bias.

Response 5.5: Thank you. We have now added the number of participants contributed by each dataset in **Table 1,** and refer to this in the Methods section. We provided here a section of the table with the countries' samples.

Table 1. Demographic data.

Group	Subgroup	Countries	Sample size	Age (years)	Education (years)	Sex (female: male ratio)	Expertise criteria	Expertise level
Training data	Global south	Argentina	37	46.0 (21.0)	13.8 (4.4)	603:593*	Not apply	Not apply
		Brazil	84					
		Chile	41					
		Colombia	134					
		Cuba	220					
	Global north	Greece	22					
		Ireland	132					
		Italy	20					
		Turkey	484					
		UK	66					
		**Total**	**1240**					

Comment 5.6: line 460: "Exclusion criteria included normal or corrected-to-normal vision..." — this should not be listed as an exclusion.

Response 5.6: Thank you for catching this. We have corrected the sentence to clarify that normal or corrected-to-normal vision was an inclusion criterion, not an exclusion.

Methods. Tango dancers

Exclusion criteria included no past neurological or psychiatric history reported. Inclusion criteria included being right-handed, verified by the Edinburgh Inventory, with normal or corrected-to-normal vision.

References

1 Jakubowska, N. *et al.* The role of individual differences in attentional blink phenomenon and real-time-strategy game proficiency. *Heliyon* **7** (2021).

2 Raymond, J. E., Shapiro, K. L. & Arnell, K. M. Temporary suppression of visual processing in an RSVP task: An attentional blink? *Journal of experimental psychology: Human perception and performance* **18**, 849 (1992).

3 Orgs, G., Dombrowski, J. H., Heil, M. & Jansen-Osmann, P. Expertise in dance modulates alpha/beta event-related desynchronization during action observation. *European Journal of Neuroscience* **27**, 3380-3384 (2008).

4 Jauk, E., Benedek, M. & Neubauer, A. C. Tackling creativity at its roots: Evidence for different patterns of EEG alpha activity related to convergent and divergent modes of task processing. *International Journal of Psychophysiology* **84**, 219-225 (2012).

5 Başar, E., Başar-Eroglu, C., Karakaş, S. & Schürmann, M. Gamma, alpha, delta, and theta oscillations govern cognitive processes. *International journal of psychophysiology* **39**, 241-248 (2001).

6 Herrmann, C. S., Fründ, I. & Lenz, D. Human gamma-band activity: a review on cognitive and behavioral correlates and network models. *Neuroscience & Biobehavioral Reviews* **34**, 981-992 (2010).

7 Seabra, J. P., Kaltwasser, L., Mylius, M. & Gallese, V. Artistic expertise shapes face perception: An alpha frequency and N170 study on portraiture. *Neuroimage: Reports* **2**, 100117 (2022). https://doi.org/https://doi.org/10.1016/j.ynirp.2022.100117

8 Baez, S. *et al.* Structural inequality and temporal brain dynamics across diverse samples. *Clinical and Translational Medicine* **14**, e70032 (2024). https://doi.org/https://doi.org/10.1002/ctm2.70032

9 Hernandez, H. *et al.* Brain health in diverse settings: How age, demographics and cognition shape brain function. *NeuroImage* **295**, 120636 (2024).

10 Engemann, D. A. *et al.* A reusable benchmark of brain-age prediction from M/EEG resting-state signals. *Neuroimage* **262**, 119521 (2022).

11 Moguilner, S. *et al.* Brain clocks capture diversity and disparities in aging and dementia across geographically diverse populations. *Nature medicine* (2024). https://doi.org/https://doi.org/10.1038/s41591-024-03209-x

12 Palva, J. M. *et al.* Ghost interactions in MEG/EEG source space: A note of caution on inter-areal coupling measures. *Neuroimage* **173**, 632-643 (2018).

13 Gross, J. *et al.* Good practice for conducting and reporting MEG research. *Neuroimage* **65**, 349-363 (2013).

14 Van Diessen, E. *et al.* Opportunities and methodological challenges in EEG and MEG resting state functional brain network research. *Clinical Neurophysiology* **126**, 1468-1481 (2015).

15 Kida, T., Tanaka, E. & Kakigi, R. Multi-Dimensional Dynamics of Human Electromagnetic Brain Activity. *Frontiers in Human Neuroscience* **9** (2016). https://doi.org/10.3389/fnhum.2015.00713

16 Herzog, R. *et al.* Genuine high-order interactions in brain networks and neurodegeneration. *Neurobiology of Disease* **175**, 105918-105918 (2022). https://doi.org/10.1016/j.nbd.2022.105918

17 Allouch, S. *et al.* Effect of channel density, inverse solutions and connectivity measures on EEG resting-state networks reconstruction: A simulation study. *NeuroImage* **271**, 120006 (2023).

July, 2025
Manuscript: NCOMMS-24-68019A
Title: Creative experiences and brain clocks

Journal: Nature Communications

RESPONSE TO REVIEWERS
The following point-by-point responses in red have been prepared to address the Reviewers' comments. All additions and modifications made in the revised manuscript are highlighted in yellow.

Response to Reviewer #2

Comment 2.1 Thank you for further addressing my comments. Although the analysis chosen for comparing active and control groups is unusual I do not think it requires an additional round of revisions by itself. I think I have requested sufficient revisions at this point and the authors have done a commendable job of addressing them.

Response 2.1: We thank you for the positive feedback.

Comment 2.2: I will note what I think is a typo: "These effects were specific to StarCraft II itself, as we did not find any differences in the active control of the pre/post design (ΔBAGs = 0.057, t = 0.979, p = 0.0274, D = 0.0092, FDR-corrected, Figure S11 in Supplementary Materials)." this p-value seems unlikely for the t-statistic and effect sizes shown and appears to be significant... I am wondering if it is a typo.

Response 2.2: Thanks for catching this typo. The t-values and p-values were inverted. We have corrected this error in the manuscript:

Results. Higher levels of creative experiences are associated with delayed brain age:

> (ΔBAGs = 0.057, t = 0.0274, p = 0.979, D = 0.0092, FDR-corrected, **Figure S11** in **Supplementary Materials**).

Comment 2.3: The code still doesn't run for me. Thank you to the authors for adding plot_violins.py: the script successfully gets past this point now but fails at importing from surfplot. Maybe another custom plotting script that needs to be added?

Response 2.3: Thanks for raising this issue. The library surfplot is one of the dependencies that should be installed for completely running the code. We have now clarified this within the README file of the Repo.

Response to Reviewer #5

Comment 5.1: The authors have overall made a commendable effort to address the reviewers' comments. There are just a couple of situations in which I feel their response either side-steps a reviewer comment or brings up further questions and I would like to see these clarified before publication:

Response 5.1: We appreciate your positive evaluation and feedback.
